# Al_2_O_3_/Ag nanostructured substrates prepared by ALD/CVD for matrix-free laser desorption/ionization mass spectrometry

**DOI:** 10.1039/d5ra09570k

**Published:** 2026-05-20

**Authors:** Jagoda Pałczyńska, Ewelina Sibińska, Piotr Piszczek, Kinga Robotnik, Aleksandra Radtke, Oleksandra Pryshchepa, Marek Trzcinski, Weronika Brzozowska, Dominika Przybysz, Paweł Pomastowski

**Affiliations:** a Centre for Modern Interdisciplinary Technologies, Nicolaus Copernicus University in Toruń Wileńska 4 87-100 Toruń Poland 503551@doktorant.umk.pl p.pomastowski@umk.pl; b Department of Inorganic and Coordination Chemistry, Faculty of Chemistry, Nicolaus Copernicus University in Toruń Gagarina 7 87-100 Toruń Poland; c Division of Surface Science, Faculty of Chemical Technology and Engineering, Bydgoszcz University of Science and Technology Al. Prof. S. Kaliskiego 7 85-796 Bydgoszcz Poland

## Abstract

Nanostructure-assisted laser desorption/ionization (NALDI) is a promising matrix-free alternative to matrix-assisted laser desorption/ionization (MALDI) for the analysis of low-molecular-weight compounds. Here, we report the fabrication of NALDI substrates composed of atomic-layer-deposited Al_2_O_3_ interlayers on AISI 304 steel followed by chemical-vapor-deposited silver nanoparticles (AgNPs). Three independently prepared substrates were used to evaluate the reproducibility of the ALD/CVD approach. Surface morphology, composition, and optical properties were examined by scanning electron microscopy with energy-dispersive X-ray spectroscopy (SEM/EDX), atomic force microscopy (AFM), X-ray photoelectron spectroscopy (XPS), and UV-vis diffuse reflectance spectroscopy (UV-vis DRS). SEM and AFM showed granular AgNP layers with mean particle sizes of 31 ± 13, 39 ± 19, and 51 ± 26 nm for plates 1–3, indicating that the initial substrate topography strongly affected the final nanostructure. XPS confirmed the presence of metallic silver, whereas UV-vis diffuse reflectance spectra exhibited a plasmon-related band at approximately 420 nm. The resulting Al_2_O_3_/Ag substrates were then evaluated in positive-ion NALDI MS using methionine, serine, lactose, and arabinose as model analytes. Amino acids produced protonated ions, metal-ion adducts, and fragment ions, with methionine and serine fragments remaining detectable down to 8 and 20 µg mL^−1^, respectively. Saccharides showed extensive fragmentation, and characteristic fragment ions were observed over the full concentration range examined (100–0.05 µg mL^−1^). The results demonstrate the feasibility of combining ALD-grown Al_2_O_3_ with CVD-grown AgNPs to generate matrix-free LDI substrates, while also showing that improved surface homogeneity will be necessary to enhance reproducibility and analytical sensitivity.

## Abstract 2

Al_2_O_3_/Ag nanostructured substrates prepared by ALD/CVD are presented as matrix-free platforms for laser desorption/ionization mass spectrometric analysis of low-molecular-weight compounds.

## Graphical abstract

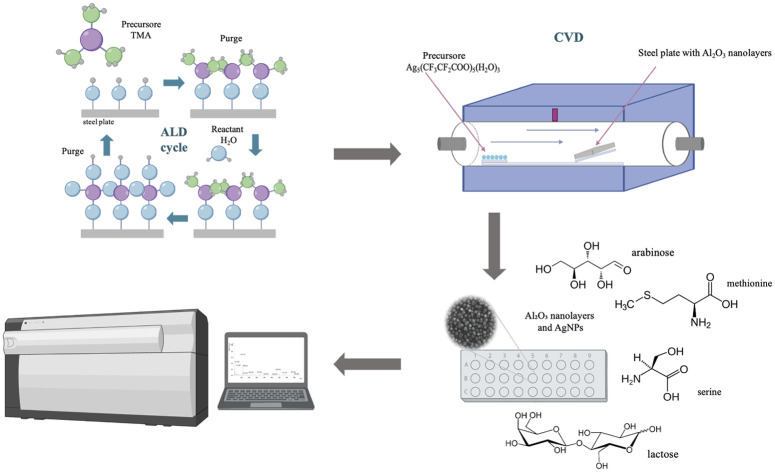

## 1 Introduction

Laser desorption/ionization (LDI) techniques are central to modern mass spectrometry, as they enable the analysis of chemically diverse compounds with simple sample preparation under soft ionization conditions that favor preservation of molecular ions and minimize fragmentation.^1^ Among these methods, matrix-assisted laser desorption/ionization time-of-flight mass spectrometry (MALDI-TOF MS) is widely used for the analysis of macromolecules such as proteins, peptides, nucleic acids, and polymers.^2–5^ However, the use of organic matrices imposes several well-recognized limitations. In particular, the analysis of low-molecular-weight compounds (<700 *m*/*z*) is hampered by intense background signals originating from the matrix, which interfere with analyte peaks. Furthermore, the lack of a universal matrix requires prior knowledge of the analyte class, and heterogeneous co-crystallization of the matrix with the analyte deteriorates quantitative performance.^6^ These drawbacks have motivated intensive efforts to develop matrix-free LDI approaches, including nanostructure-assisted laser desorption/ionization (NALDI). NALDI employs solid substrates coated with metal nanostructures, such as gold or silver nanoparticles (AuNPs, AgNPs), whose unique optical and plasmonic properties promote efficient absorption of laser energy and thereby facilitate analyte desorption and ionization.^7^ This technique has been successfully applied to the analysis of carboxylic acids,^8^ lipids,^9^ and drug metabolites,^10^ as well as to the differentiation of *Escherichia coli* strains resistant and susceptible to cefotaxime.^11^ The growing interest in nanoparticle-based LDI has stimulated rapid advances in methods for nanoparticle synthesis, stabilization, and characterization, with the goal of developing reproducible, well-controlled protocols that yield homogeneous and stable materials.^7^ Our previous work has shown that chemical vapor deposition (CVD) is a particularly promising route to AgNPs suitable for LDI analysis of low-molecular-weight compounds.^7,9,11,12^ To optimize surface properties for the adhesion and growth of deposited nanomaterials, substrates typically require dedicated preparation and activation steps. These may include cleaning, etching, or deposition of an interlayer designed to remove contaminants and introduce active nucleation centers that promote uniform layer growth.^13^ Despite such multi-step procedures, structural defects in the underlying substrate often remain a challenge. Mechanical treatment (grinding, polishing) generates scratches, grooves, and ridges, while ion etching can further enhance roughness due to phase-dependent etch rates, leading to micro-roughness and crater formation.^14^ These defects directly affect the topography of the deposited layers and, in turn, the reproducibility of the deposition process, the uniformity of nanoparticle distribution, and the resulting surface properties. Moreover, heterogeneous distribution of alloying elements in steel and local variations in chemical composition and surface energy can cause non-uniform precursor adsorption during deposition, giving rise to irregular nanostructure growth. To mitigate such substrate-derived defects, we employed Al_2_O_3_ nanolayers obtained by atomic layer deposition (ALD) as an intermediate layer between the steel substrate and the AgNPs. Aluminum oxide is widely used in surface engineering because of its high chemical and thermal stability, favorable dielectric properties, and ability to form adherent, conformal coatings on a variety of substrates, including metals.^15^ Introducing a controlled Al_2_O_3_ interlayer is expected to provide a more uniform and reproducible platform for AgNP nucleation and growth. Uniformity and reproducibility of nanoparticle distribution are crucial in NALDI: heterogeneous surface morphology leads to local variations in laser energy absorption and ionization efficiency, which directly affect the quality and reproducibility of the acquired spectra.^7,16^ Against this background, the aim of this study was to fabricate Al_2_O_3_/Ag nanostructured substrates by combining ALD and CVD, to examine how substrate topography propagates through the deposition sequence and affects the resulting AgNP morphology, and to assess the suitability of the obtained materials for matrix-free LDI MS analysis of selected low-molecular-weight model compounds.

## 2 Methodology

### 2.1 Substrate preparation

The substrates were prepared using two thin-film deposition processes. Al_2_O_3_ layers were deposited *via* ALD, and subsequently, the aluminum oxide nanolayers were coated with silver nanoparticles using CVD.

#### 2.1.1 Synthesis of Al_2_O_3_ nanolayers

Aluminum oxide layers were synthesized on clean stainless steel substrates (AISI 304, EN 1.4301) subjected to surface cleaning and activation prior to deposition. Initially, the substrates were ultrasonically cleaned using a mixture of acetonitrile and ethanol (1 : 1 v/v, purity >99.95%). Subsequently, the substrates were rinsed twice with distilled water and dried under an Ar stream. The surface was activated by immersion in a 1% trifluoroacetic acid (TFA) solution.

Aluminum oxide layers were deposited using a Beneq TFS 200 302 reactor (Espoo, Finland; manufactured in 2021). Trimethylaluminum (TMA, Al(CH_3_)_3_) and water (H_2_O) were used as precursors. The process was conducted at a chamber temperature of 200 °C. The pulse duration was 125 ms for TMA and 130 ms for water. A nitrogen purge step of 760 ms was applied after each precursor pulse. The total number of deposition cycles was 1500.

#### 2.1.2 Synthesis of silver nanoparticles

The substrates coated with aluminum oxide were dried under an Ar stream and subsequently placed in a custom-built hot-wall CVD reactor for the deposition of AgNPs. 100 µg of silver(i) pentafluoropropionate trihydrate ([Ag_5_(O_2_CC_2_F_5_)_5_(H_2_O)_3_]) was used as the precursor, per deposition run, corresponding to 0.355 µmol of Ag. The process was conducted at a precursor vaporization temperature of 240 °C and a decomposition and deposition temperature of 290 °C. Argon (Ar) was used as the carrier gas, and the total pressure in the reactor was 3.0 mbar. The deposition time was 60 min. The substrates were stored in a sealed, dry container protected from light. A detailed description and schematic of the reactor are provided in the SI.

### 2.2 Characterization of the obtained substrates

**Fig. 1:**
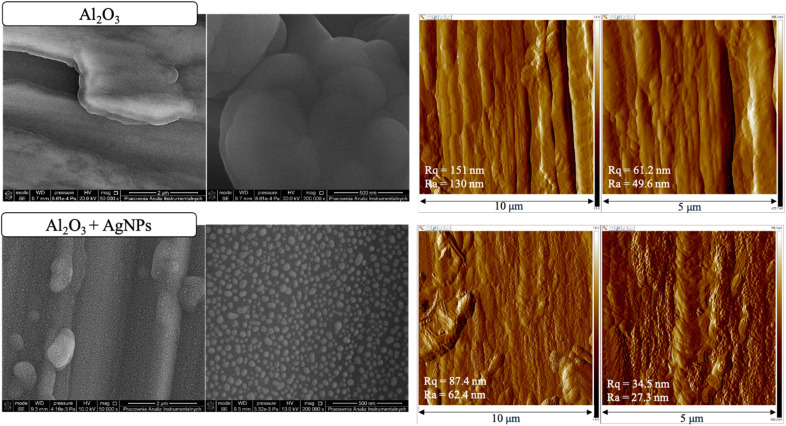
Comparison of the morphology and topography of NALDI plate no. 1. SEM images (500 00×, 2 000 00×) and AFM maps (10 µm, 5 µm) of the surface: top – Al_2_O_3_-coated steel substrate before Ag deposition, bottom – Al_2_O_3_ layer coated with silver nanoparticles (Al_2_O_3_ + AgNPs). Corresponding data for plates 2 and 3 are shown in SI Fig. S2 and S3.

Three independently prepared AISI 304 stainless steel substrates (1 × 1 cm) were first coated with Al_2_O_3_ by ALD and then characterized after each preparation stage, *i.e.* as Al_2_O_3_-coated steel substrates and, subsequently, as Al_2_O_3_/Ag-coated steel substrates after CVD deposition of AgNPs. In addition, an Al_2_O_3_-coated Si chip and unmodified AISI 304 stainless steel were analyzed as reference samples. The resulting coatings were examined by scanning electron microscopy coupled with energy-dispersive X-ray spectroscopy (SEM/EDX), atomic force microscopy (AFM), X-ray photoelectron spectroscopy (XPS), and UV-vis diffuse reflectance spectroscopy (UV-vis DRS). SEM imaging and EDX analyses were performed using a LEO Electron Microscopy Ltd 1430 VP scanning electron microscope equipped with a Quantax 200 energy-dispersive X-ray spectrometer and an XFlash 4010 detector (Bruker AXS). The acquisition parameters were adjusted depending on the imaging and analytical purpose. The successively higher-magnification SEM images shown in Fig. 1 and SI Fig. S2 and S3 were acquired from the same area of the sample. EDX measurements were carried out at an accelerating voltage of 28.0 kV. XPS measurements were performed using an Al Kα X-ray source (1486.7 eV) under high-vacuum conditions (∼1 × 10^−9^ mbar). The binding energy scale was calibrated with respect to the C 1s peak at 284.8 eV. Spectra were processed using CasaXPS software with Shirley background subtraction and mixed Gaussian–Lorentzian peak fitting. An asymmetric line shape was applied for metallic states where appropriate. AFM measurements were carried out using a NanoScope MultiMode SPM system (Veeco, Digital Instruments) equipped with a NanoScope IIIa controller, a MultiMode head, and an E scanner with a maximum scan area of 10 × 10 × 2.5 µm. The measurements were performed in intermittent-contact mode (Tapping Mode) using an HA_NC Etalon probe (ScanSens GmbH). The probe consisted of a silicon body, a polysilicon cantilever, and a high-resolution silicon tip, with a chip size of 3.6 × 1.6 × 0.45 mm. The cantilever used (no. 2) had a length of 124 µm, a width of 34 µm, a thickness of 1.85 µm, a resonant frequency of 140 kHz, and a force constant of 3.5 N m^−1^. AFM data were recorded using NanoScope software (version V530r3sr3). For the reference samples (Si chip coated with an Al_2_O_3_ layer and AISI 304 stainless steel), X-ray photoelectron spectroscopy measurements were carried out under a base pressure of 5 × 10^−10^ mbar using an Al Kα X-ray source (1486.6 eV) oriented at an angle of 55° to the sample normal. Photoelectron spectra were recorded with a VG-Scienta R3000 analyzer using an energy step of 100 meV. The experimental data were processed in CasaXPS software using Shirley background subtraction and Gaussian–Lorentzian peak fitting. AFM measurements were performed in tapping mode using an OTESPA-R4 probe on a Bruker Innova microscope.

### 2.3 Sample preparation

Amino acid standards (methionine and serine; Sigma-Aldrich) and saccharide standards (lactose and arabinose; Sigma-Aldrich) were used for the analysis. The powdered standards were dissolved in water to obtain stock solutions at a concentration of 1 mg mL^−1^. Lower concentrations were prepared by serial dilution. The analyses were performed at concentrations of 200, 100, 40, 20, 8, and 4 µg mL^−1^ for amino acids, and 100, 50, 10, 5, 1, and 0.05 µg mL^−1^ for saccharides. A volume of 0.5 µL of each solution was applied onto the nanoparticle-coated steel substrate. After spotting, the samples were allowed to dry in air at room temperature and were then introduced into the mass spectrometer for analysis (Table 1).

**Table 1:** List of analyzed standards

Compound formula	Molar mass	Initial concentration	Solvent
Methionine C_5_H_11_NO_2_S	149.21	1 mg mL^−1^	Water
Serine C_3_H_7_NO_3_	105.09	1 mg mL^−1^	Water
Lactose C_12_H_22_O_11_	342.30	1 mg mL^−1^	Water
Arabinose C_5_H_10_O_5_	150.13	1 mg mL^−1^	Water

### 2.4 Analysis of samples using NALDI MS

Mass spectrometry experiments in LDI mode were performed in positive reflectron mode (ion source 1 : 25.05 kV; ion source 2 : 22.40 kV) using a Bruker ultrafleXtreme II spectrometer equipped with a SmartBeam II laser (355 nm, 2 kHz). Each sample spot was subjected to a total of 6000 laser shots, distributed among four symmetrically arranged points around the center of the spot. At each point, 1500 laser shots were accumulated using the default random walk strategy (random points with 50 laser shots each).

The measurement range covered a mass-to-charge ratio (*m*/*z*) of 40–2000, with signal suppression typically applied for ions with *m*/*z* below 40. Data were calibrated and analyzed using FlexAnalysis software (version 3.3) employing the centroid peak detection algorithm. Mass calibration for the plates coated with silver nanoparticles was performed using internal standards utilizing signals from ^107^Ag and ^109^Ag ions and their Ag_2_ and Ag_3_ clusters (quadratic mode). The parameters were as follows: laser power 30%, detector gain set to 30×, global attenuator value 45%, laser focus parameter set to “large”, digitizer sensitivity 100 mV, reflectron analog offset 2.6 mV, and trigger level 800 mV.

Three spectra were recorded for each sample dilution in the analyses. Substrates with Al_2_O_3_/Ag nanostructures were analyzed using an MTP Slide Adapter II (Bruker Daltonics, Bremen, Germany).

## 3 Results

### 3.1 Characterization of deposited layers

**Fig. 2:**
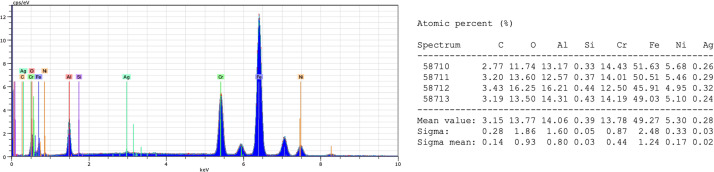
EDX analysis of plate 1. The EDX analysis results for plates 2 and 3 are provided in the (SI Fig. S4 and S5).

An aluminum oxide layer was sequentially deposited on three independent steel substrates *via* the ALD method, followed by the deposition of silver nanoparticles *via* the CVD method. The substrates were characterized after each stage. The obtained SEM images (Fig. 1 and SI Fig. S2 and S3) revealed that the Al_2_O_3_ nanolayers exhibited a heterogeneous morphology, with the presence of rounded forms and local thickenings. In turn, SEM images of the surface after silver deposition (Fig. 1 and SI Fig. S2 and S3) revealed a granular morphology with clearly visible, approximately spherical silver nanoparticles distributed over the surface. The average nanoparticle size was 31 ± 13 nm for plate 1, 39 ± 19 nm for plate 2, and 51 ± 26 nm for plate 3, based on the analysis of 30 particles for each plate. Images obtained using atomic force microscopy (Fig. 1 and SI Fig. S2 and S3) further confirmed the morphology of the analyzed substrates. Roughness parameter values (*R*_a_ and *R*_q_) were determined for each plate. After AgNPs deposition, the surface exhibited a more granular topography. For plates 1 and 3, the roughness decreased after AgNPs deposition, whereas plate 2 exhibited the opposite trend. For plates 1 and 3, *R*_a_ decreased from 49.6 to 27.3 nm and from 35.2 to 23.0 nm, respectively, whereas for plate 2 it increased from 19.7 to 26.3 nm. SEM–EDX analysis (Fig. 2 and SI S4, S5) detected Al-, O-, and Ag-related signals for all three plates, consistent with deposition of the Al_2_O_3_ interlayer and Ag-containing surface structures; however, under the applied EDX conditions, contributions from the underlying steel substrate must also be taken into account.

**Fig. 3:**
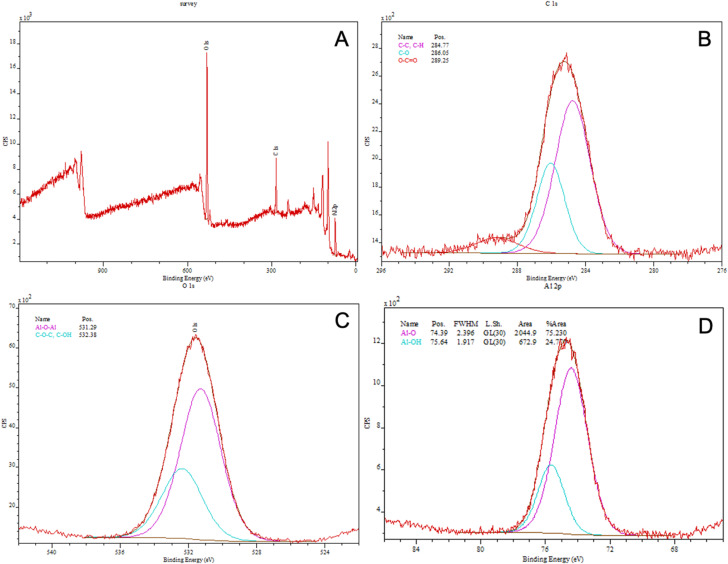
XPS characterization of the Al_2_O_3_ sample: (A) survey spectrum; (B) high-resolution C 1s spectrum; (C) high-resolution O 1s spectrum; and (D) high-resolution Al 2p spectrum. The XPS data for plates 2 and 3 are shown in the SI (Fig. S7 and S9).

XPS spectra for the Al_2_O_3_ sample (Fig. 3) revealed the presence of O, C, and Al elements (Table 2). In the O 1s region, two components with binding energies of 531.3 eV and 532.4 eV were identified, corresponding to metal oxide species (Al–O) and oxygen-containing surface species (C–O–C/C–OH), respectively. The C 1s spectrum exhibits three characteristic peaks at 284.8 eV (C–C/C=C), 286.1 eV (C–O), and 289.2 eV (O–C=O), typical of carbonaceous species present on the surface. The high-resolution Al 2p spectrum consists of two components at 74.4 eV and 75.6 eV, corresponding respectively to Al–O and Al–OH species, characteristic of an aluminum oxide-based surface layer (Table 3).

**Table 2:** Surface elemental composition of the Al_2_O_3_-coated sample determined by XPS survey analysis

% Al	% O	% C
30.50	40.21	29.29

**Table 3:** Peak positions, assignments, and surface concentrations of the components identified in the high-resolution XPS spectra of the Al_2_O_3_-coated sample

Region	Binding energy (eV)	Assignment	Surface concentration (%)
C 1s	284.77	C–C/C=C	18.42
C 1s	286.05	C–O	8.86
C 1s	289.25	O–C=O	2.19
O 1s	531.29	Metal oxides	27.48
O 1s	532.38	C–O–C/C–OH	12.73
Al 2p	74.39	Al–O	22.95
Al 2p	75.64	Al–OH	7.55

**Fig. 4:**
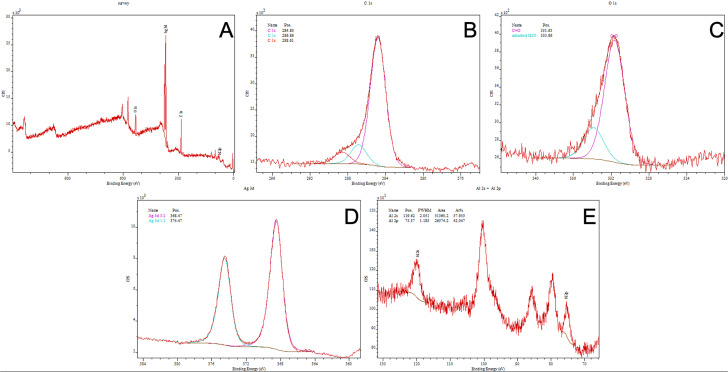
XPS characterization of the Al_2_O_3_ + Ag sample: (A) survey spectrum; (B) Ag 3d high-resolution spectrum; (C) O 1s high-resolution spectrum; (D) C 1s high-resolution spectrum; and (E) Al 2s/2p region. The XPS analysis results for plates 2 and 3 are provided in the SI (Fig. S8 and S10).

XPS analysis of the sample surface indicated the presence of silver in the surface layer (Fig. 4 and Table 4). Peaks corresponding to O 1s, C 1s, Ag 3d, and Al 2p were observed in the survey spectrum. In the Ag 3d region, two peaks were recorded at binding energies of 368.47 eV (Ag 3d_5/2_) and 374.47 eV (Ag 3d_3/2_), and the doublet splitting of 6.0 eV is consistent with metallic silver (Ag^0^).^17^ Analysis of the O 1s region revealed two components at 531.65 eV and 533.86 eV, corresponding to oxygen-containing surface species and adsorbed surface species, respectively. The C 1s spectrum exhibited three typical components: C–C/C=C (284.8 eV), C–O/C–OH (286.9 eV), and C=O (288.6 eV), originating from organic contamination layers and adsorbed carbon compounds. A weak Al 2p signal at 75.37 eV shows that the underlying Al_2_O_3_ layer remained detectable after Ag deposition (Table 5).

**Table 4:** Surface elemental composition of the Al_2_O_3_ + Ag sample determined by XPS survey analysis

% C	% O	% Ag	% Al
61.41	18.96	16.00	3.63

**Table 5:** Peak positions, assignments, and surface concentrations of the components identified in the high-resolution XPS spectra of the Al_2_O_3_ + Ag sample

Region	Binding energy (eV)	Assignment	Surface concentration (%)
C 1s	284.80	C–C/C=C	49.43
C 1s	286.86	C–O/C–OH	7.44
C 1s	288.61	C=O	4.54
O 1s	531.65	C=O/oxygen-containing surface species	15.11
O 1s	533.86	Adsorbed H_2_O/adsorbed surface species	3.85
Ag 3d	368.47/374.47	Ag 3d_5/2_ and Ag 3d_3/2_, metallic Ag (Ag^0^)	16.00
Al 2p	75.37	Al 2p Al_2_O_3_	3.63

**Fig. 5:**
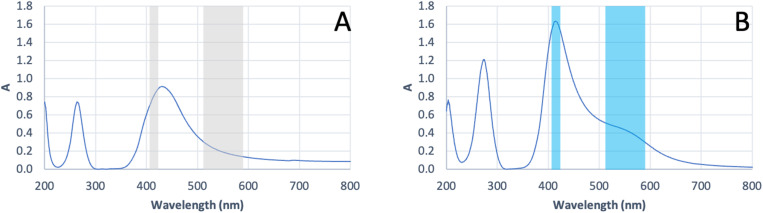
UV-Vis DRS spectra of samples (A) Al_2_O_3_ and (B) Al_2_O_3_ + AgNPs for plate 1. The corresponding UV-Vis DRS results for plates 2 and 3 are provided in the SI (Fig. S11, S12 and Tables S10, S11).

The UV-Vis DRS spectra of the Al_2_O_3_ and Al_2_O_3_ + Ag samples are presented in Fig. 5. The calculated band gap values (*E*_g_) and corresponding wavelengths (*λ*_max_) are summarized in Table 6. Three band gap values and their corresponding absorption maxima were determined for each sample using Tauc plots assuming direct and indirect allowed transitions. In the spectrum of the Al_2_O_3_ + Ag sample, a distinct absorption maximum is observed around 420 nm (marked in blue), which is absent in the pure Al_2_O_3_ layer. Additionally, a broadened band is visible in the 500–600 nm range (marked in blue), which was not observed for the Al_2_O_3_ sample.

**Table 6:** Calculated band gap values (*E*_g_) and corresponding wavelengths (*λ*_max_) for the Al_2_O_3_ and Al_2_O_3_ + AgNPs samples for plate 1. The corresponding values for plates 2 and 3 are provided in the SI (Tables S10 and S11)

Sample	Band gap (eV)	*λ*_max_ (nm)
Al_2_O_3_	2.50	200.5
	4.42	264.5
	5.96	431.0
Al_2_O_3_ + AgNPs	1.82	207.0
	2.66	279.5
	4.16	419.5

### 3.2 Qualitative analysis of selected standards in positive ion mode

**Fig. 6:**
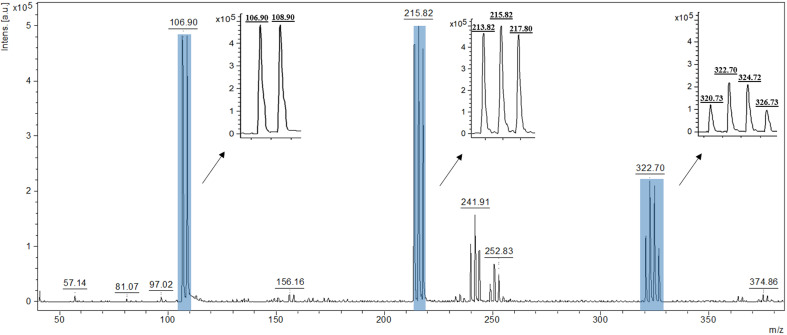
Spectrum of the Al_2_O_3_ + Ag substrate with marked characteristic Ag clusters.

**Fig. 7:**
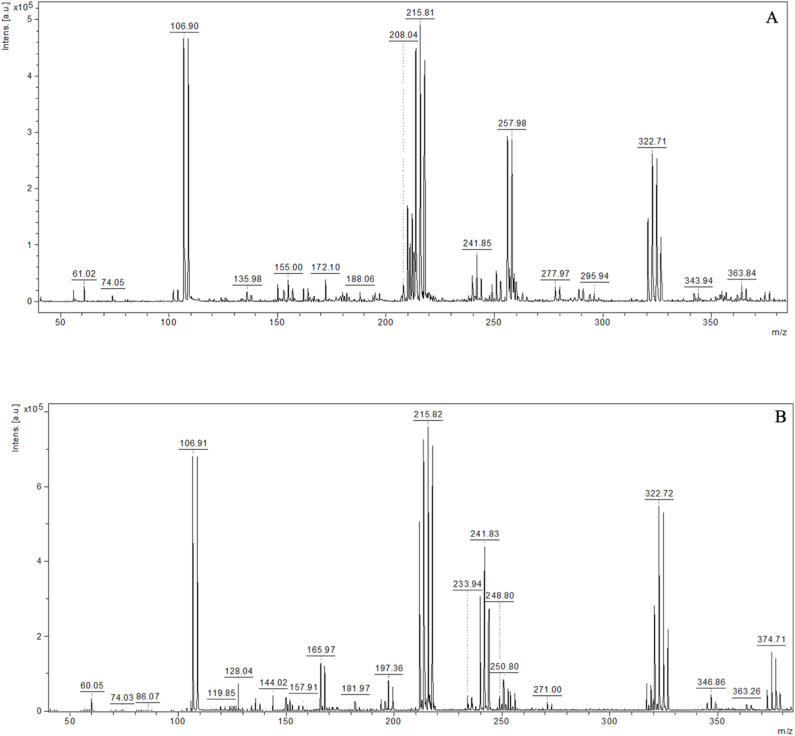
Representative spectra: (A) methionine, 200 µg mL; (B) serine, 200 µg mL^−1^.

**Fig. 8:**
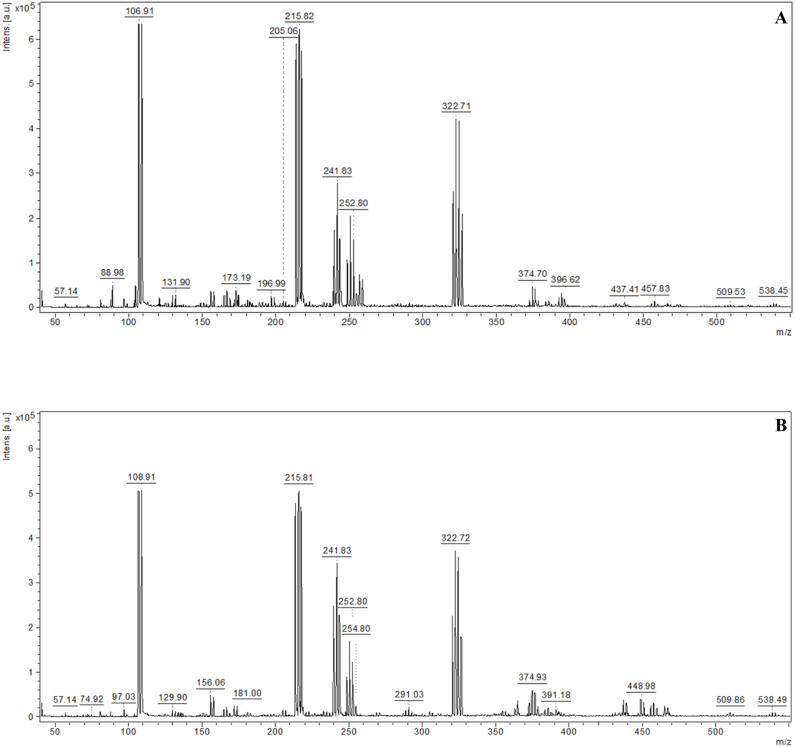
Representative spectra: (A) arabinose, 100 µg mL^−1^, (B) lactose, 100 µg mL^−1^.

To evaluate the analytical potential of the prepared substrates, measurements were performed by recording mass spectra of selected standard compounds. Low molecular weight compounds belonging to two chemical classes were used as standards: amino acids (serine, methionine) and saccharides (lactose and arabinose). Fig. 6 presents the spectrum obtained from the analysis of the plate surface. Fig. 7 and 8 present the mass spectra obtained for the highest analyte concentrations in the NALDI analysis. In the MS spectra of the analyzed standards, signals characteristic of protonated adducts ([M + H]^+^) and metal ion adducts ([M + K]^+^, [M + Na]^+^, [M + Ag]^+^) were observed. Additionally, signals originating from molecular fragments, including their respective adducts, as well as clusters, were also visible. Tables 7–10 present the proposed interpretation of signals obtained for the analyzed compounds at various concentrations, constituting a tentative assignment of possible ions. Signal reproducibility was assessed based on three measurements, and the number of occurrences of a given signal was indicated in the tables using superscripts. Average intensity and S/N values were calculated based on the replicates in which the given signal was recorded.

**Table 7:** Proposed interpretation of methionine (C_5_H_11_NO_2_S) signals in NALDI TOF MS spectra. Assigned ions are listed along with theoretical and observed *m*/*z* values, as well as mean intensity and S/N values for specific concentrations. Superscripts indicate the number of replicates in which the signal was observed. The table presents signals for which S/N ≥ 7

	Adduct/fragment	Molecular formula	*m*/*z*		Concentration [µg mL^−1^]					
			Theor.	Obs.	200	100	40	20	8	4
Methionine C_5_H_11_NO_2_S	[Fragment]^+^	C_2_H_5_S^+^	61.01	61.02	25 730 (40)^3^	19 757 (29)^3^	18 674 (17)^3^	8000 (14)^3^	5289 (12)^3^	—
	[Fragment]^+^	C_4_H_8_NO_2_^+^	102.06	102.14	24 944 (25)^3^	21 820 (21)^3^	18 614 (12)^3^	8951 (10)^3^	4821 (7)^2^	—
	[Fragment]^+^	C_4_H_10_NO_2_^+^	104.07	104.13	22 255 (21)^3^	20 840 (19)^3^	23 104 (14)^3^	10 592 (11)^3^	3677 (8)^1^	19 898 (37)^3^
	[M + H]^+^	C_5_H_12_NO_2_S^+^	150.06	150.13	24 042 (12)^3^	17 789 (9)^3^	—	—	—	—
	[M + Na]^+^	C_5_H_11_NO_2_SNa^+^	172.04	172.10	34 709 (16)^3^	31 264 (14)^3^	64 745 (15)^2^	11 949 (8)^3^		33 894 (29)^2^
	[M + K]^+^	C_5_H_11_NO_2_SK^+^	188.01	188.06	16 549 (8)^1^	18 670 (8)^1^	67 578 (14)^1^	—	—	—
	[Fragment + ^107^Ag]^+^	C_4_H_9_NO_2_^107^Ag	209.97	210.03	178 360 (67)^3^	161 957 (67)^3^	188 484 (54)^3^	65 155 (41)^3^	40 689 (30)^3^	31 591 (21)^3^
	[Fragment + ^109^Ag]^+^	C_4_H_9_NO_2_^109^Ag	211.97	212.04	158 336 (60)^3^	141 082 (58)^3^	148 825 (44)^3^	58 036 (36)^3^	35 668 (26)^3^	27 135 (18)^3^
	[Fragment + ^107^Ag]^+^	C_4_H_10_NO_2_^107^Ag^+^	210.98	211.04	115 624 (43)^3^	99 440 (41)^3^	82 703 (24)^3^	28 011 (17)^3^	15 322 (11)^3^	—
	[Fragment + ^109^Ag]^+^	C_4_H_10_NO_2_^109^Ag^+^	212.98	213.06	99 199 (37)^3^	84 957 (35)^3^	67 599 (20)^3^	23 153 (14)^3^	12 924 (10)^3^	—
	[M + ^107^Ag]^+^	C_5_H_11_NO_2_S^107^Ag^+^	255.96	255.98	309 259 (106)^3^	256 710 (97)^3^	200 400 (52)^3^	60 170 (34)^3^	31 425 (15)^3^	—
	[M + ^109^Ag]^+^	C_5_H_11_NO_2_S^109^Ag^+^	257.96	257.98	301 253 (103)^3^	249 351 (95)^3^	200 592 (51)^3^	61 526 (36)^3^	31 996 (15)^3^	—
	[M–H + Na + ^107^Ag]^+^	C_5_H_10_NO_2_SNa^107^Ag^+^	277.94	277.97	24 363 (9)^3^	21 682 (9)^3^	62 979 (12)^1^	—	—	—
	[M–H + Na + ^109^Ag]^+^	C_5_H_10_NO_2_SNa^109^Ag^+^	279.94	279.97	24 075 (9)^3^	21 673 (9)^3^	62 662 (12)^1^	—	—	—

**Table 8:** Proposed interpretation of serine (C_3_H_7_NO_3_) signals in NALDI TOF MS spectra. Assigned ions are listed along with theoretical and observed *m*/*z* values, as well as mean intensity and S/N values for specific concentrations. Superscripts indicate the number of replicates in which the signal was observed. The table presents signals for which S/N ≥ 7

	Adduct/fragment	Molecular formula	*m*/*z*		Concentration [µg mL^−1^]					
			Theor.	Obs.	200	100	40	20	8	4
Serine C_3_H_7_NO_3_	[Fragment]^+^	C_2_H_6_NO^+^	60.04	60.05	31 817 (60)^3^	32 463 (81)^3^	10 521 (39)^3^	4279 (14)^2^	—	—
	[M + H]^+^	C_3_H_8_NO_3_	106.05	106.06	23 413 (43)^3^	18 692 (30)^3^	4220 (9)^2^	—	—	
	[M + Na]^+^	C_3_H7NO_3_Na^+^	128.03	128.04	60 354 (128)^3^	48 965 (58)^3^	36 110 (62)^3^	9102 (15)^2^	—	—
	[M + K]^+^	C_3_H_7_NO_3_K^+^	144.01	144.02	33 779 (144)^3^	88 487 (92)^3^	20 646 (32)^3^	13 591 (13)^2^	—	—
	[Fragment + ^107^Ag]^+^	C_2_H_5_NOAg^+^	165.94	165.97	129 894 (166)^3^	144 083 (121)^3^	76 800 (98)^3^	56 900 (56)^3^	10 092 (11)^3^	9784 (12)^3^
	[Fragment + ^109^Ag]^+^	C_2_H_5_NOAg^+^	167.94	167.96	120 202 (168)^3^	133 038 (112)^3^	45 184 (59)^3^	28 925 (31)^3^	8054 (9)^3^	8347 (10)^3^
	[M + 2K−H]^+^	C_3_H_6_NO_3_K_2_^+^	181.96	181.97	20 961 (182)^3^	96 721 (78)^3^	25 115 (30)^3^	27 145 (24)^3^	8523 (8)^1^	—
	[2M + H−NH_3_]^+^	C_6_H_12_NO_6_^+^	194.07	193.99	29 718 (194)^3^	30 916 (23)^3^	13 538 (16)^3^	8721 (7)^2^	—	—
	[M + ^107^Ag]^+^	C_3_H_7_NO_3_Ag^+^	211.95	211.96	483 591 (212)^3^	477 358 (327)^3^	187 661 (198)^3^	—	—	—
	[M + ^109^Ag]^+^	C_3_H_7_NO_3_Ag^+^	213.95	213.96	527 972 (412)^3^	528 563 (362)^3^	296 655 (287)	—	—	—
	[M + ^107^Ag + Na + H_3_O^+^]^+^	C_5_H_9_NO_4_Ag^+^	253.96	253.97	49 317 (254)^3^	47 758 (30)^3^	13 242 (12)^3^	—	—	—
	[M + ^109^Ag + Na + H_3_O^+^]^+^	C_5_H_9_NO_4_Ag^+^	255.96	255.97	47 949 (256)^3^	47 307 (29)^3^	13 779 (12)^3^	—	—	—

**Table 9:** Proposed interpretation of arabinose (C_5_H_10_O_5_) signals in NALDI TOF MS spectra. Assigned ions are listed along with theoretical and observed *m*/*z* values, as well as mean intensity and S/N values for specific concentrations. Superscripts indicate the number of replicates in which the signal was observed. The table presents signals for which S/N ≥ 7

	Adduct/fragment	Molecular formula	*m*/*z*	Concentration [µg mL^−1^]						
			Theor.	Obs.	100	50	10	5	1	0.05
Arabinose C_5_H_10_O_5_	[Fragment]^+^	C_3_H_4_O_2_^+^	72.02	72.07	6502 (10)^3^	6113 (10)^3^	16 541 (26)^3^	50 120 (88)^3^	50 416 (109)^2^	34 516 (54)^3^
	[Fragment]^+^	C_3_H_5_O_3_^+^	89.02	88.98	46 094 (49)^3^	17 472 (21)^3^	39 765 (47)^3^	52 035 (73)^3^	30 178 (52)^2^	51 347 (68)^3^
	[Fragment]^+^	C_4_H_9_O_3_^+^	105.06	104.96	48 505 (44)^3^	46 700 (52)^3^	57 789 (61)^3^	44 896 (55)^3^	20 380 (32)^2^	42 136 (50)^3^
	[Fragment]^+^	C_4_H_9_O_4_^+^	121.05	120.95	22 245 (16)^3^	46 357 (44)^3^	35 381 (31)^3^	13 636 (14)^3^	—	13 515 (13)^3^
	[M + Na]^+^	C_5_H_10_O_5_Na^+^	173.04	173.19	29 957 (18)^3^	16 890 (14)^3^	22 891 (18)^3^	13 435 (11)^3^	11 096 (10)^2^	21 723 (17)^3^
	[Fragment + ^107^Ag]^+^	C_3_H_6_O_3_Ag^+^	196.94	196.99	22 014 (12)^3^	17 176 (12)^2^	—	—	—	—
	[Fragment + ^109^Ag]^+^	C_3_H_6_O_3_Ag^+^	198.94	198.99	20 222 (11)^3^	11 372 (8)^2^	—	—	—	—
	[M + ^107^Ag]^+^	C_5_H_10_O_5_Ag^+^	256.96	257.01	62 688 (35)^3^	25 358 (19)^3^	—	—	—	—
	[M + ^109^Ag]^+^	C_5_H_10_O_5_Ag^+^	258.96	258.99	52 635 (29)^3^	20 094 (15)^3^	—	—	—	—

**Table 10:** Proposed interpretation of lactose (C_12_H_22_O_11_) signals in NALDI TOF MS spectra. Assigned ions are listed along with theoretical and observed *m*/*z* values, as well as mean intensity and S/N values for specific concentrations. Superscripts indicate the number of replicates in which the signal was observed. The table presents signals for which S/N ≥ 7

	Adduct/fragment	Molecular formula	*m*/*z*		Concentration [µg mL^−1^]					
			Theor.	obs.	100	50	10	5	1	0.05
Lactose C_12_H_22_O_11_	[Fragment]^+^	C_4_H_5_O^+^	69.03	69.13	3949 (8)^1^	3422 (7)^2^	—	—	—	—
	[Fragment]^+^	C_3_H_4_O_2_^+^	72.02	72.07	7131 (14)^1^	9204 (20)^2^	9711 (11)^3^	28 663 (46)^3^	74 524 (111)^2^	37 253 (55)^3^
	[Fragment]^+^	C_3_H_7_O_2_^+^	75.04	74.92	4170 (8)^1^	4437 (9)^3^	—	—	—	—
	[Fragment]^+^	C_4_H_8_O_2_^+^	88.05	88.03	13 232 (14)^3^	13 909 (26)^2^	54 354 (52)^3^	56 445 (72)^3^	73 417 (88)^2^	48 639 (51)^3^
	[Fragment]^+^	C_5_H_7_O_2_^+^	99.04	98.98	54 079 (38)^3^	8178 (13)^2^	76 544 (70)^3^	50 776 (64)^3^	44 347 (49)^2^	8023 (8)^1^
	[Fragment]^+^	C_4_H_8_O_3_^+^	104.05	104.12	17 106 (12)^3^	8713 (13)^2^	27 616 (24)^3^	22 906 (27)^3^	19 909 (21)^3^	29 395 (23)^2^
	[Fragment]^+^	C_5_H_5_O_3_^+^	113.02	112.92	108 491 (63)^3^	7540 (9)^2^	17 545 (13)^3^	16 120 (18)^3^	15 761 (14)^3^	—
	[Hexose + H]^+^	C_6_H_13_O_6_^+^	181.07	181.00	49 011 (25)^3^	10 581 (9)^2^	51 233 (34)^3^	27 850 (25)^3^	18 039 (14)^3^	13 031 (8)^1^
	[Fragment + ^107^Ag]^+^	C_5_H_6_O_2_Ag^+^	204.94	205.02	15 138 (10)^2^	16 330 (12)^2^	—	33 456 (26)^1^	24 414 (18)^3^	25 716 (16)^3^
	[Fragment + ^109^Ag]^+^	C_5_H_6_O_2_Ag^+^	206.94	207.02	13 478 (8)^2^	15 143 (11)^2^	—	32 499 (25)^1^	24 264 (18)^3^	25 183 (16)^3^
	[Hexose + K + H_3_O]^+^	C_6_H_15_O_7_K^+^	238.05	238.87	20 090 (10)^2^	—	21 870 (14)^3^	16 132 (16)^2^	12 515 (9)^3^	—
	[M + Na]^+^	C_12_H_22_O_11_Na^+^	365.11	365.18	36 688 (25)^3^	33 953 (24)^3^	24 287 (18)^3^	9231 (10)^2^	—	—
	[M + ^107^Ag]^+^	C_12_H_22_O_11_Ag^+^	449.02	448.98	37 575 (36)^3^	16 394 (13)^3^	—	—	—	—
	[M + ^109^Ag]^+^	C_12_H_22_O_11_Ag^+^	451.02	450.98	29 282 (27)^3^	13 078 (10)^3^	—	—	—	—
	[M + O + ^107^Ag]^+^	C_12_H_22_O_12_Ag^+^	465.02	465.23	21 026 (20)^3^	26 495 (23)^3^	16 056 (14)^3^	10 319 (13)^2^	—	—
	[M + O + ^109^Ag]^+^	C_12_H_22_O_12_Ag^+^	467.02	467.24	19 032 (18)^3^	23 852 (21)^3^	12 908 (11)^3^	8359 (10)^2^	—	—

In the mass spectrum recorded for the substrate itself (Fig. 6), characteristic, intense signals originating from silver nanoparticles are observed as clusters of ^107^Ag and ^109^Ag isotopes. Among the most characteristic signals, envelopes located at *m*/*z* 106.90 and 108.90; 213.82, 215.82, and 217.80; as well as 320.73, 322.70, 324.72, and 326.73 are visible. A detailed description of the substrate signals is included in the SI, which is essential for unambiguous assignment of analyte-derived ions in subsequent NALDI experiments (SI Table S1).

The recorded spectra of methionine and serine exhibited the presence of signals corresponding to the protonated ion [M + H]^+^ and adducts with metal ions [M + Na]^+^, [M + K]^+^ and [M + ^107/109^Ag]^+^. Signals with *m*/*z* values lower than the molecular mass of the analyte were also observed, indicating the presence of fragment ions, as well as signals suggesting the formation of adducts with molecular fragments. In both cases, the highest intensity was obtained for silver adducts. Signals were most abundant in the higher concentration range (200–40 µg mL^−1^), whereas their number decreased with decreasing concentration. Methionine fragments comprising ions in the *m*/*z* range of 61–104 were consistently recorded down to a concentration of 8 µg mL^−1^. In contrast, serine fragments in the *m*/*z* range of 60–144 were consistently recorded down to a concentration of 20 µg mL^−1^. Based on triplicate measurements, the relative standard deviation (RSD) of the [M + ^107^Ag]^+^ signal intensity for methionine at 8 µg mL^−1^ was 51%, and the corresponding limit of detection (LOD, S/N = 3) was estimated at 1.68 ± 0.33 µg mL^−1^. A list of recorded and identified ions is presented in Tables 7 and 8.

The recorded spectra of lactose and arabinose were characterized by the presence of numerous fragmentation signals with *m*/*z* values lower than the molecular mass of the investigated compounds. In the case of lactose, fragments in the 69–181 *m*/*z* range dominated, whereas for arabinose, they appeared in the 72–121 *m*/*z* range. Besides fragments, signals corresponding to sodium adducts ([M + Na]^+^) and adducts with silver ions, including [M + ^107/109^Ag]^+^, were also recorded, observed mainly at higher concentrations (100–10 µg mL^−1^). Adducts [M + ^107/109^Ag]^+^ were detected up to a concentration of 50 µg mL^−1^ (S/N ≥ 7), while at lower concentrations the spectra were dominated by fragment ion signals. Most fragmentation signals were recorded across the entire concentration range (100–0.05 µg mL^−1^). A list of recorded and identified ions is presented in Tables 9 and 10.

## 4 Discussion

In this study, we combined ALD and CVD to deposit Al_2_O_3_ nanolayers on steel and subsequently form AgNPs coatings. Three substrates were prepared to evaluate process reproducibility. The performance of the resulting NALDI plates was assessed using amino acid and saccharide standards, focusing on the occurrence of ion signals across a range of analyte concentrations. SEM images confirmed the presence of AgNP grains distributed over the Al_2_O_3_ surface, even though the Al_2_O_3_ layer itself did not exhibit perfectly homogeneous topography (Fig. 1 and SI S2 and S3). Nonetheless, noticeable differences in grain size were observed among the three plates. These variations are most likely related to the individual topography and roughness of each steel substrate, which may be partially replicated in the deposited layers and subsequently influence nanoparticle size and distribution. In this context, local depressions, protrusions, and other surface irregularities may promote non-uniform nucleation and growth, leading to heterogeneous morphology.^18^ AFM analysis showed that AgNPs deposition significantly modified the substrate topography; however, the magnitude and direction of these changes depended on the initial roughness of the plate. The differing trends in *R*_a_ and *R*_q_ for the three plates indicate that AgNPs did not form an identical surface layer on all substrates, but instead responded sensitively to local surface properties. From an LDI perspective, such differences in roughness and AgNPs morphology are crucial, as they may modulate local laser energy absorption and, consequently, analyte desorption/ionization efficiency. Literature reports underscore that nanostructure size and density critically determine both sensitivity and spectral features in low-molecular-weight LDI analysis. Sagandykova *et al.* demonstrated that the amount of precursor used in the CVD process directly affects substrate morphology and the resulting LDI response for particular analyte classes.^9^ Similarly, Arendowski *et al.* showed that electrochemical parameters (salt type, voltage, deposition time) influence the formation of “flower-like” micro- and nanostructures and, in turn, the sensitivity and background level in SALDI MS.^19^ Müller *et al.* emphasized that nanosubstrate properties such as topography, morphology, and roughness are among the key determinants of desorption/ionization efficiency and data reproducibility in SALDI MS.^16^ Consequently, various substrate preparation strategies have been explored to control surface parameters; for example, Naito *et al.* employed porous anodic aluminum oxide membranes with well-defined pore geometry, which improved analyte trapping and ionization reproducibility.^20^ In our work, aluminum oxide was selected as an intermediate layer because of its chemical stability and because it can provide a suitable platform for silver nanoparticle deposition. However, the results obtained here indicate that, in the case of steel-supported samples, the final surface morphology must be interpreted in the context of the initial substrate topography. This interpretation is additionally supported by the supplementary SEM and AFM data for the unmodified steel substrate, which revealed a heterogeneous and directionally developed topography with local irregularities (SI Fig. S13, S14 and Table S12), whereas the reference Al_2_O_3_ layer deposited on Si exhibited a markedly more uniform surface morphology and substantially lower roughness (SI Fig. S15, S16 and Table S13). Despite this, the reproducibility of AgNPs morphology was not ideal, which likely reflects the combined effect of substrate-related variability and potential limitations of the CVD process, such as local variations in nucleation density, growth kinetics, or precursor delivery. EDX (Fig. 2 and SI S4 and S5) and XPS (Fig. 3 and 4, SI S7–S10) confirmed the presence of silver after the CVD step. In particular, the Ag 3d doublet observed in the XPS spectra is consistent with metallic silver (Ag^0^), indicating that the deposited nanostructures retained the chemical state relevant to plasmon-assisted LDI processes. In the EDX spectra of the steel-supported samples, signals originating from Fe, Cr, and Ni were also observed, which can be attributed to the contribution of the underlying substrate under the applied measurement conditions. Therefore, the EDX results should be treated primarily as confirmation of the presence of Ag together with Al/O-containing surface components. This interpretation is additionally supported by the EDX results obtained for the reference Al_2_O_3_ layer deposited on Si, for which only Si, O, and Al were detected (SI Fig. S17 and S18), consistent with the expected composition of the oxide-coated reference substrate. The weak Al 2p signal still observed after Ag deposition indicates that the Al_2_O_3_ support remained detectable within the XPS sampling depth, which is consistent with nanoparticle-type surface coverage rather than a fully continuous metallic overlayer. For the Al_2_O_3_-coated samples (Fig. 3, SI S7, S9 and Table 3; SI S3 and S7), the higher-binding-energy Al 2p contribution assigned to Al–OH is consistent with surface hydroxylation; notably, the reference Al_2_O_3_ layer deposited on Si also showed a second Al 2p component at 75.3 eV together with a higher-binding-energy O 1s contribution at approximately 532.4 eV (SI Fig. S19 and Table S15), supporting the presence of hydroxylated surface species on alumina.^21^ UV-Vis DRS data (Fig. 5 and Table 6; SI Fig. S11 and S12; Tables S10 and S11) further show that AgNPs deposition altered the optical response of the system. The appearance of a distinct band at approximately 420 nm is consistent with localized surface plasmon resonance (LSPR) of silver nanoparticles and thus supports the presence of plasmonically active Ag structures on the surface.^22,23^ In addition, the broad absorption extending into the 500–600 nm region may be associated with a broader particle size distribution, local aggregation, or morphological heterogeneity of the Ag nanostructures, which is consistent with the SEM observations for the analyzed plates. Taken together, the EDX, XPS, and UV-Vis DRS results indicate that the obtained substrates contain metallic silver nanostructures with optical properties relevant to laser energy absorption, while the surface remains structurally heterogeneous rather than ideally uniform. For stoichiometric Al_2_O_3_, the expected O/Al atomic ratio is 1.5.^24^ In the present study, the O/Al ratios derived from the XPS survey spectra were approximately 1.32 for the Al_2_O_3_ sample discussed in the main text (Fig. 3 and Table 2), 1.39 for plate 2 (SI Fig. S7 and Table S2), 1.39 for plate 3 (SI Fig. S9 and Table S6), and 1.37 for the reference Al_2_O_3_ layer deposited on Si (SI Fig. S19 and Table S14), while the corresponding carbon contents were 29.29, 32.56, 27.49, and 17.77 at%, respectively. Slightly oxygen-deficient Al_2_O_3_ films grown by ALD at 200 °C have also been reported in the literature.^25^ At the same time, XPS probes only the outermost surface region, typically within about 5–10 nm, and the measured atomic ratios can be strongly influenced by adsorbed species and adventitious carbon present at the surface.^26^ In this context, the relatively high carbon contribution observed here most likely reflects surface contamination and adsorption effects and may contribute to the deviation of the survey-derived O/Al values from the ideal stoichiometric ratio.^26^ Therefore, the XPS-derived O/Al ratios are best interpreted as surface-sensitive compositions of the outermost layer rather than as a direct measure of the bulk stoichiometry of the deposited Al_2_O_3_ film.^26^ The analytical performance of the Al_2_O_3_/Ag substrates was probed using amino acids (methionine, serine) and saccharides (arabinose, lactose) over a wide concentration range in positive-ion mode, which generally offers higher sensitivity.^27^ For all analytes, adducts with metal ions, including Na^+^, K^+^, and Ag^+^, were detected (Tables 7–10). The spectra also contained numerous fragment ions, fragment-ion adducts, and species interpreted as clusters. Saccharides (both lactose and arabinose) exhibited pronounced fragmentation, consistent with the lability of C–O bonds in these molecules. In contrast, amino acids displayed a higher propensity to form metal-ion adducts, particularly with silver, indicating more favorable interactions between these molecules and the AgNP-coated surface. The faster disappearance of serine signals compared to methionine suggests that differences in affinity for the silver surface are related to molecular composition and the presence of specific functional groups. This observation aligns with reports that Ag^+^ ions exhibit high affinity toward sulfur-containing compounds.^28^ At the same time, amino acids showed less extensive fragmentation than saccharides, suggesting a milder ionization pathway under the applied conditions. Overall, the obtained results indicate that the Al_2_O_3_/Ag substrates are suitable for qualitative NALDI analysis of amino acids and saccharides. However, it must be emphasized that signal detectability and reproducibility at lower concentrations are not yet fully satisfactory. In the context of the structural heterogeneity discussed above, this behavior may reflect local differences in nanoparticle distribution, roughness, and surface activity, which can translate into spatial variation of ionization efficiency across the substrate. In our previous work on silver-based substrates, the formation of metallic adducts enabled calibration curves over a much broader dynamic range than that achieved here.^7^ It is therefore conceivable that the Al_2_O_3_ underlayer modifies the surface properties of the overlying silver and indirectly affects the ionization process, for example, by altering the local work function, surface charge distribution, or accessibility of high-field “hot spots”, which in turn may influence the balance between desorption efficiency and gas-phase ionization.^7,9^ Changes in substrate character could contribute to reduced signal intensity and a narrower range of detectable concentrations. It should also be noted that interpreting NALDI spectra recorded on metal-containing substrates remains challenging because there are no dedicated reference spectral databases for such materials. Metal-containing nanostructured substrates produce characteristic signals that differ from those observed in conventional MALDI, both in fragmentation patterns and in the formation of specific adducts.^29^ Moreover, metal-nanostructure-based techniques often promote extensive fragmentation, resulting in complex low-*m*/*z* patterns that are difficult to assign unambiguously. The absence of suitable databases and the diversity of surface processes mean that NALDI data analysis still relies largely on manual interpretation and is associated with increased uncertainty. Systematic compilation of NALDI spectra for well-defined nanoparticle substrates and model analytes would therefore be highly valuable for the field, and the detailed signal assignments presented here in Tables 7–10 provide a useful starting point in this direction.

## 5 Conclusions

This study demonstrates that ALD-grown Al_2_O_3_ interlayers combined with CVD-grown AgNPs can be used to fabricate matrix-free LDI substrates on stainless steel. The resulting surfaces contain metallic silver and exhibit optical features consistent with plasmonically active Ag nanostructures, while the comparative characterization further shows that the morphology of the final Ag layer is strongly conditioned by the topography of the underlying substrate. In analytical tests, the Al_2_O_3_/Ag plates supported qualitative detection of amino acids and saccharides, with particularly prominent Ag-adduct formation and extensive saccharide fragmentation. At the same time, the variability in surface morphology was reflected in limited signal reproducibility and a relatively narrow concentration window for reliable signal assignment.

The main contribution of the present work is therefore not only the fabrication of ALD/CVD-derived Al_2_O_3_/Ag NALDI substrates, but also the demonstration that substrate topography is a critical design variable for further optimization of NALDI performance.

## Author contributions

Jagoda Pałczyńska: writing – original draft; conceptualization; methodology; investigation; data curation; formal analysis; visualization; validation; writing – review & editing. Ewelina Sibińska: methodology; formal analysis; writing – review & editing. Piotr Piszczek: methodology; conceptualization; resources; writing – review & editing. Kinga Robotnik: investigation; writing – review & editing. Aleksandra Radtke: supervision; writing – review & editing. Oleksandra Pryshchepa: supervision; writing – review & editing. Marek Trzcinski: formal analysis; visualization; writing – review & editing. Weronika Brzozowska: formal analysis; visualization; writing – review & editing. Dominika Przybysz: writing – review & editing. Paweł Pomastowski: conceptualization; resources; funding acquisition; project administration; supervision; writing – review & editing.

## Conflicts of interest

There are no conflicts to declare.

## Supplementary Material

RA-016-D5RA09570K-s001

RA-016-D5RA09570K-s002

RA-016-D5RA09570K-s003

RA-016-D5RA09570K-s004

RA-016-D5RA09570K-s005

RA-016-D5RA09570K-s006

RA-016-D5RA09570K-s007

RA-016-D5RA09570K-s008

RA-016-D5RA09570K-s009

RA-016-D5RA09570K-s010

RA-016-D5RA09570K-s011

RA-016-D5RA09570K-s012

RA-016-D5RA09570K-s013

RA-016-D5RA09570K-s014

RA-016-D5RA09570K-s015

RA-016-D5RA09570K-s016

RA-016-D5RA09570K-s017

RA-016-D5RA09570K-s018

RA-016-D5RA09570K-s019

RA-016-D5RA09570K-s020

RA-016-D5RA09570K-s021

## Data Availability

Processed MS data supporting this study are openly available in Zenodo (DOI: https://doi.org/10.5281/zenodo.17876562). Supplementary information (SI) is available. See DOI: https://doi.org/10.1039/d5ra09570k.
