# Supplementary figures and images for: Al2O3/Ag nanostructured substrates prepared by ALD/CVD for matrix-free laser desorption/ionization mass spectrometry
Source: RSC Adv. 2026 May 20;16(29):27035–47. doi: 10.1039/d5ra09570k (PMC13191748; doi:10.1039/d5ra09570k)

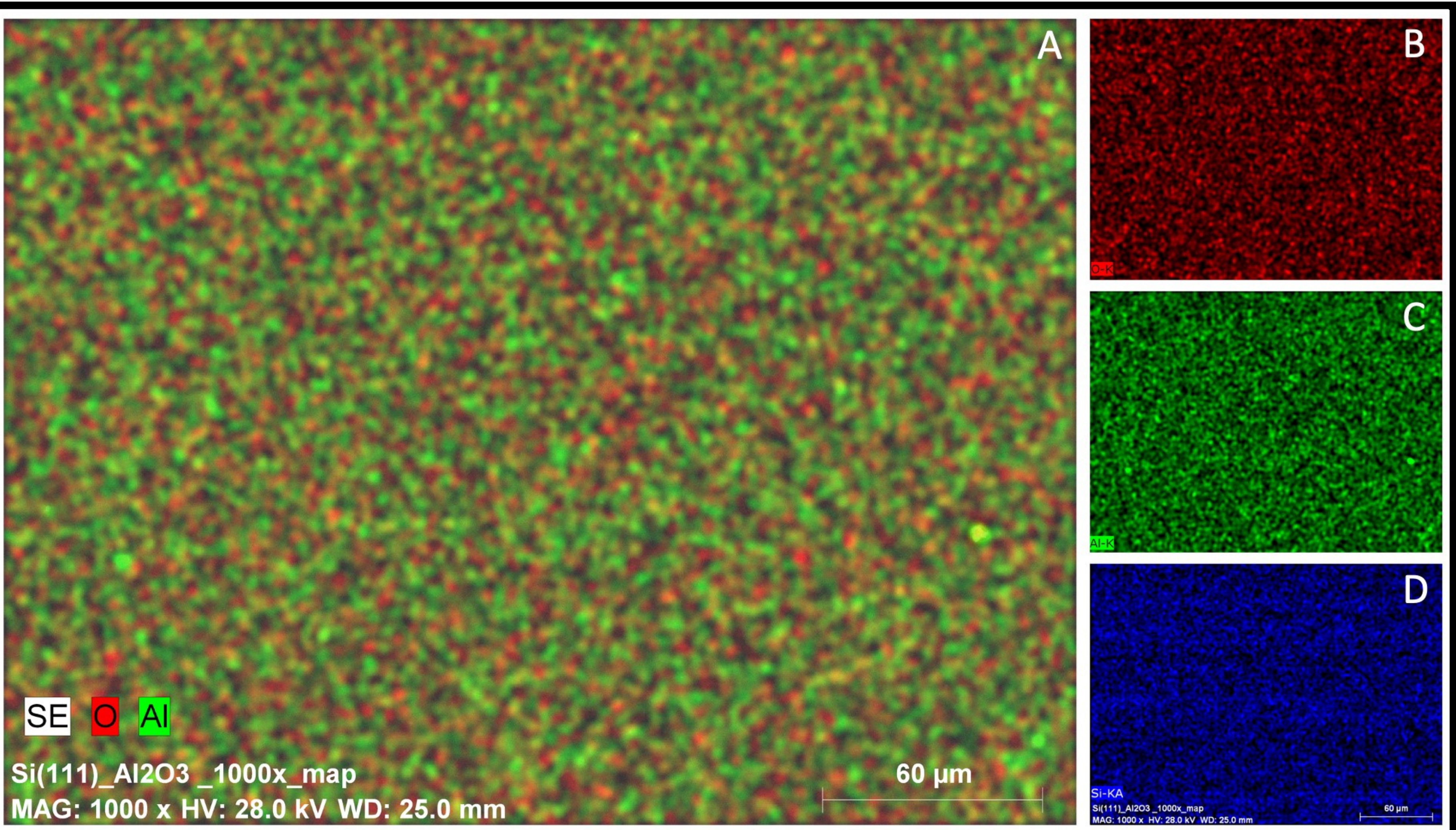

Supplement: RA-016-D5RA09570K-s002 [file RA-016-D5RA09570K-s002.pdf]

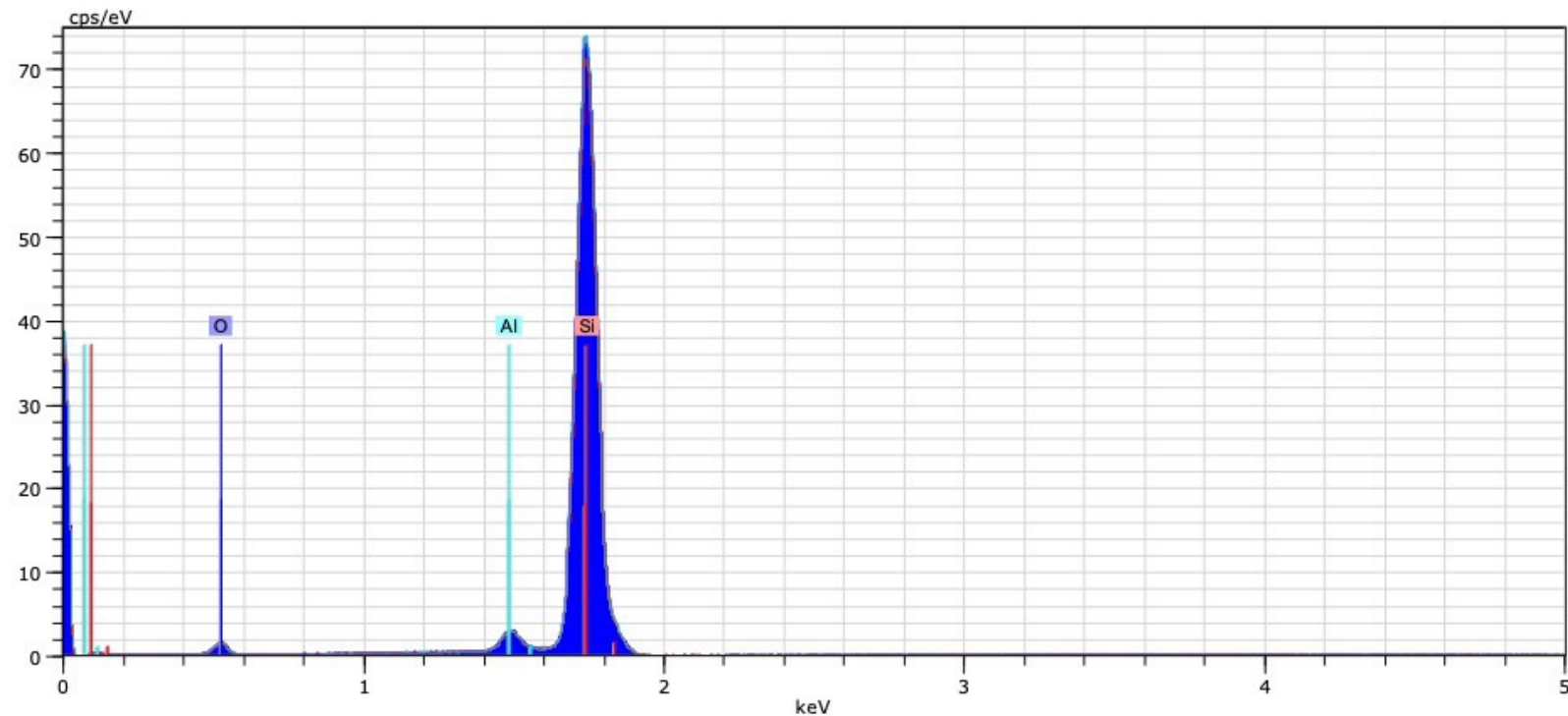

Atomic percent (%)

| Spectrum    | O     | Al   | Si    |
|-------------|-------|------|-------|
| 65201       | 26.71 | 2.35 | 70.94 |
| 65202       | 25.92 | 2.46 | 71.63 |
| 65203       | 26.25 | 2.39 | 71.36 |
| Mean value: | 26.29 | 2.40 | 71.31 |
| Sigma:      | 0.40  | 0.05 | 0.35  |
| Sigma mean: | 0.23  | 0.03 | 0.20  |

Supplement: RA-016-D5RA09570K-s003 [file RA-016-D5RA09570K-s003.pdf]

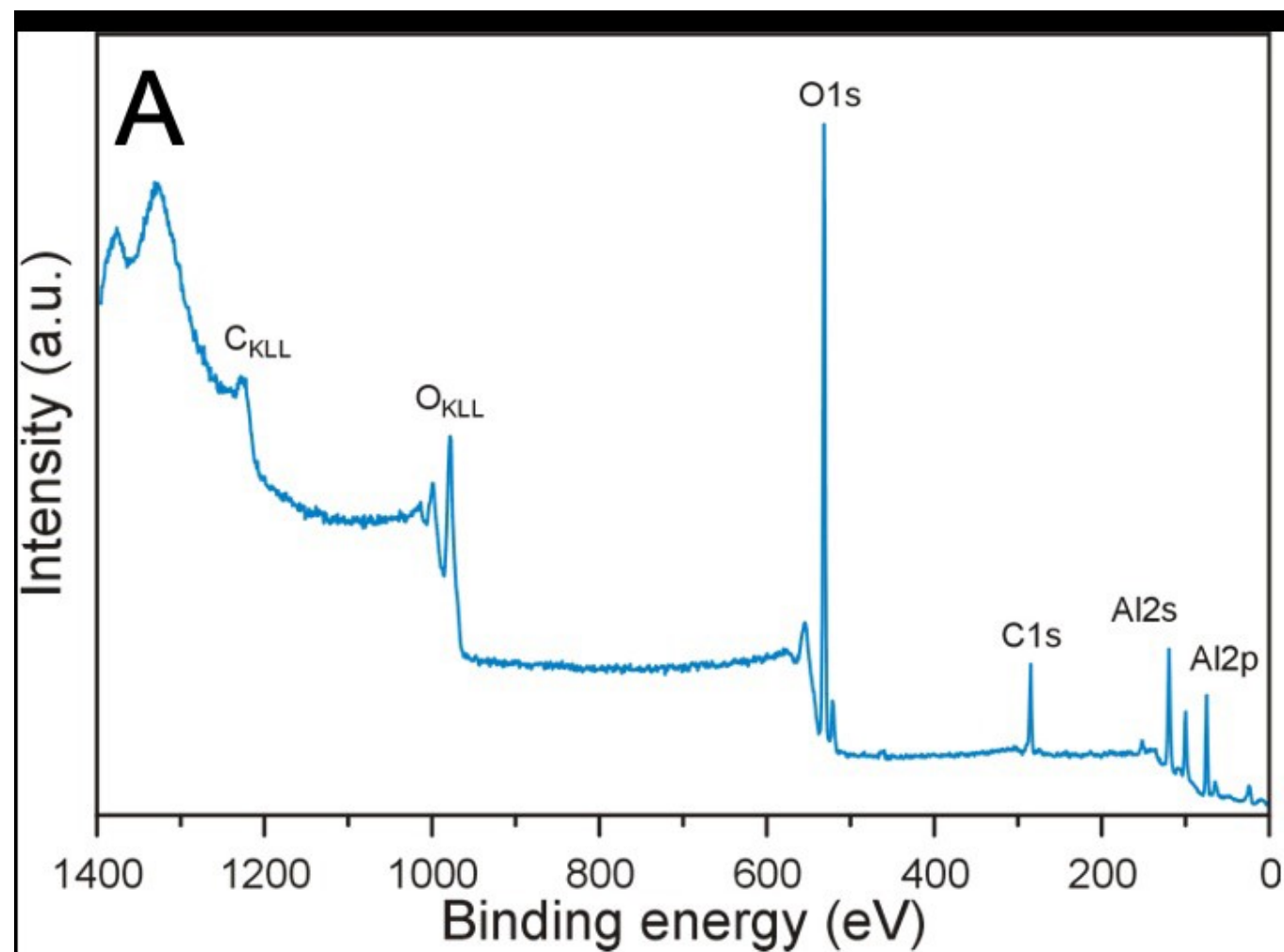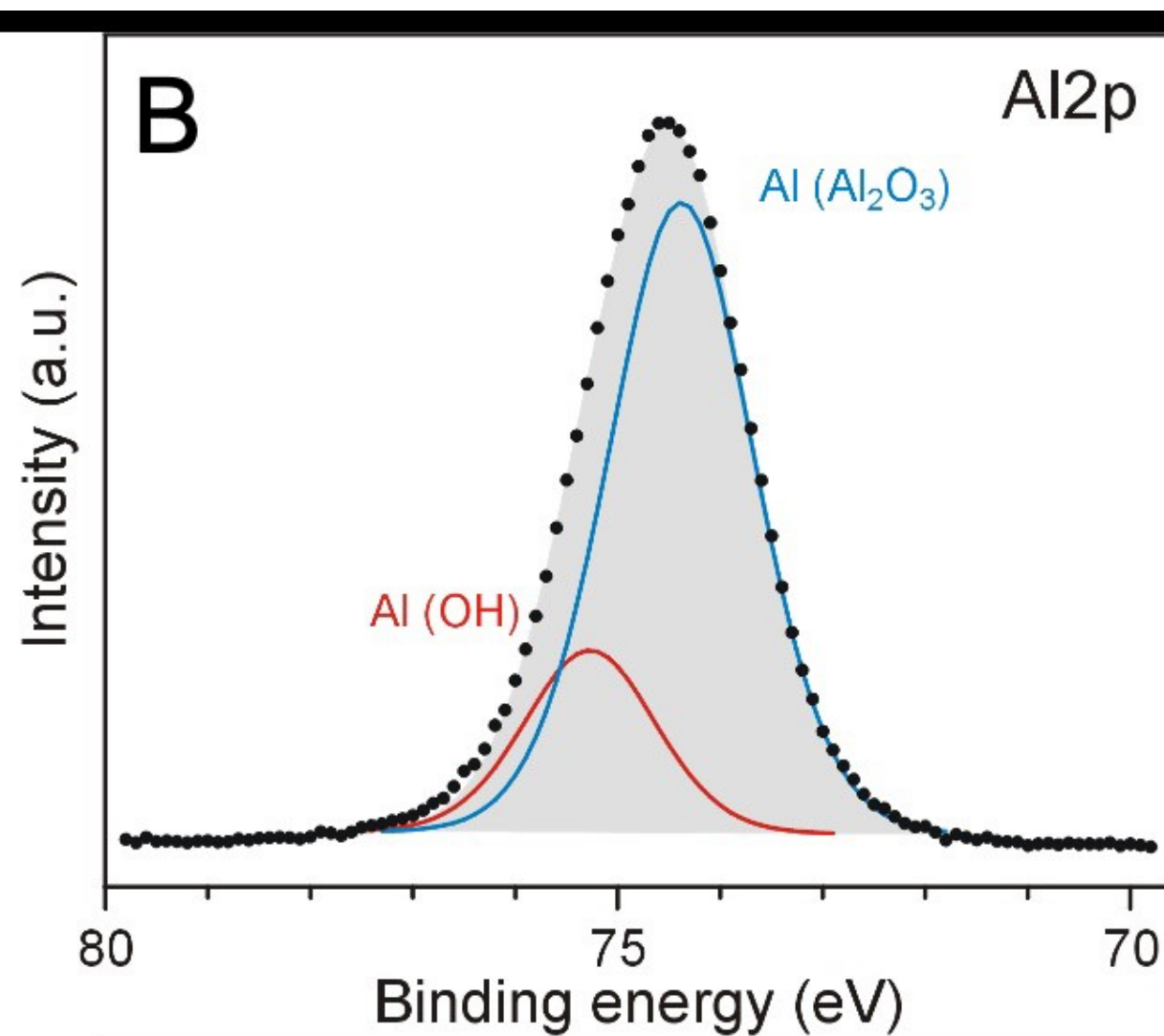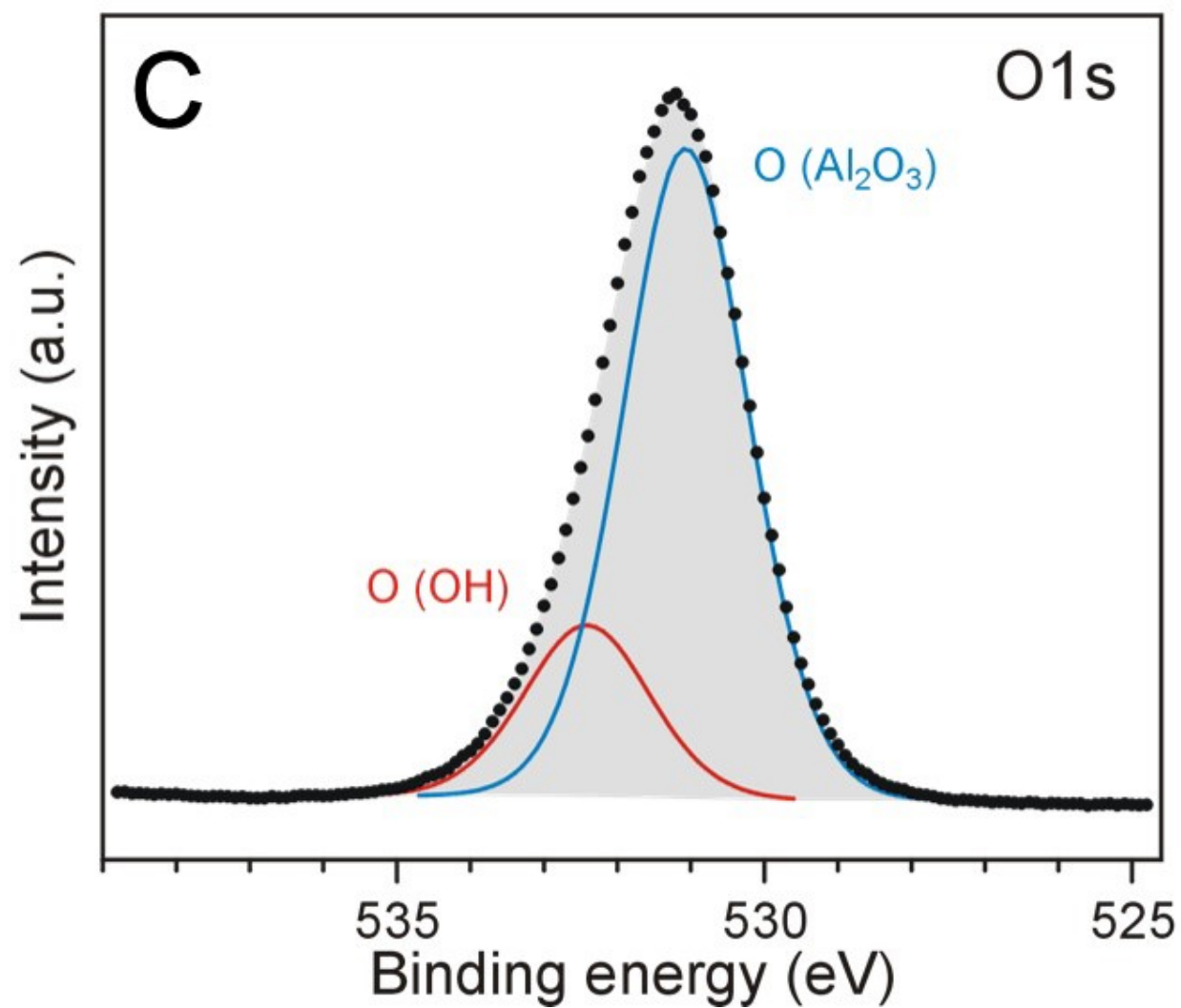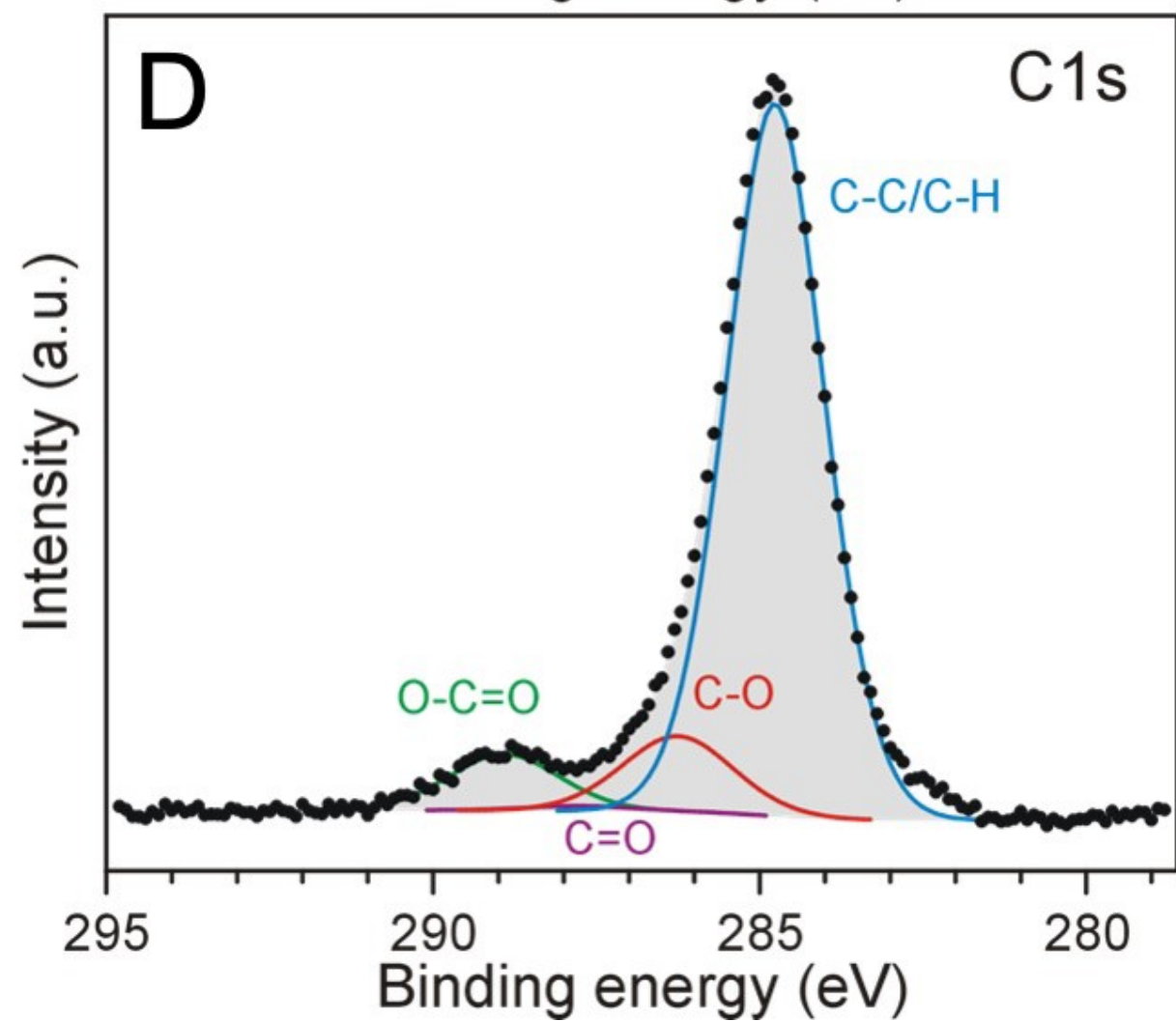

Supplement: RA-016-D5RA09570K-s004 [file RA-016-D5RA09570K-s004.pdf]

$\text{Al}_2\text{O}_3$

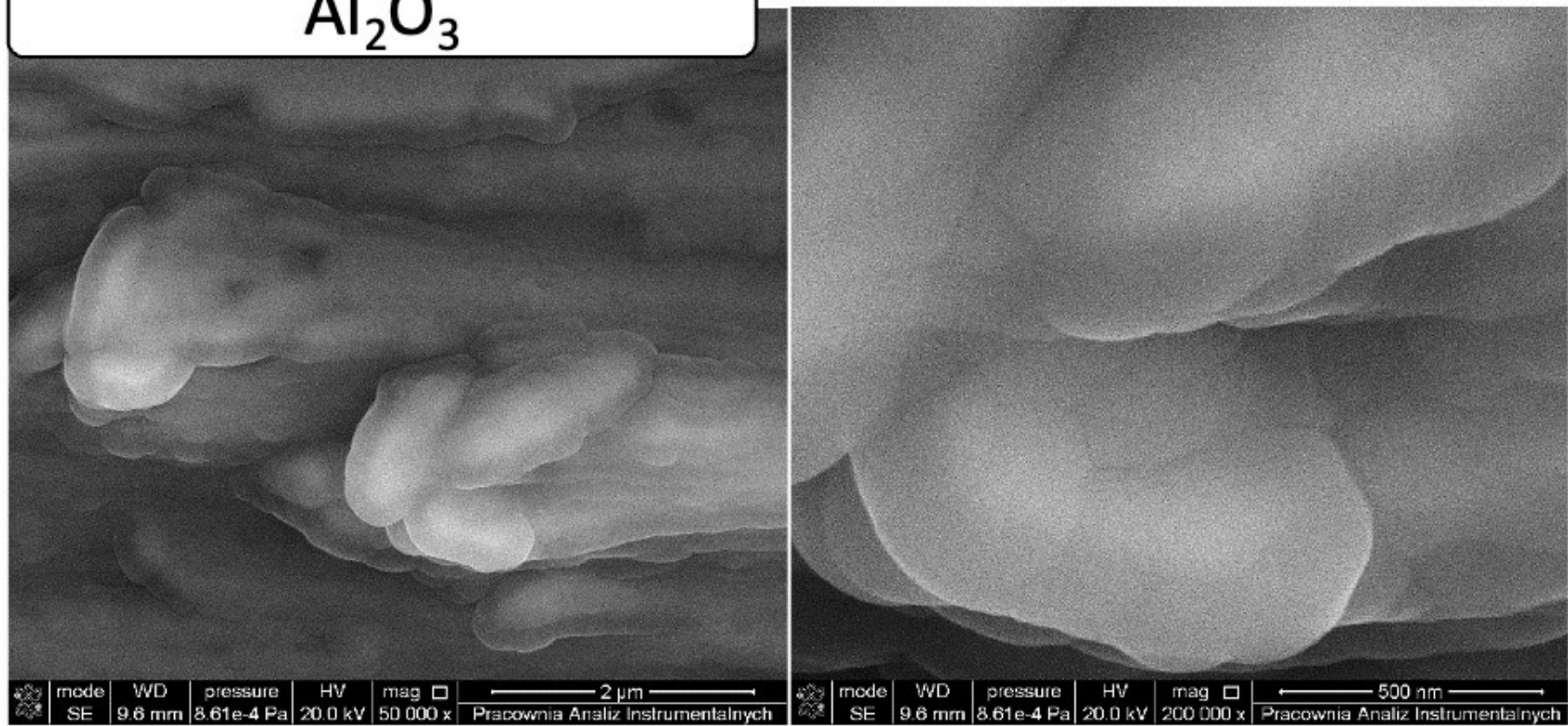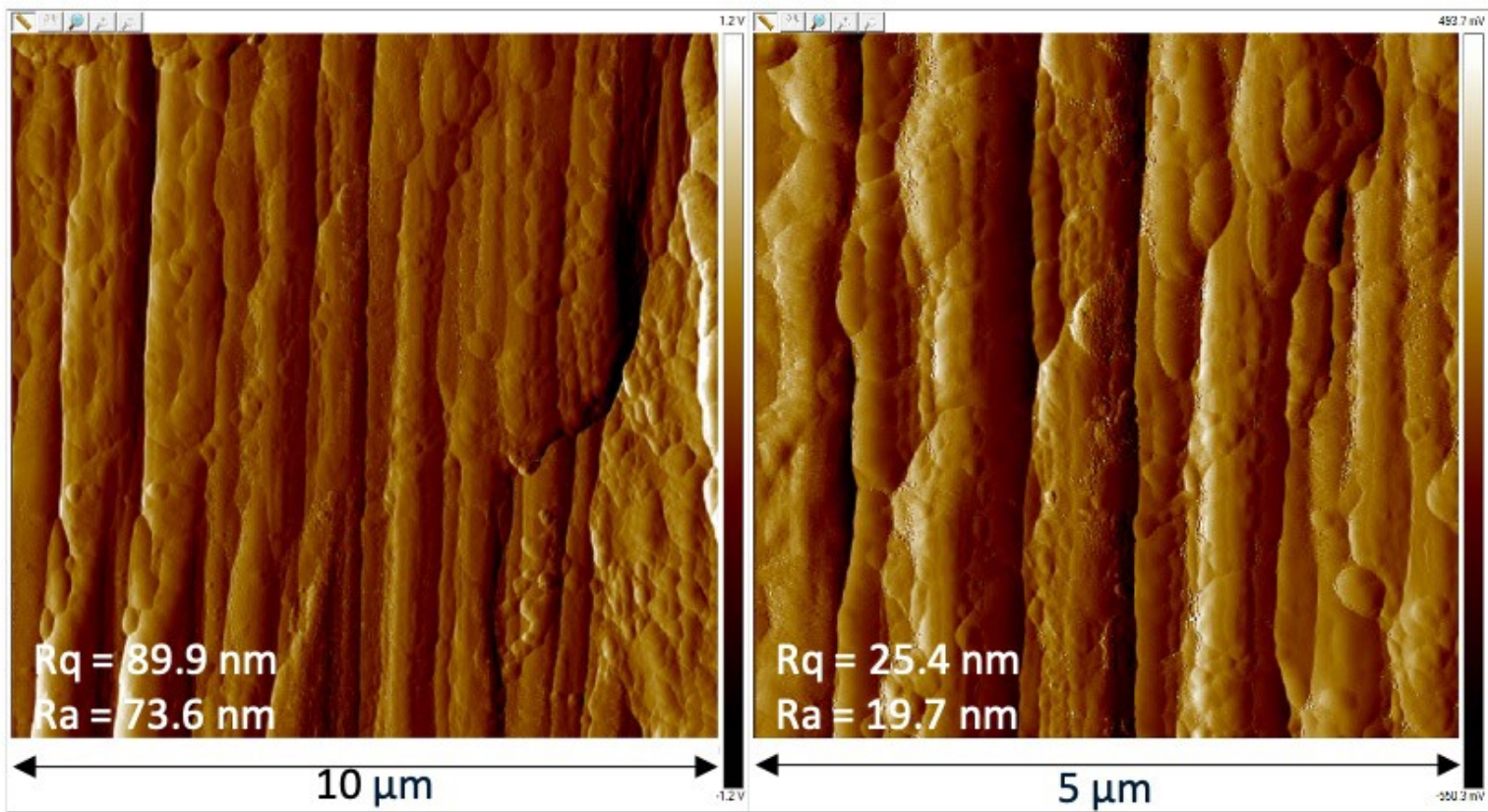

$\text{Al}_2\text{O}_3 + \text{AgNPs}$

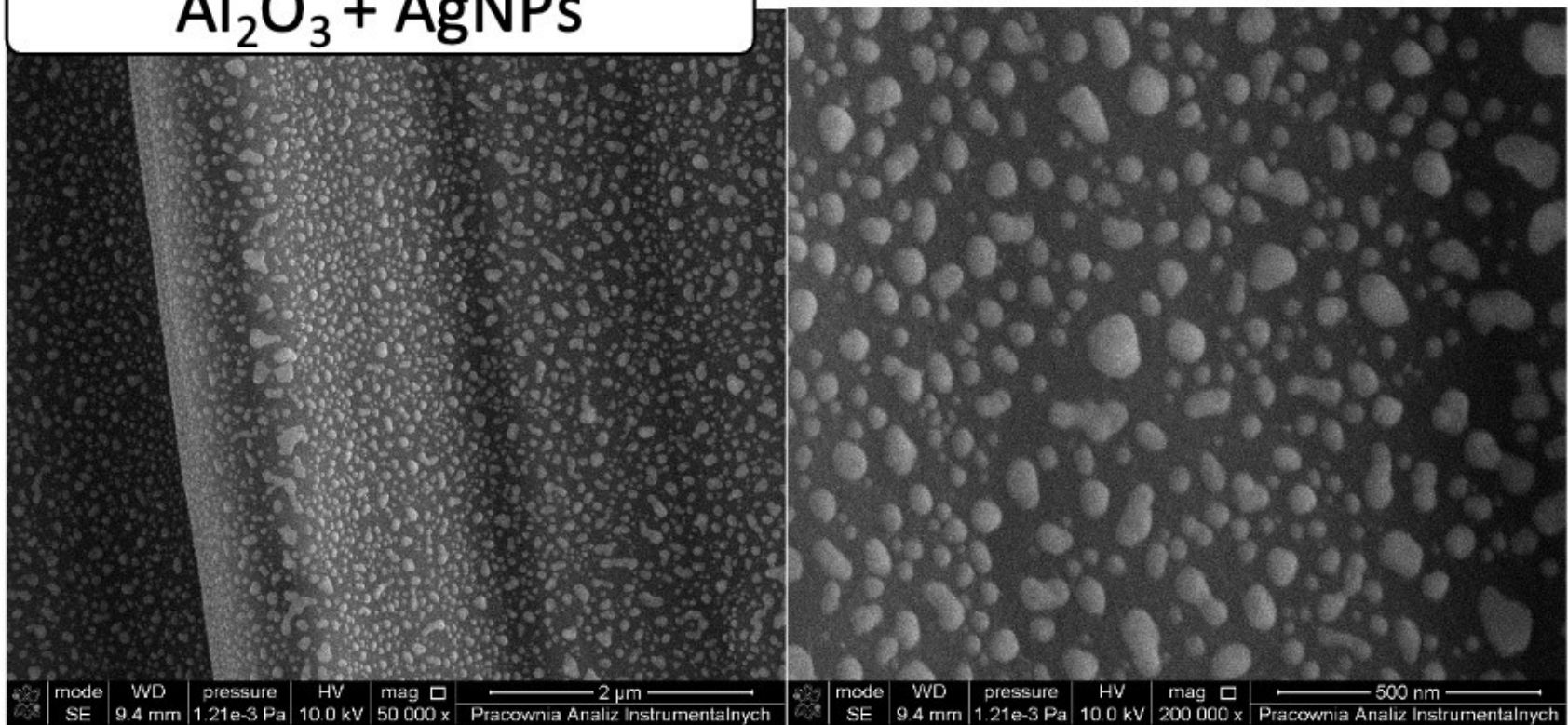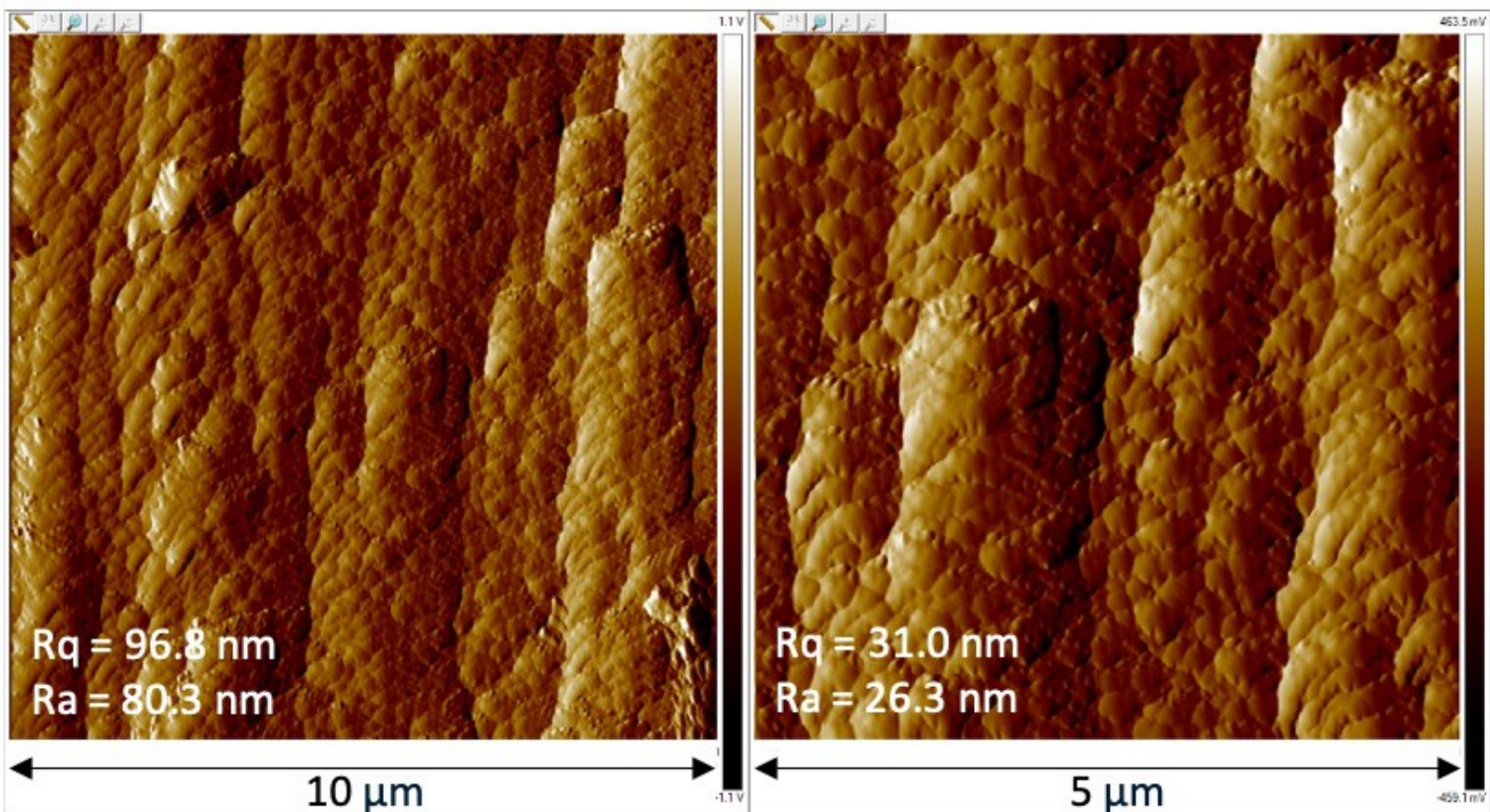

Supplement: RA-016-D5RA09570K-s005 [file RA-016-D5RA09570K-s005.pdf]

$\text{Al}_2\text{O}_3$

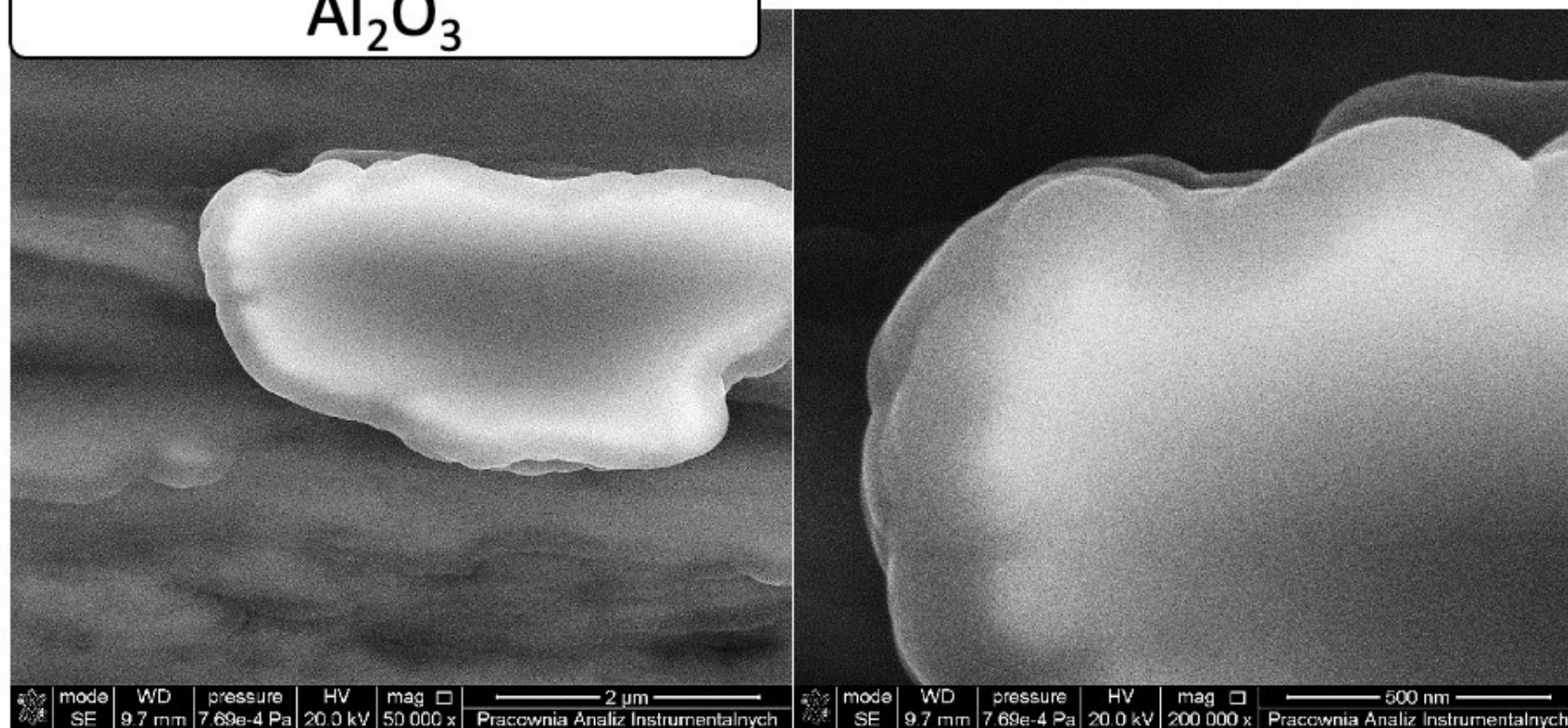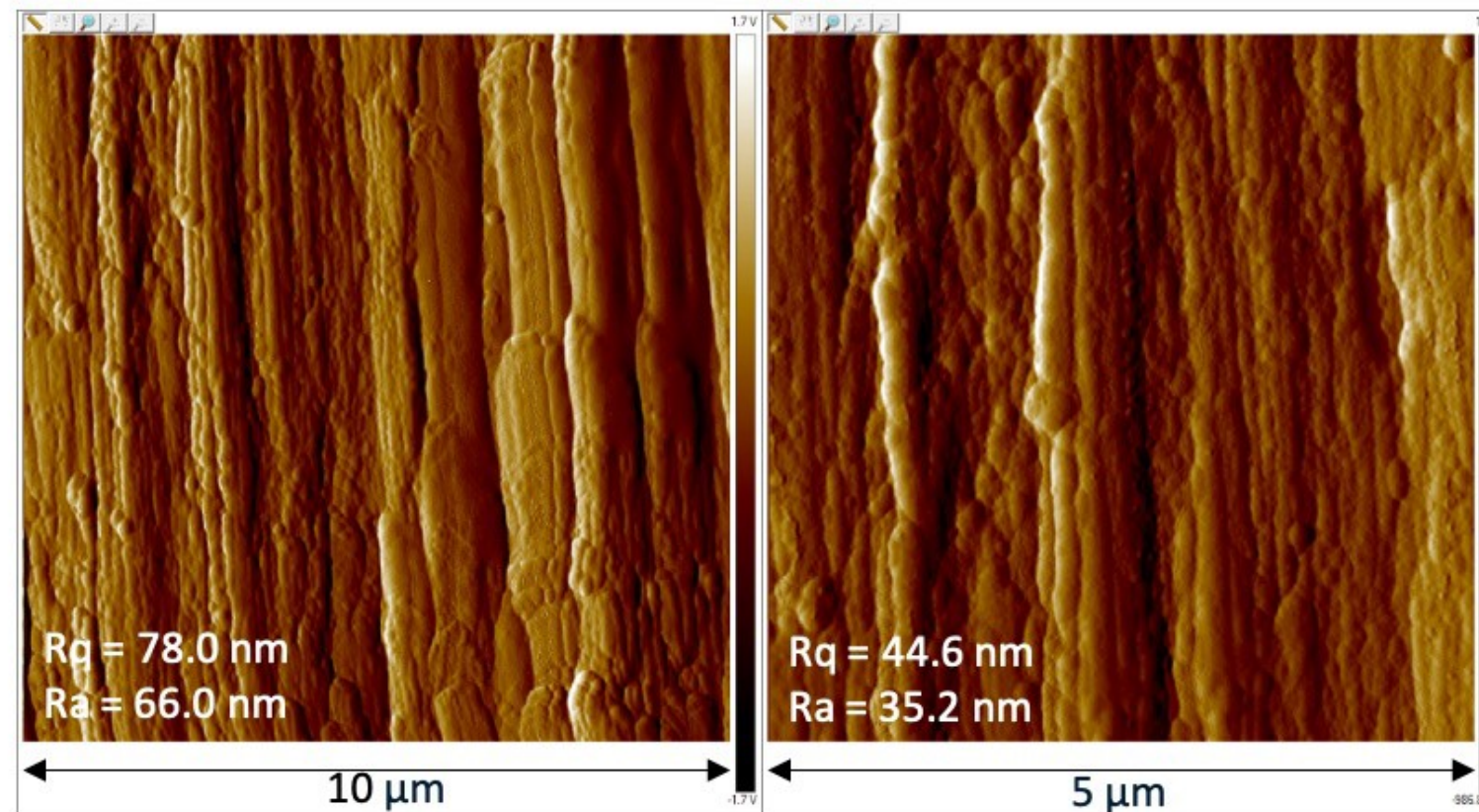

$\text{Al}_2\text{O}_3 + \text{AgNPs}$

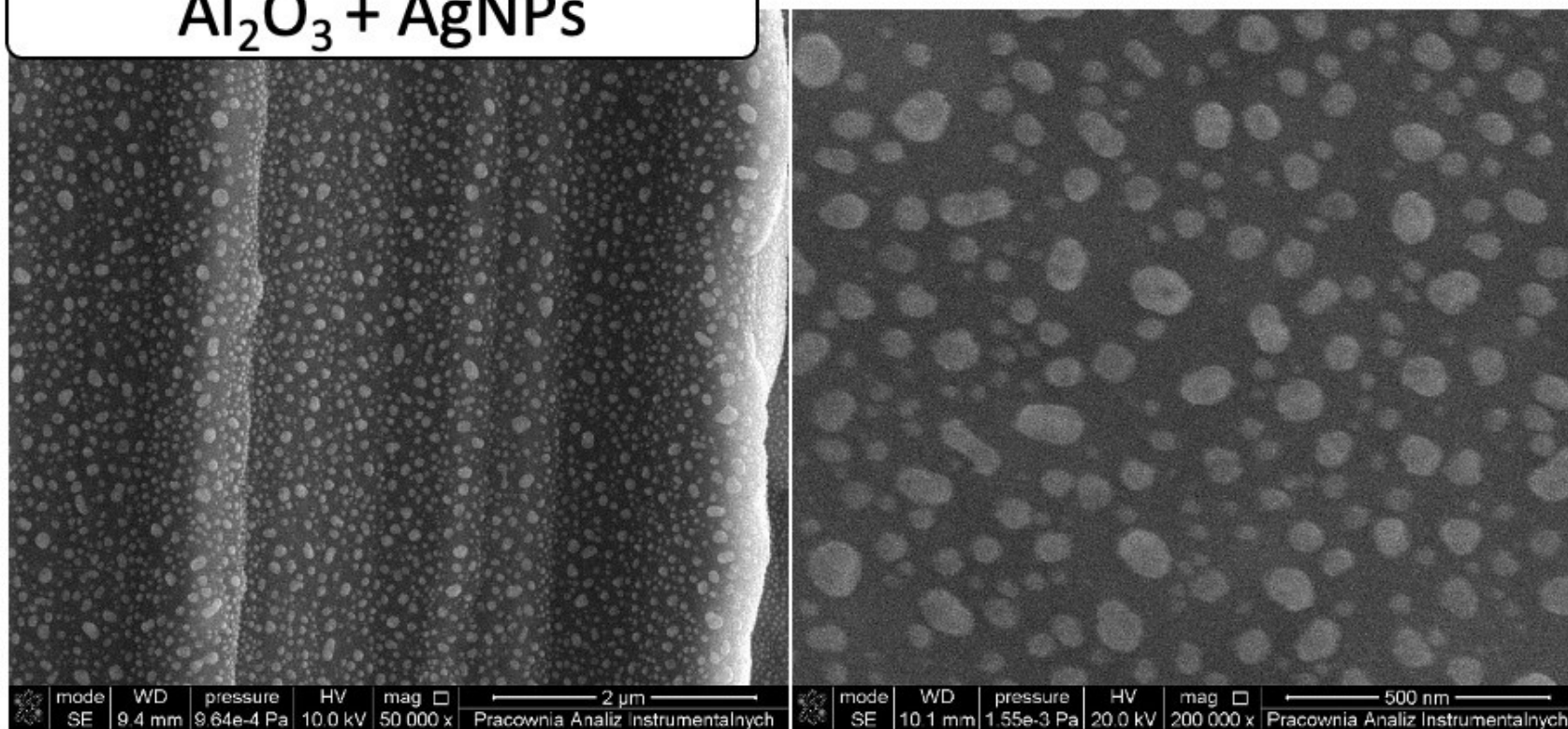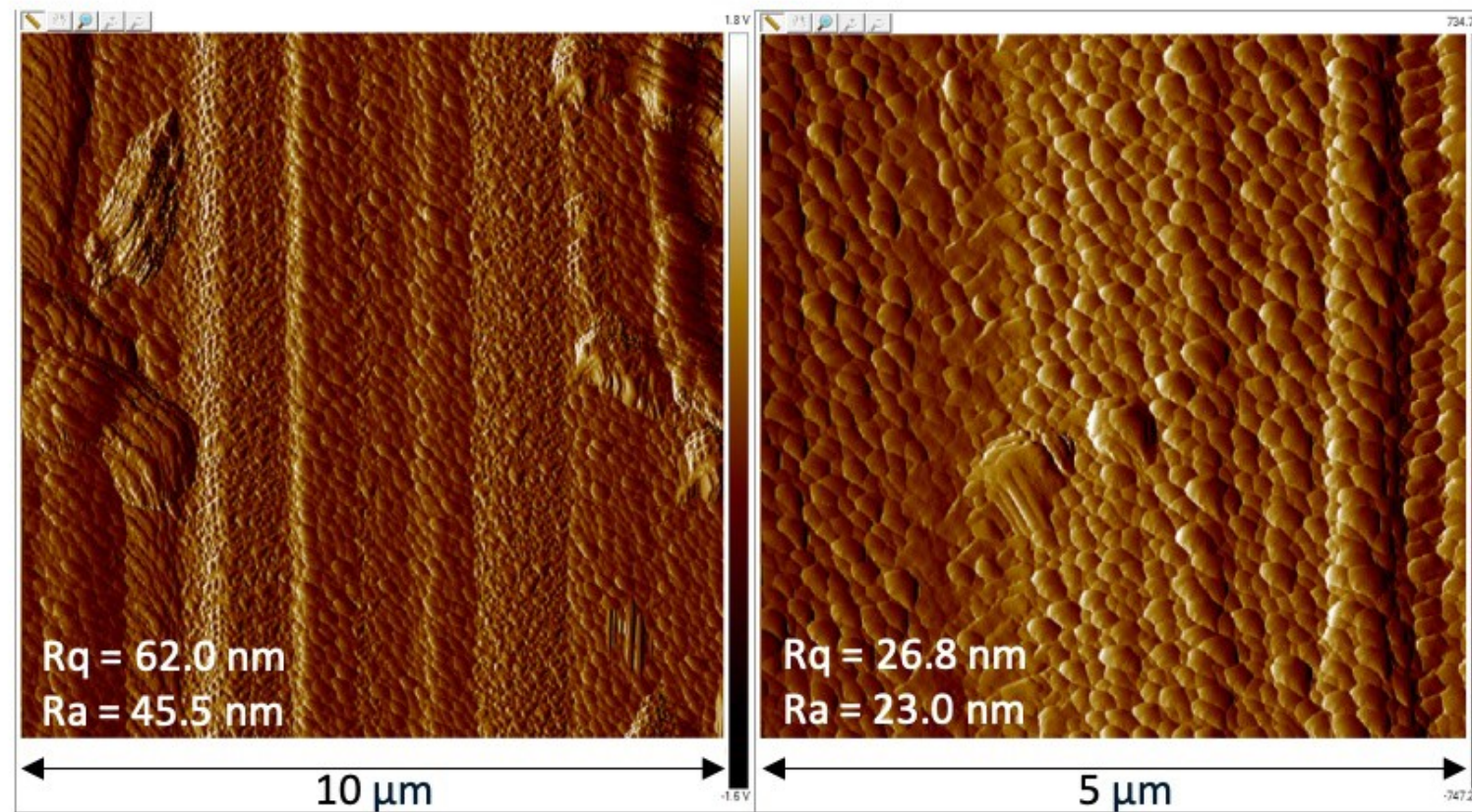

Supplement: RA-016-D5RA09570K-s006 [file RA-016-D5RA09570K-s006.pdf]

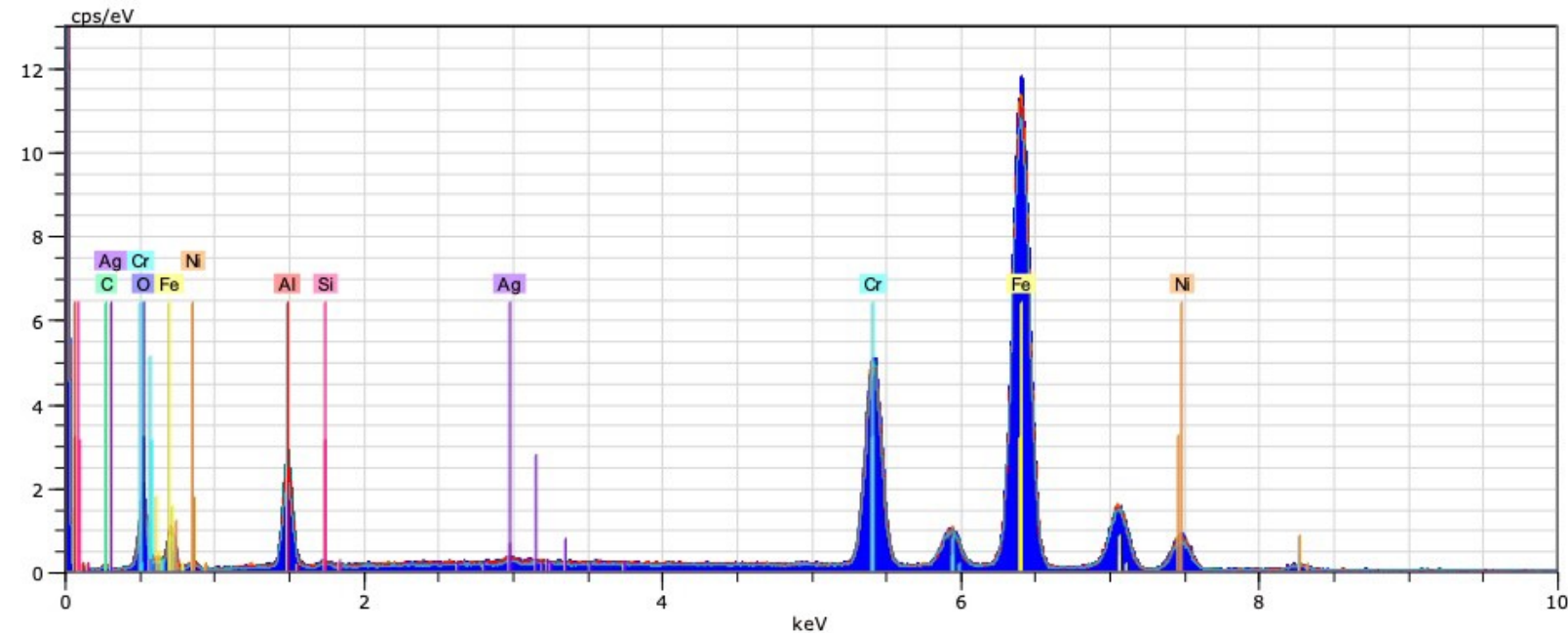

Atomic percent (%)

| Spectrum    | C    | O     | Al    | Si   | Cr    | Fe    | Ni   | Ag   |
|-------------|------|-------|-------|------|-------|-------|------|------|
| 58719       | 2.80 | 11.48 | 14.28 | 0.38 | 14.19 | 51.34 | 5.21 | 0.32 |
| 58720       | 3.06 | 13.22 | 14.88 | 0.40 | 13.81 | 49.34 | 4.96 | 0.34 |
| 58721       | 2.87 | 14.05 | 14.62 | 0.42 | 13.24 | 48.98 | 5.54 | 0.27 |
| 58722       | 2.96 | 12.45 | 12.83 | 0.33 | 14.25 | 51.29 | 5.61 | 0.28 |
| 58723       | 3.28 | 13.96 | 14.26 | 0.48 | 13.63 | 48.89 | 5.22 | 0.29 |
| Mean value: | 2.99 | 13.03 | 14.18 | 0.40 | 13.82 | 49.97 | 5.31 | 0.30 |
| Sigma:      | 0.19 | 1.08  | 0.79  | 0.05 | 0.42  | 1.24  | 0.27 | 0.03 |
| Sigma mean: | 0.08 | 0.48  | 0.35  | 0.02 | 0.19  | 0.55  | 0.12 | 0.01 |

Supplement: RA-016-D5RA09570K-s007 [file RA-016-D5RA09570K-s007.pdf]

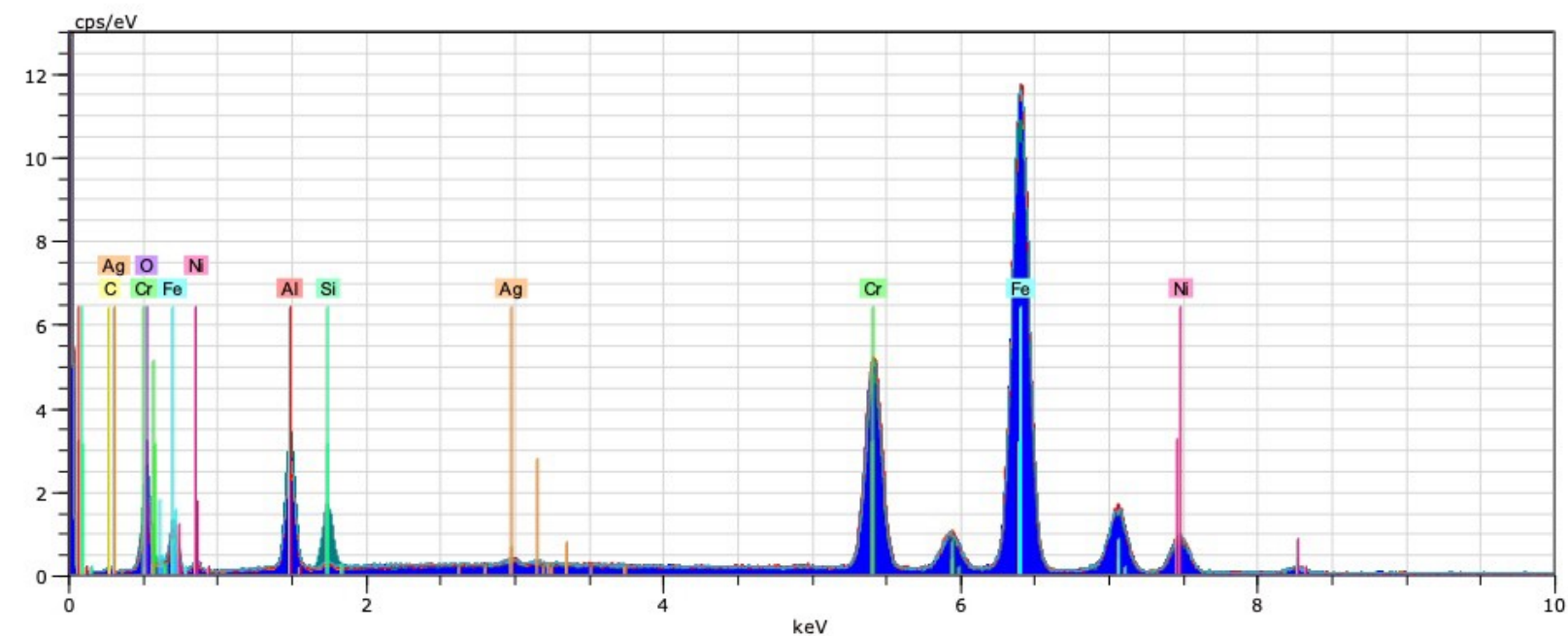

Atomic percent (%)

| Spectrum    | C    | O     | Al    | Si   | Cr    | Fe    | Ni   | Ag   |
|-------------|------|-------|-------|------|-------|-------|------|------|
| 58714       | 3.21 | 14.31 | 12.18 | 0.43 | 13.83 | 50.14 | 5.61 | 0.30 |
| 58715       | 3.40 | 14.40 | 14.08 | 0.47 | 13.82 | 48.37 | 5.13 | 0.33 |
| 58716       | 3.21 | 19.59 | 15.28 | 6.37 | 11.36 | 39.79 | 4.10 | 0.30 |
| 58717       | 3.23 | 13.14 | 14.95 | 0.46 | 13.96 | 48.90 | 5.05 | 0.31 |
| 58718       | 3.15 | 14.53 | 13.76 | 0.43 | 13.62 | 48.93 | 5.26 | 0.31 |
| Mean value: | 3.24 | 15.19 | 14.05 | 1.63 | 13.32 | 47.23 | 5.03 | 0.31 |
| Sigma:      | 0.09 | 2.52  | 1.22  | 2.65 | 1.10  | 4.21  | 0.56 | 0.01 |
| Sigma mean: | 0.04 | 1.13  | 0.54  | 1.18 | 0.49  | 1.88  | 0.25 | 0.00 |

Supplement: RA-016-D5RA09570K-s008 [file RA-016-D5RA09570K-s008.pdf]

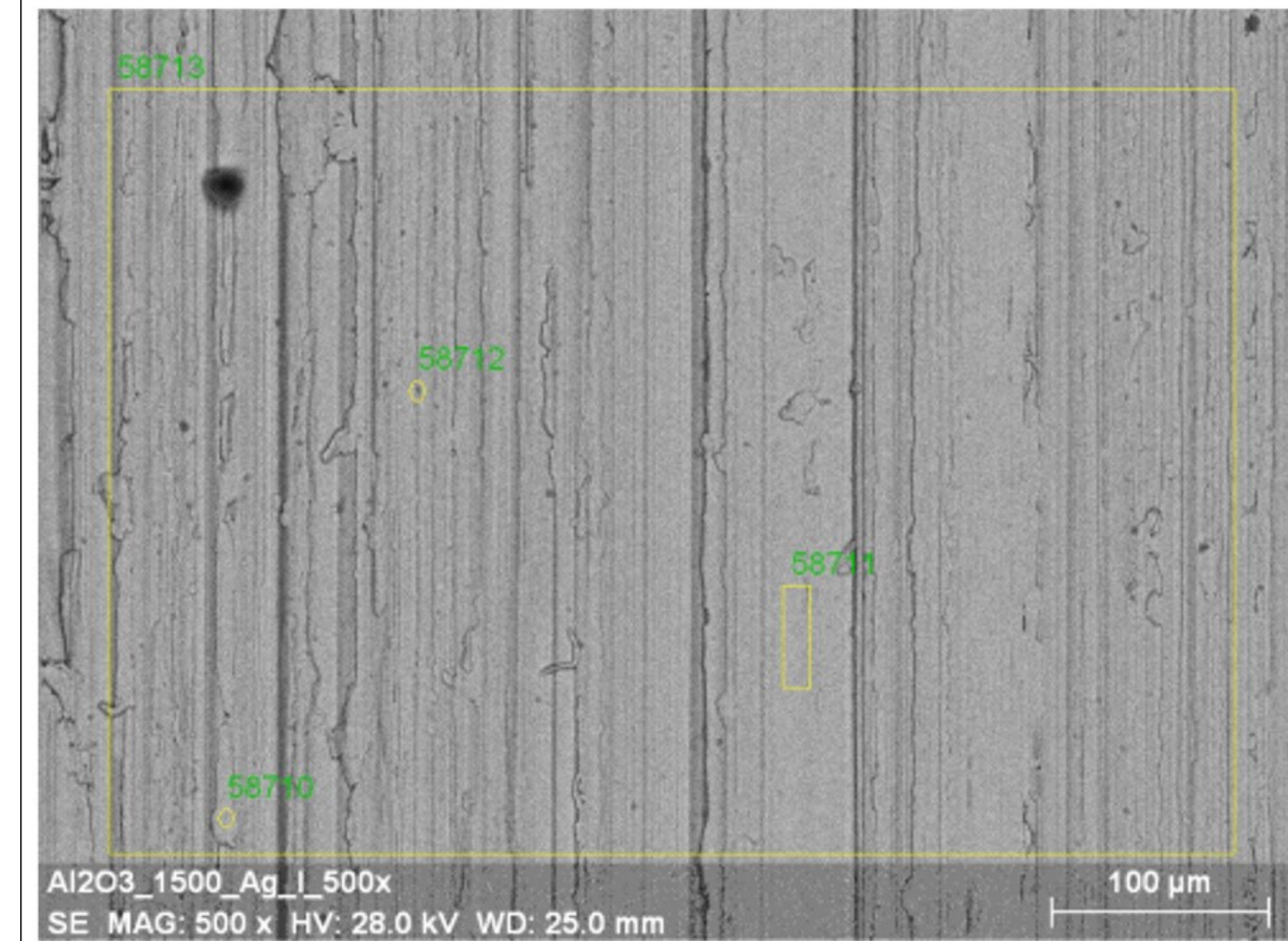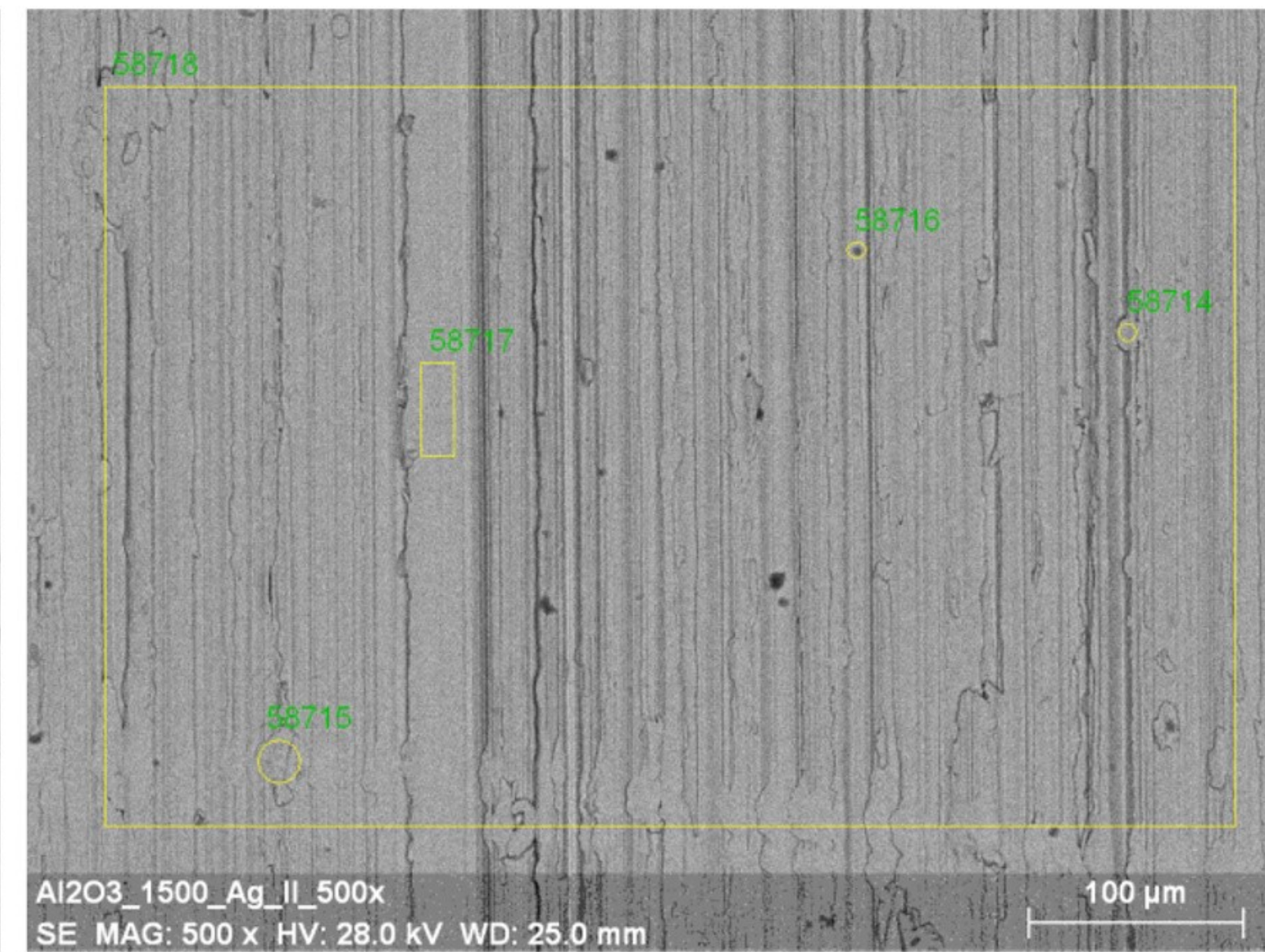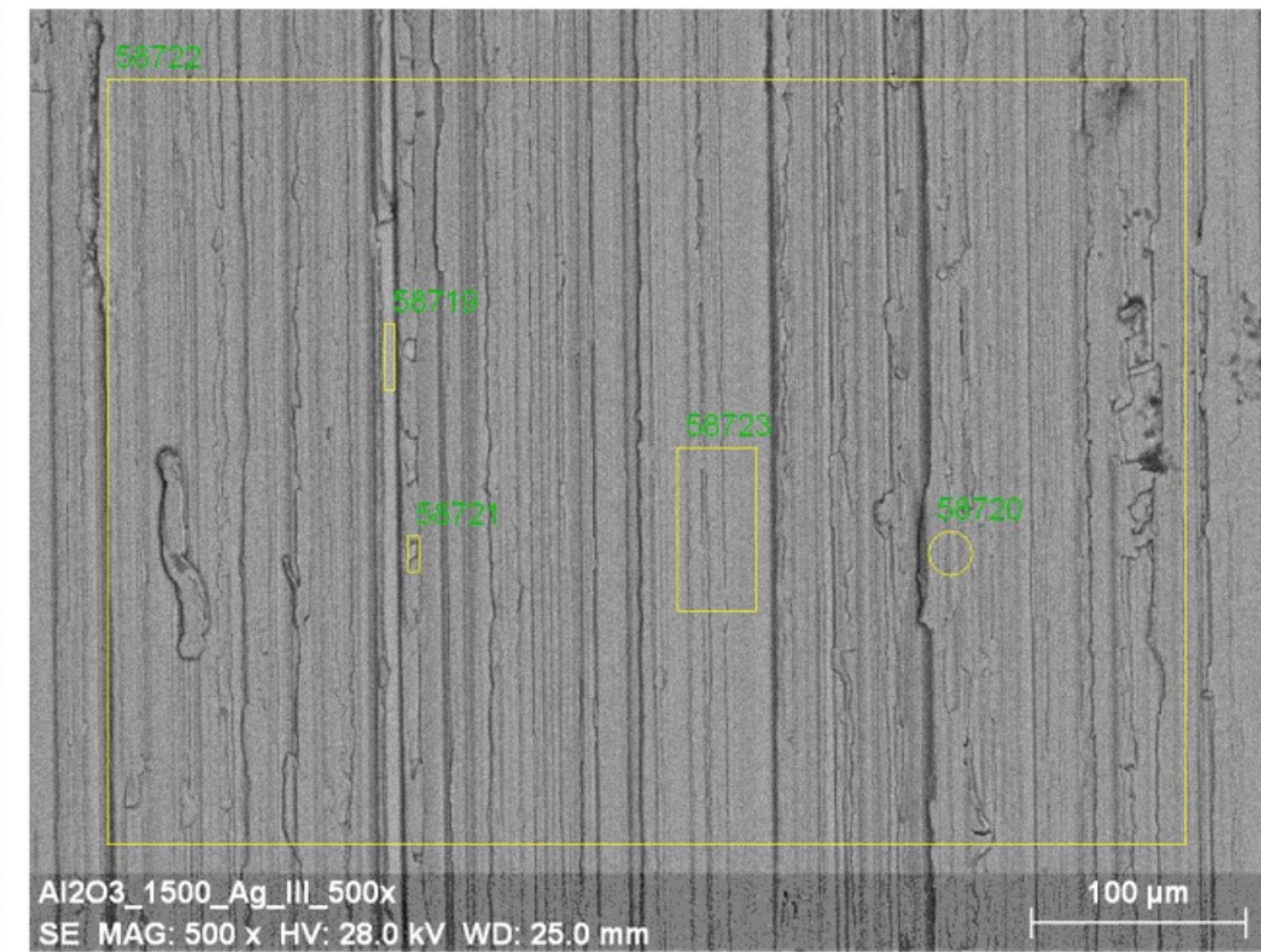

Supplement: RA-016-D5RA09570K-s009 [file RA-016-D5RA09570K-s009.pdf]

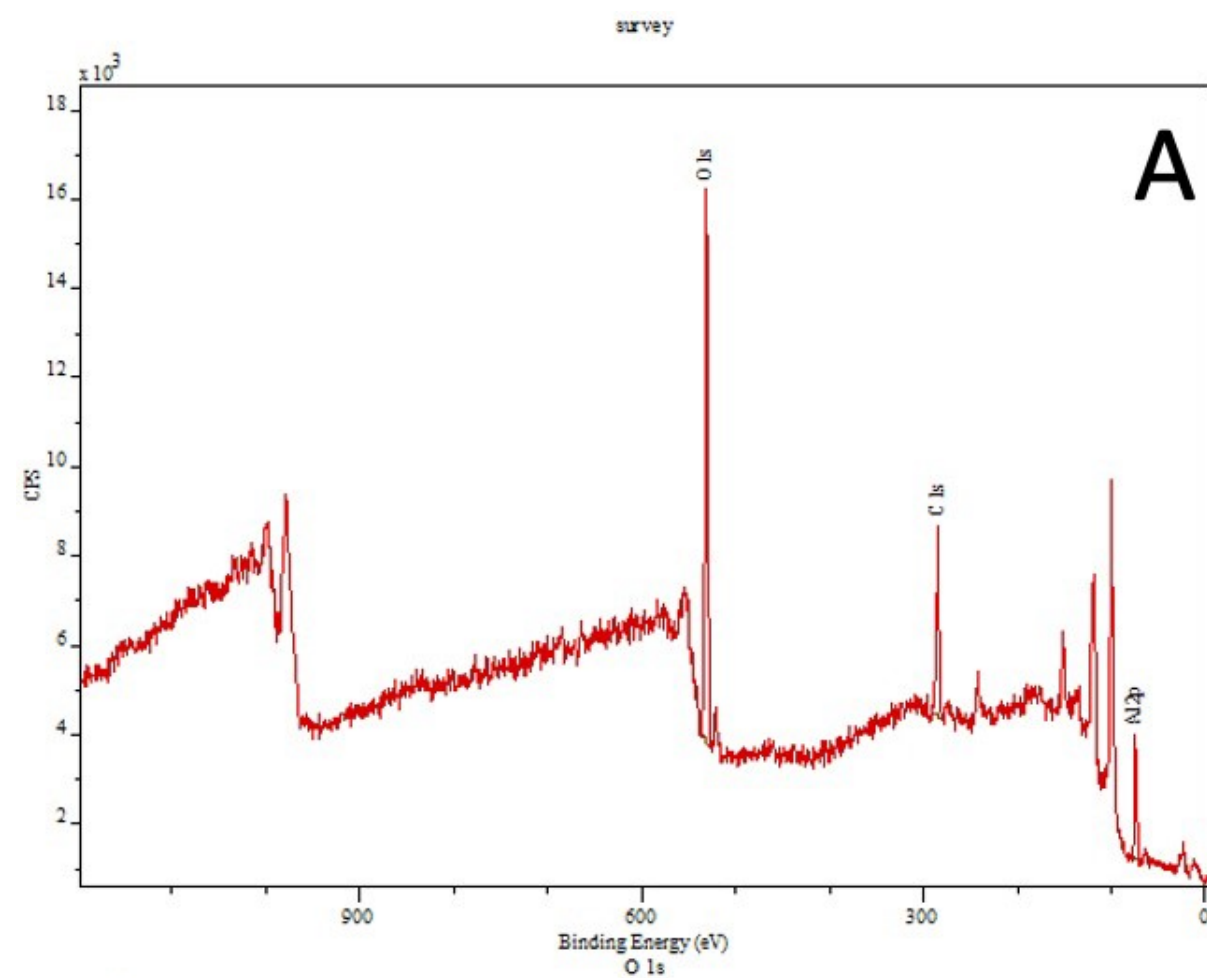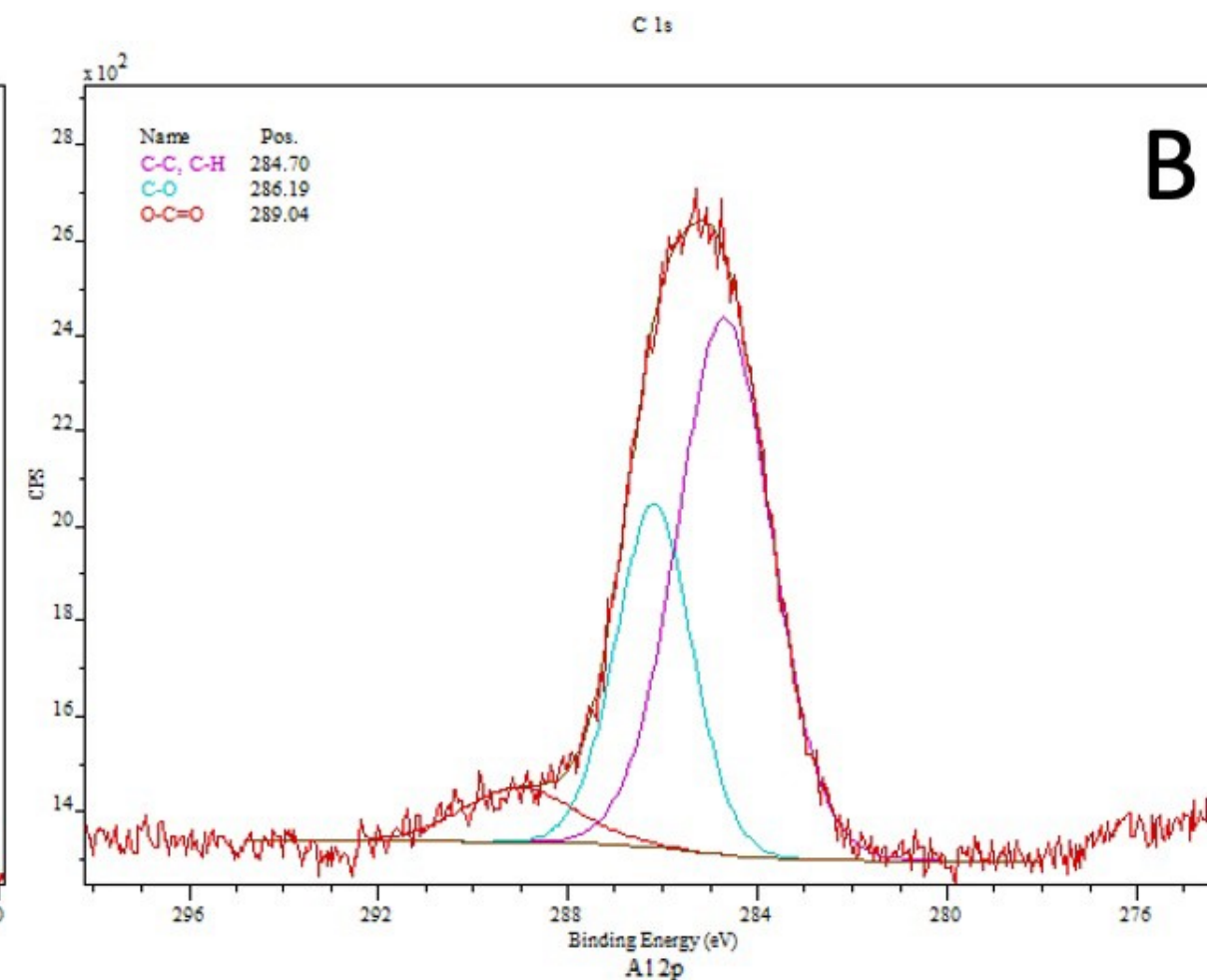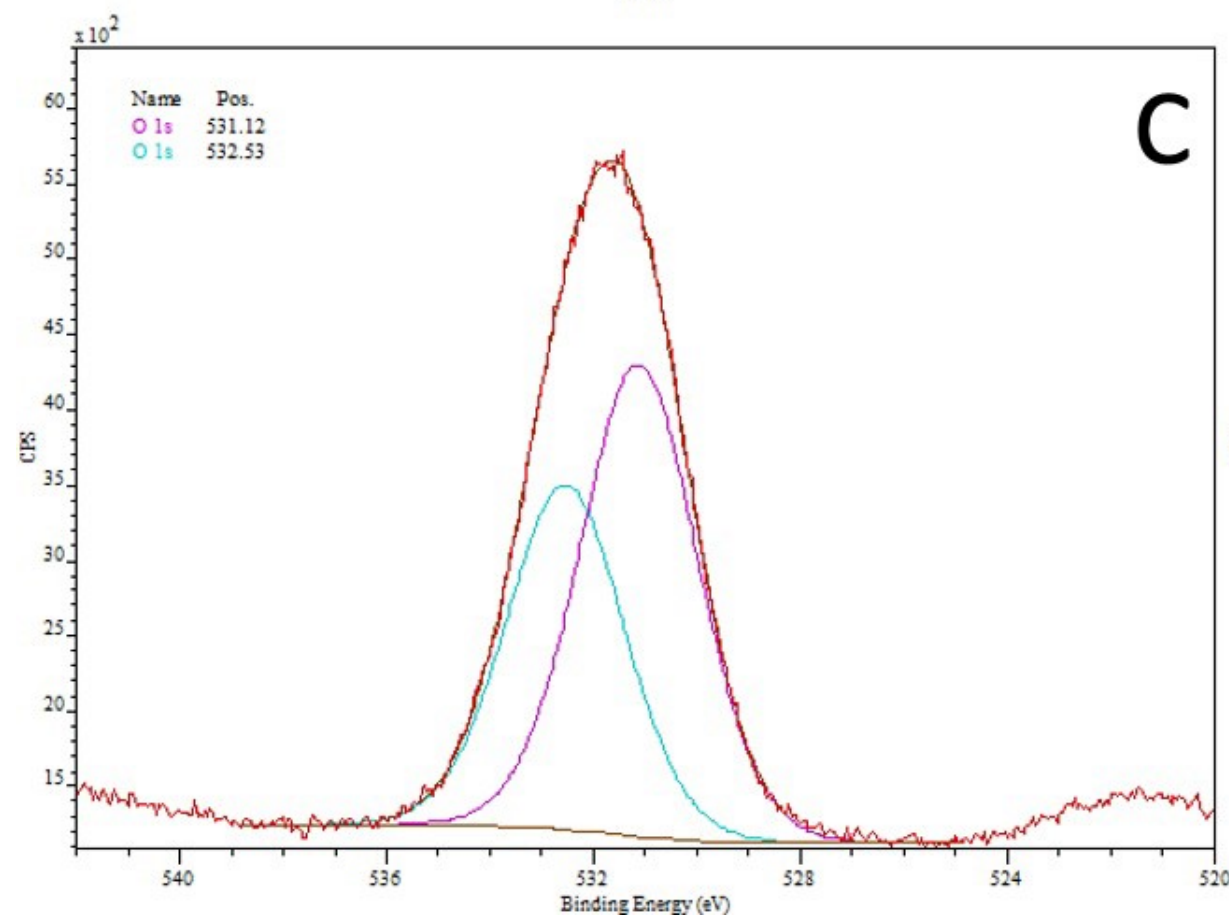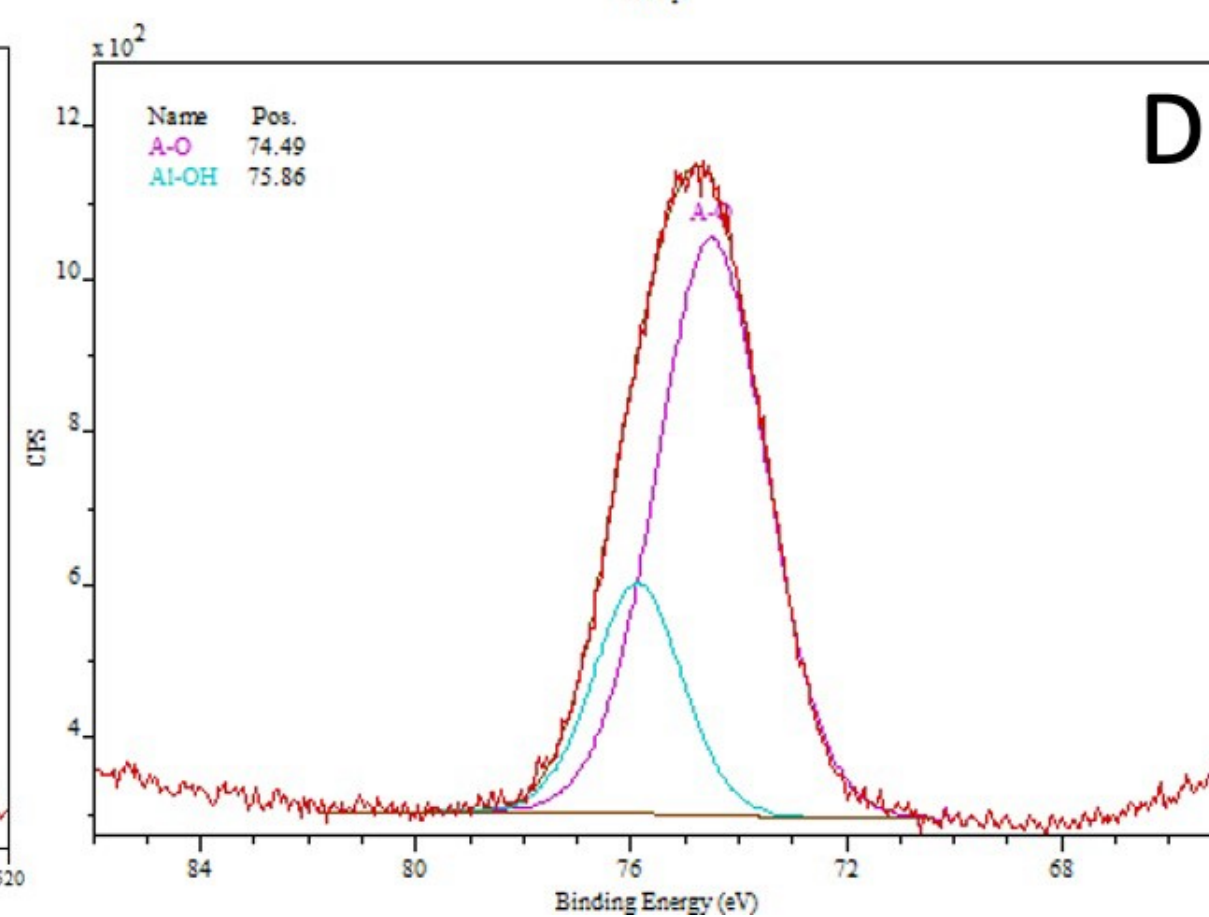

Supplement: RA-016-D5RA09570K-s010 [file RA-016-D5RA09570K-s010.pdf]

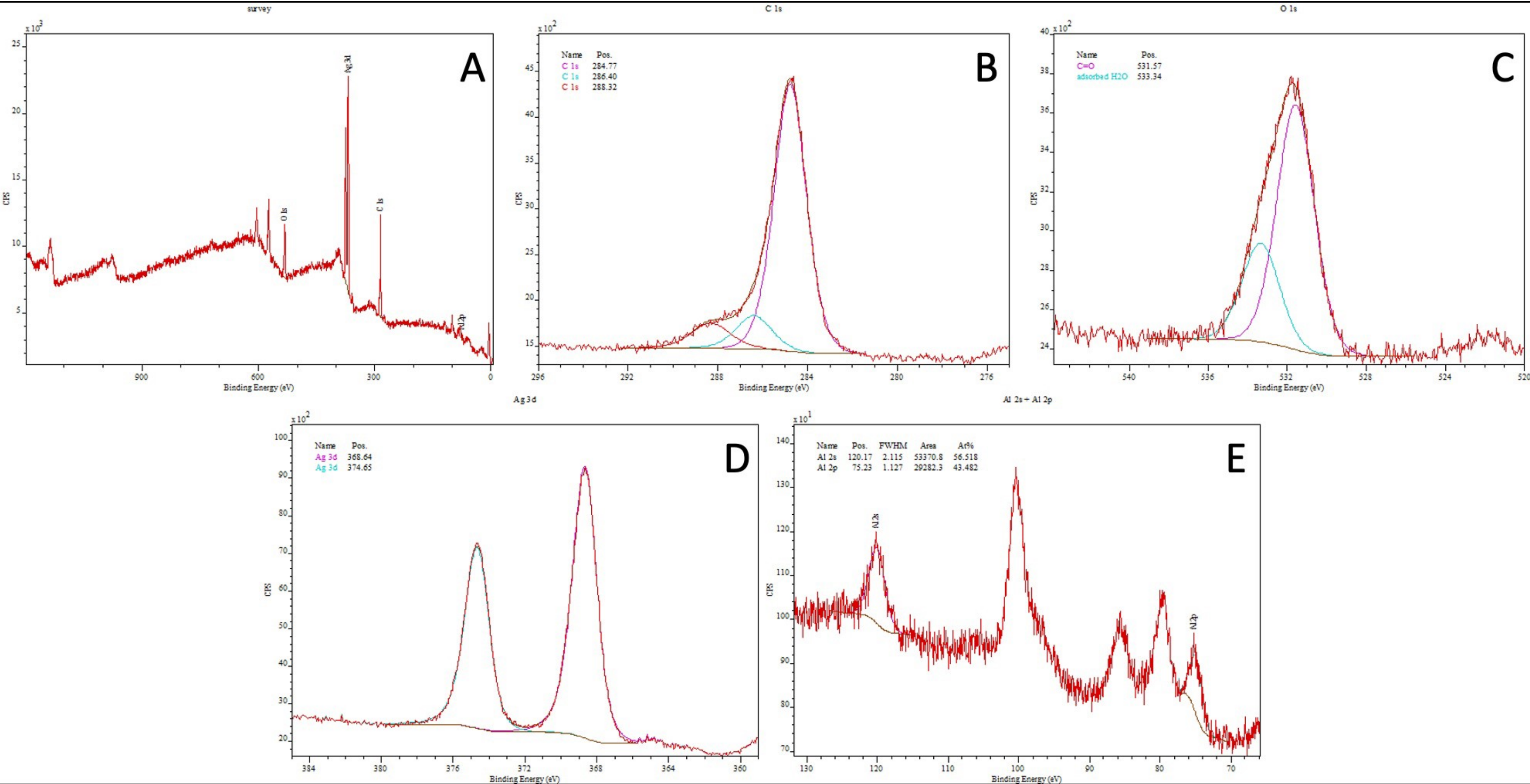

Supplement: RA-016-D5RA09570K-s011 [file RA-016-D5RA09570K-s011.pdf]

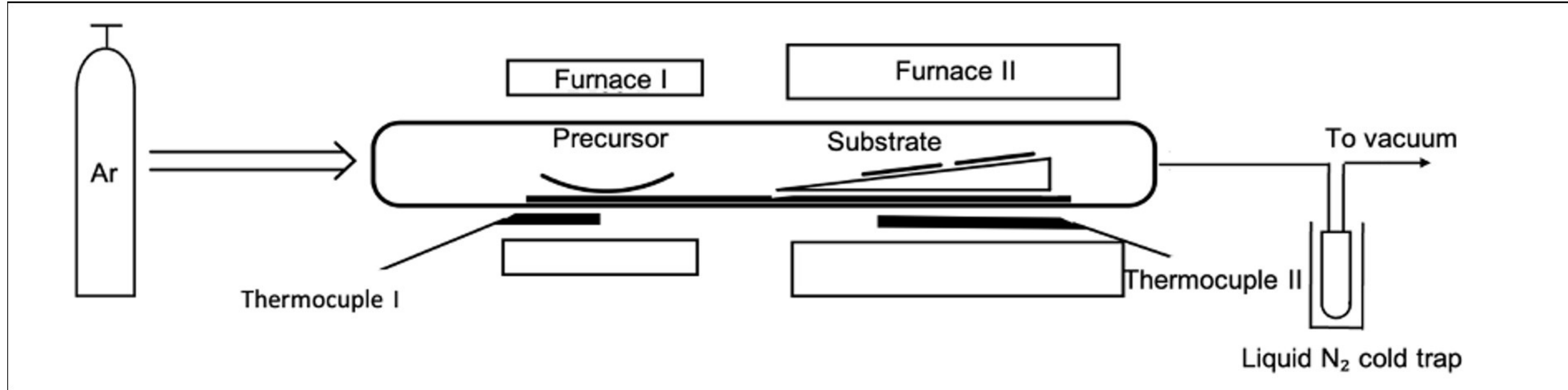

Supplement: RA-016-D5RA09570K-s012 [file RA-016-D5RA09570K-s012.pdf]

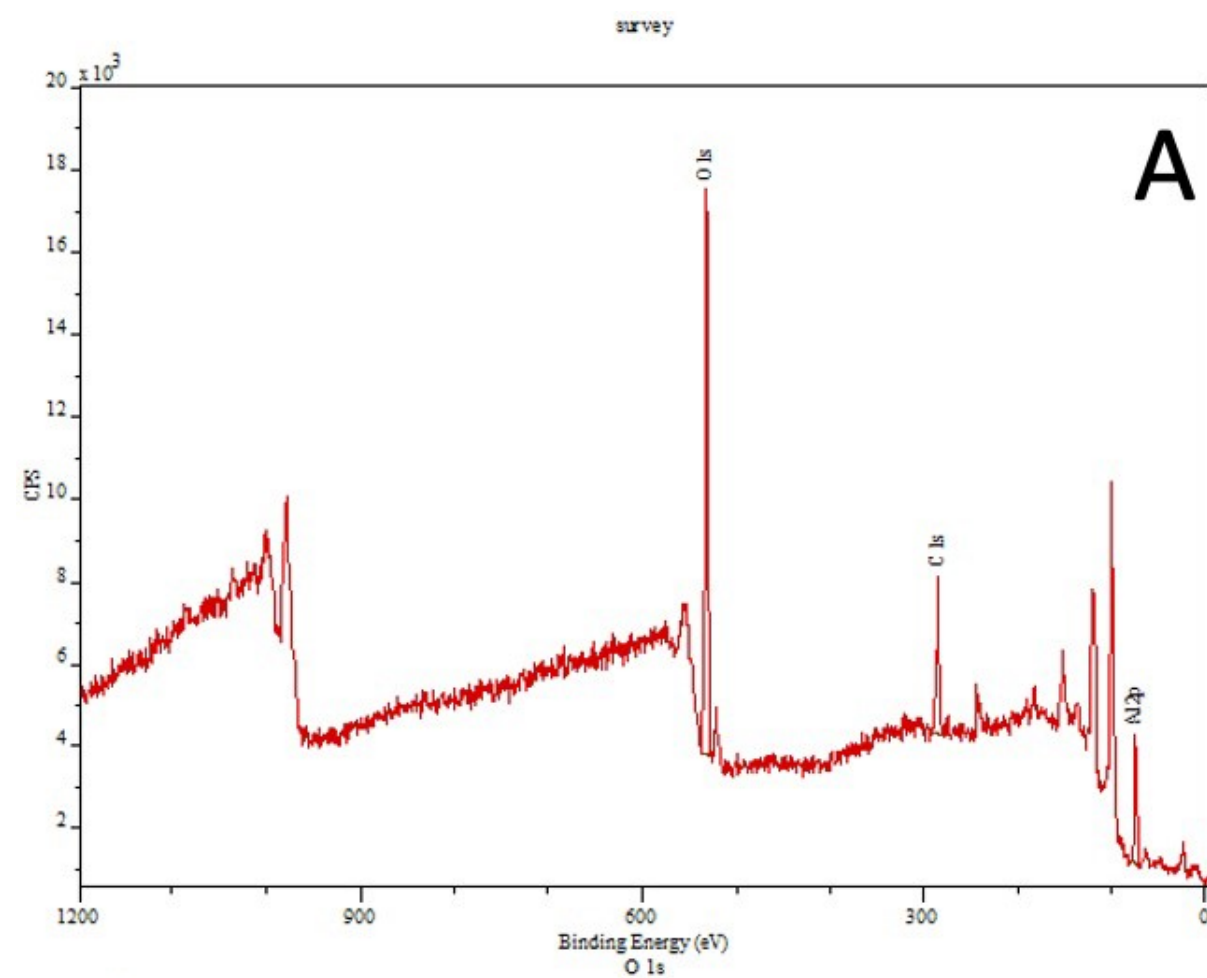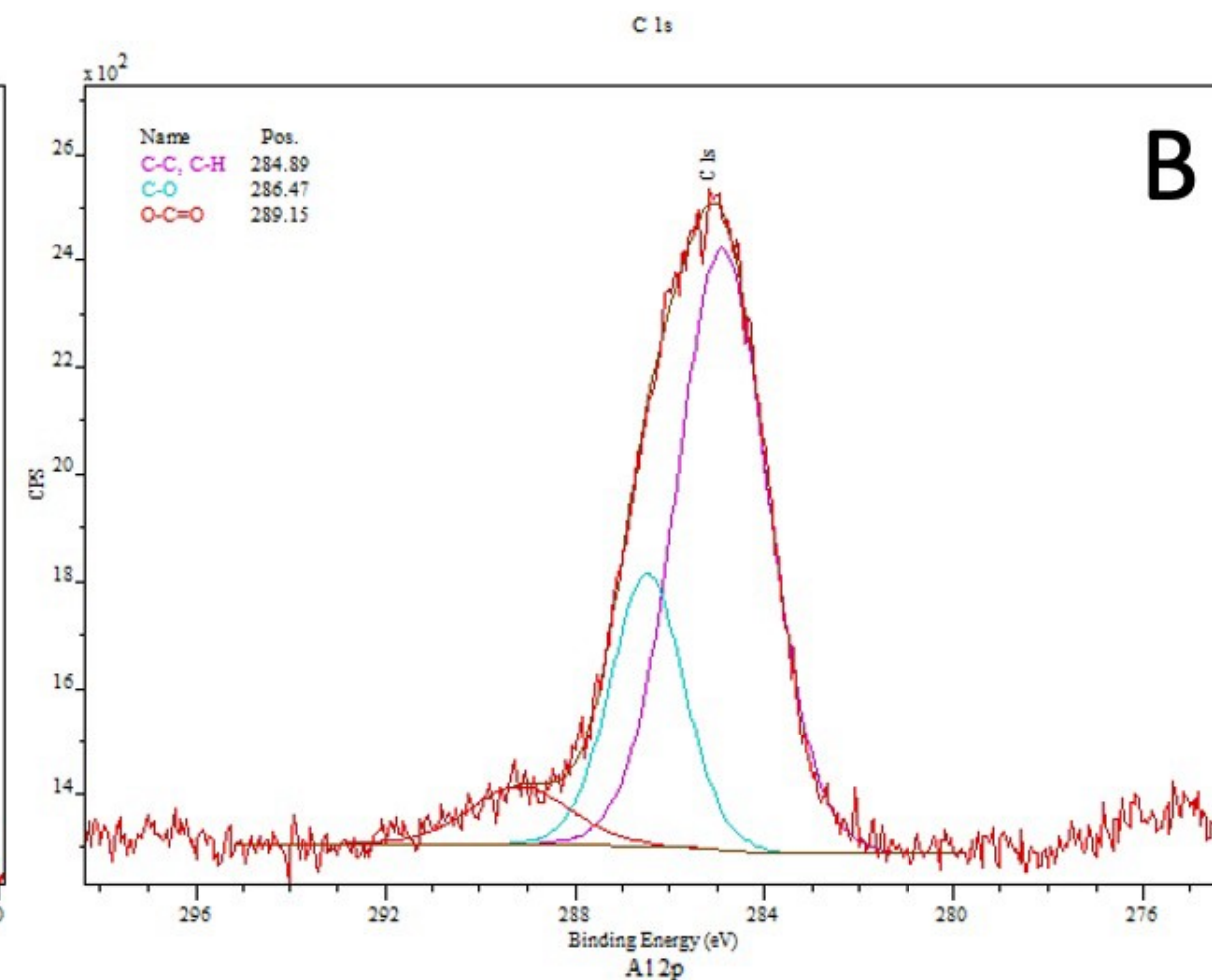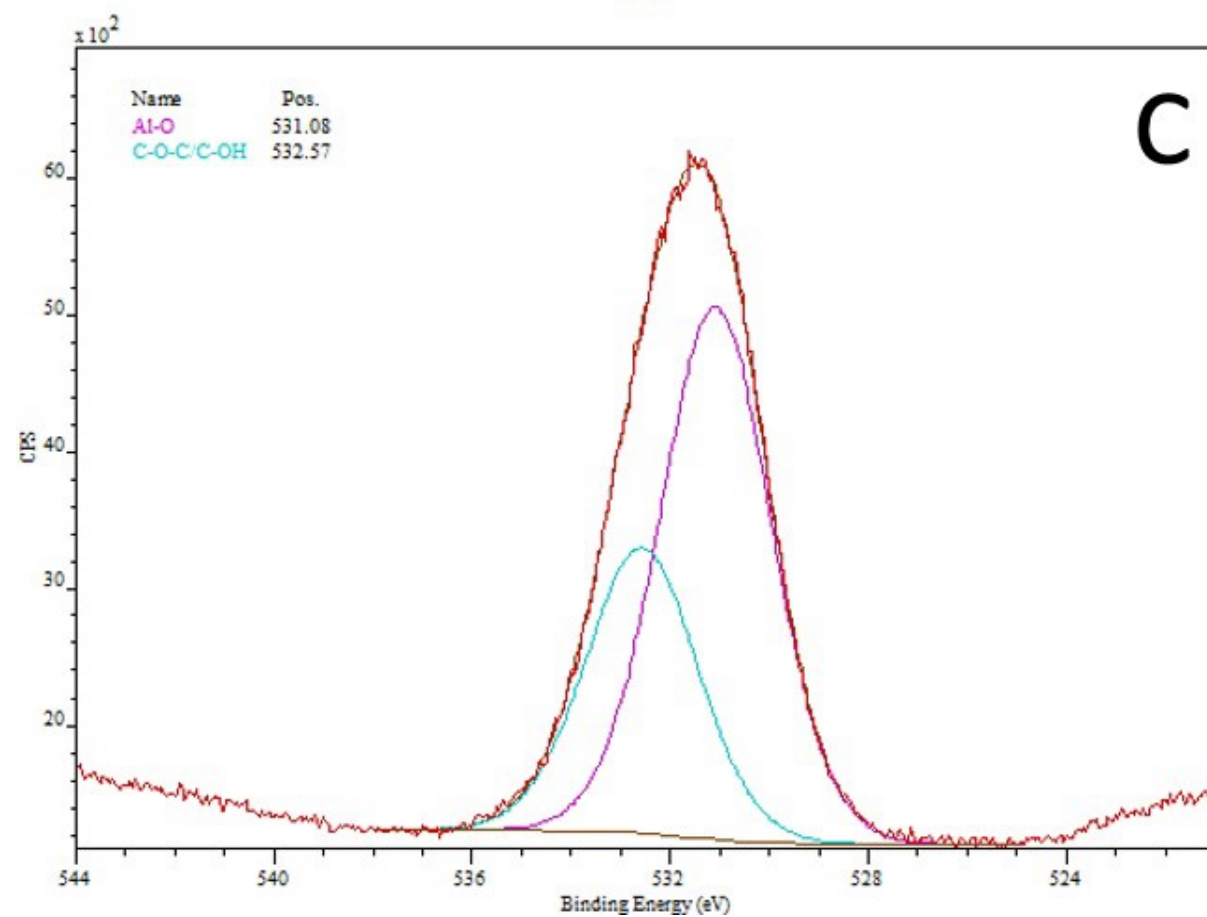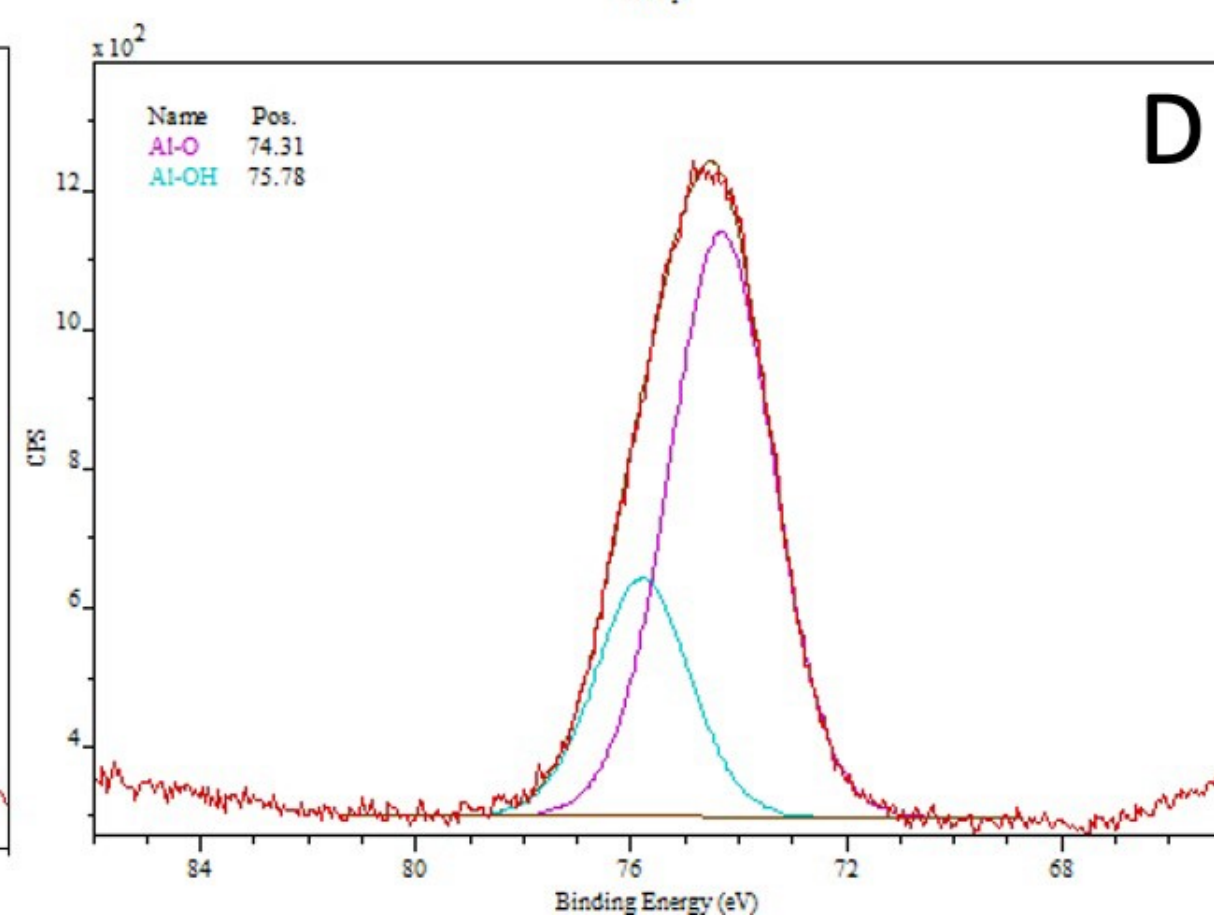

Supplement: RA-016-D5RA09570K-s013 [file RA-016-D5RA09570K-s013.pdf]

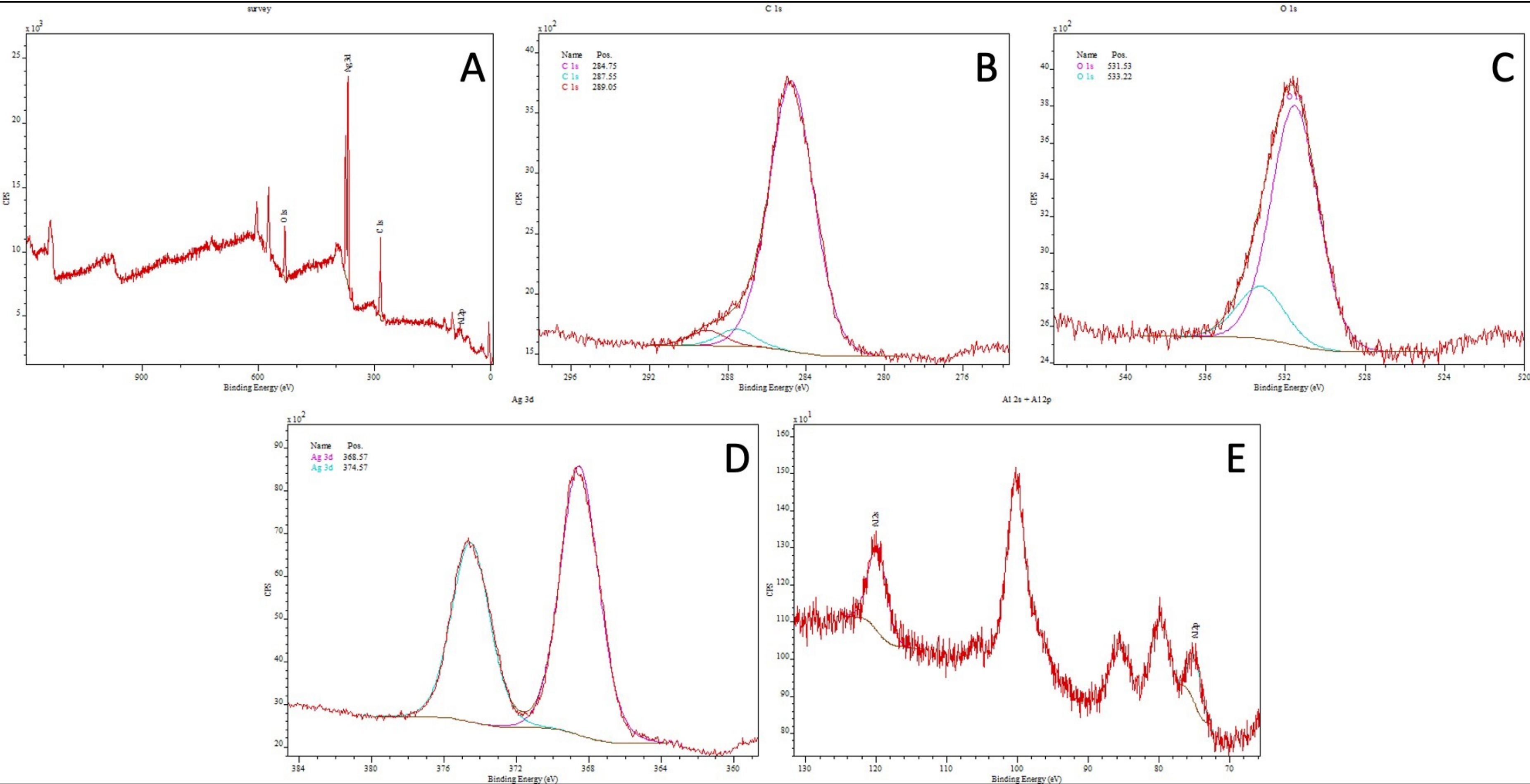

Supplement: RA-016-D5RA09570K-s015 [file RA-016-D5RA09570K-s015.pdf]

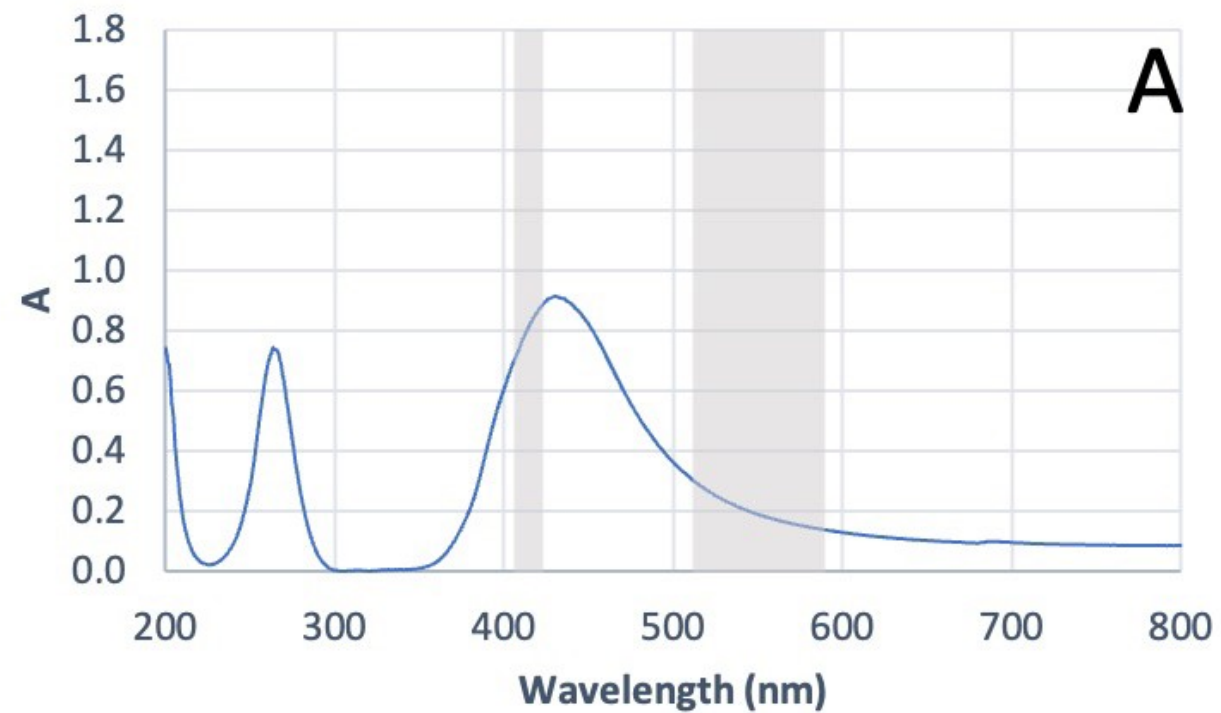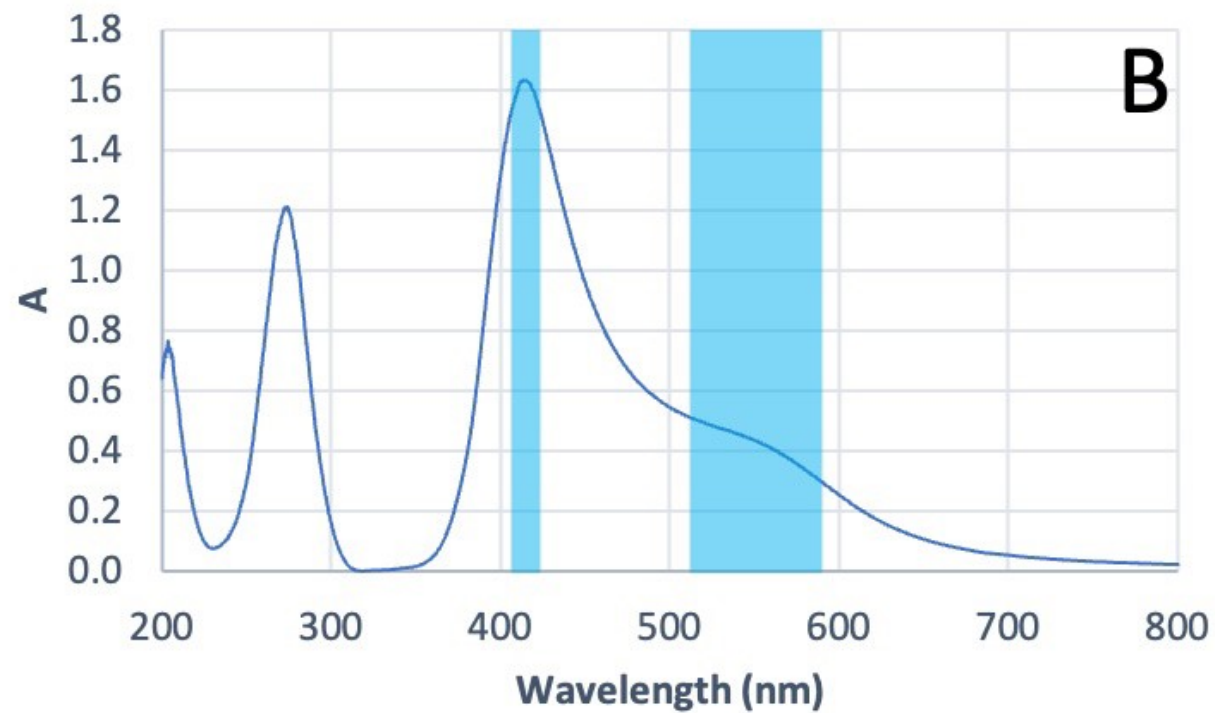

Supplement: RA-016-D5RA09570K-s016 [file RA-016-D5RA09570K-s016.pdf]

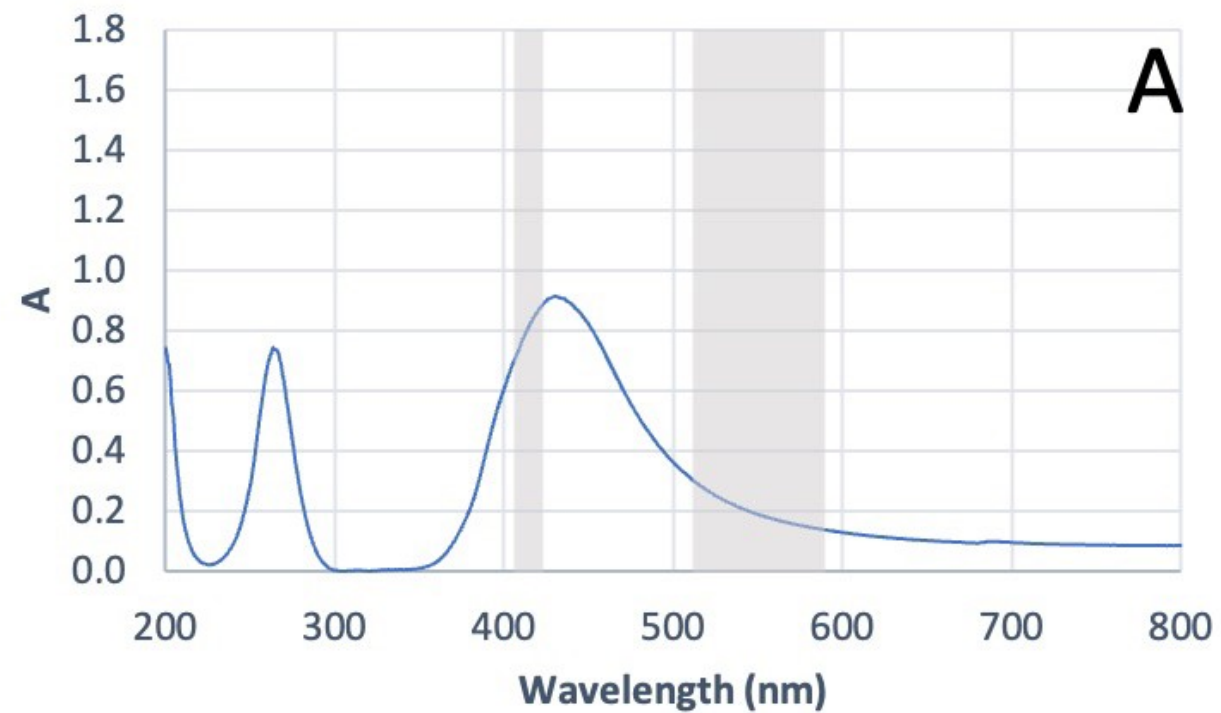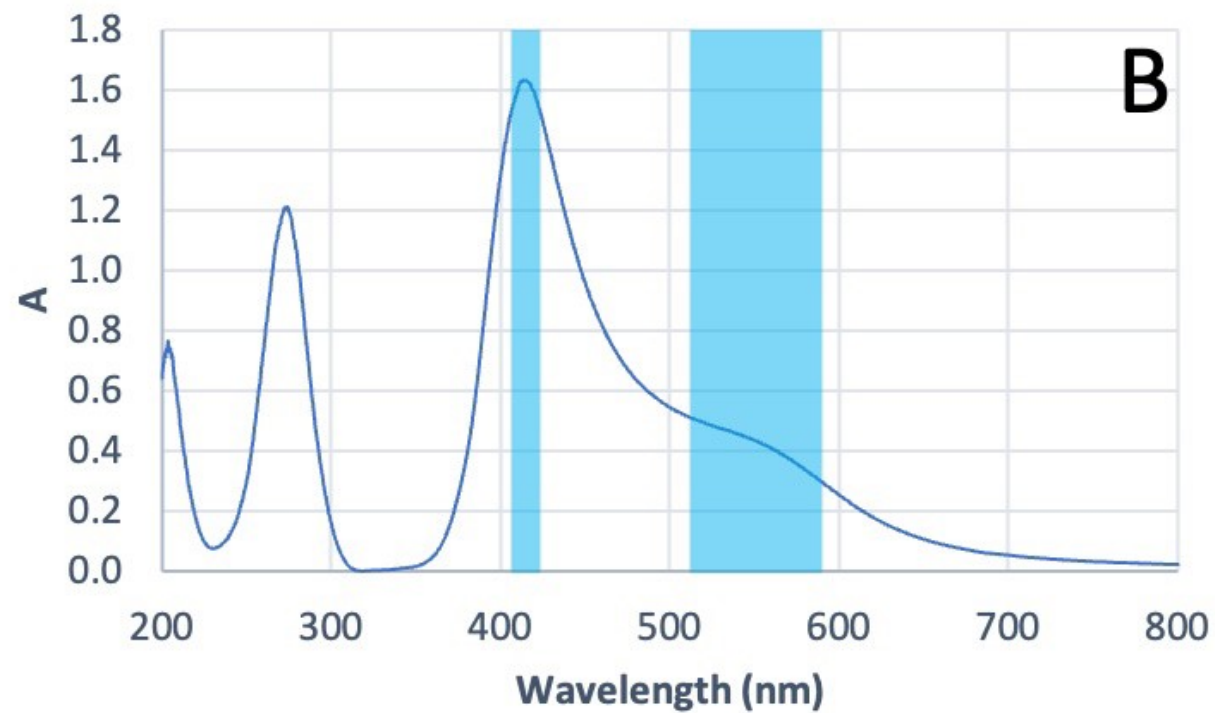

Supplement: RA-016-D5RA09570K-s017 [file RA-016-D5RA09570K-s017.pdf]

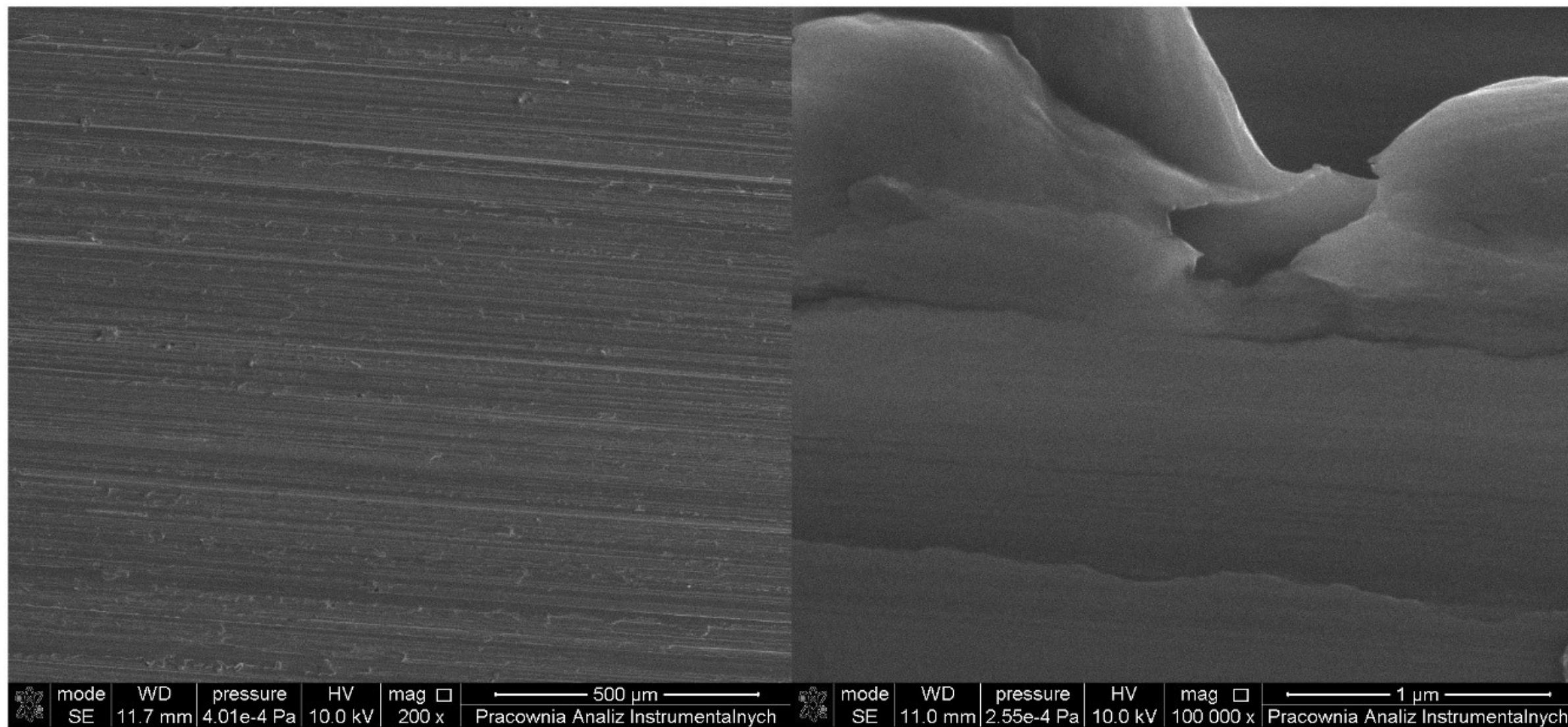

Supplement: RA-016-D5RA09570K-s018 [file RA-016-D5RA09570K-s018.pdf]

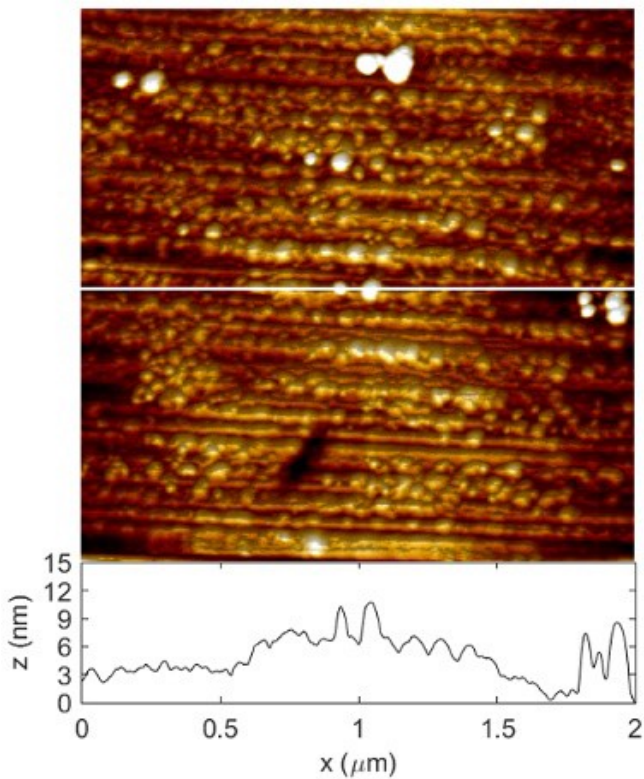

Supplement: RA-016-D5RA09570K-s019 [file RA-016-D5RA09570K-s019.pdf]

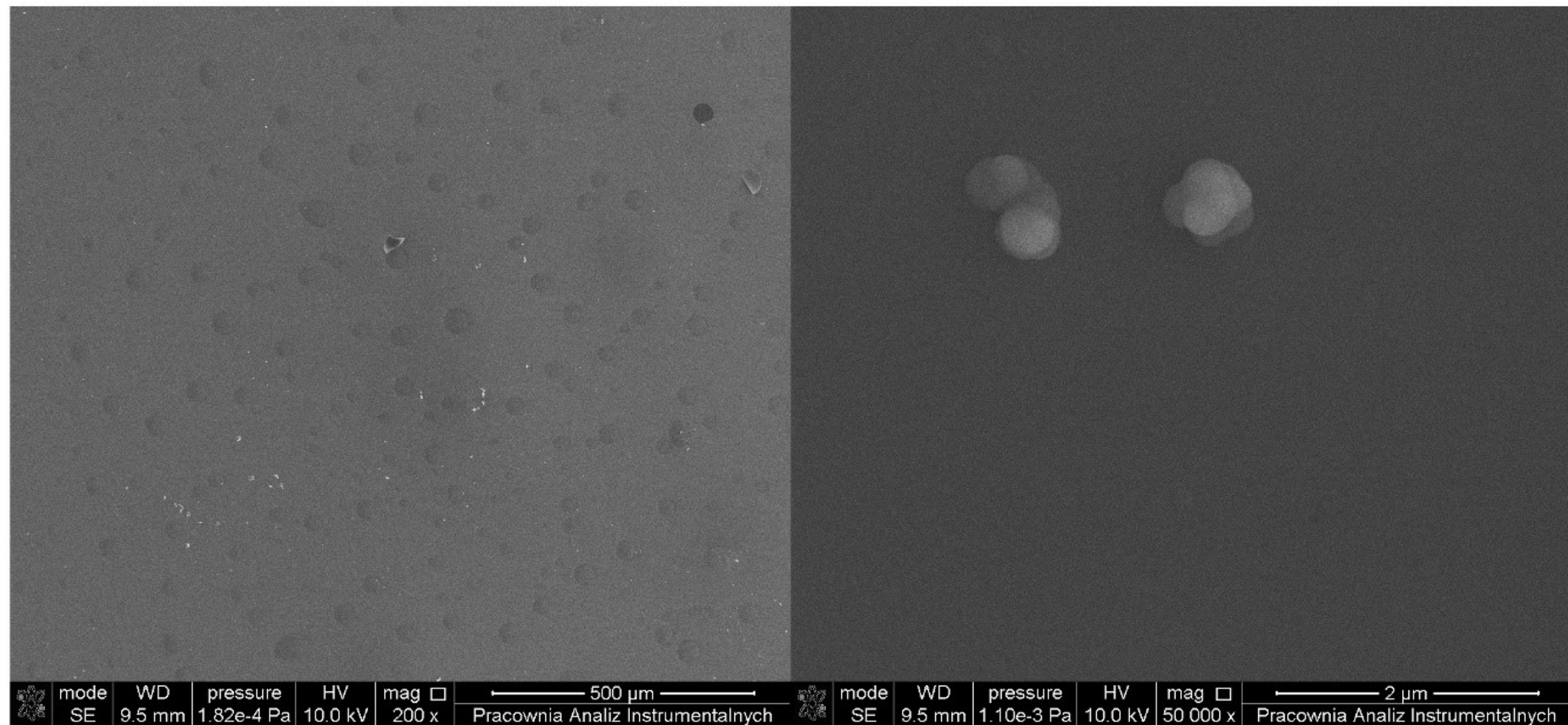

Supplement: RA-016-D5RA09570K-s020 [file RA-016-D5RA09570K-s020.pdf]

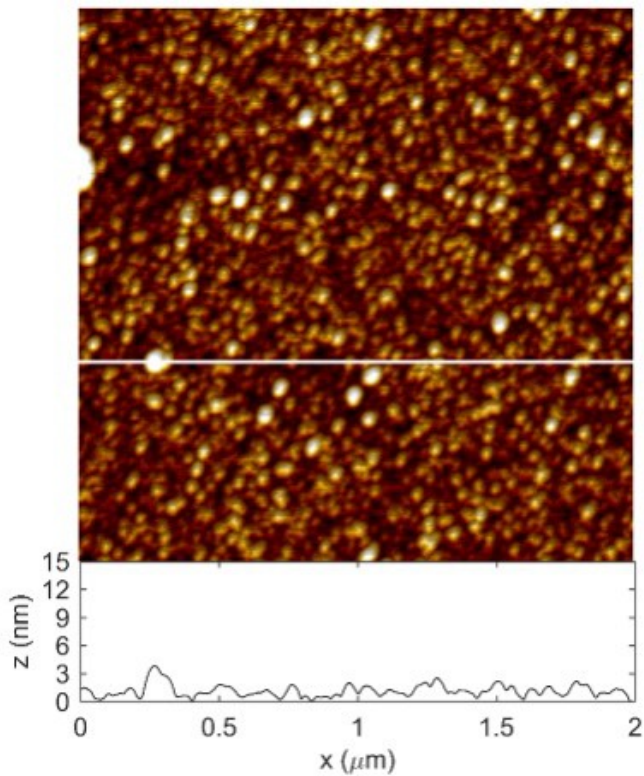

Supplement: RA-016-D5RA09570K-s021 [file RA-016-D5RA09570K-s021.pdf]
