# Supplementary material for: Al2O3/Ag nanostructured substrates prepared by ALD/CVD for matrix-free laser desorption/ionization mass spectrometry
Source: RSC Adv. 2026 May 20;16(29):27035–47. doi: 10.1039/d5ra09570k (PMC13191748; doi:10.1039/d5ra09570k)
Supplement: RA-016-D5RA09570K-s014 [file RA-016-D5RA09570K-s014.pdf]

Supplementary Information (SI) for RSC Advances.
This journal is © The Royal Society of Chemistry 2026

Supplementary Information

Cluster composition	Molecular weight	
	theoretical	observed
$^{107}Ag^{+}$	106.91	106.91
$^{109}Ag^{+}$	108.90	108.91
$^{107}Ag + Na^{+}$	129.89	129.90
$^{109}Ag + Na^{+}$	131.89	131.90
$^{107}Ag + Na^{+} + H_3O$	148.92	149.18
$^{109}Ag + Na^{+} + H_3O$	150.92	150.98
$^{107}Ag_2{}^{+}$	213.81	213.82
$[^{107}Ag + {}^{109}Ag]^{+}$	215.81	215.82
$^{109}Ag_2{}^{+}$	217.81	217.83
$^{107}Ag_2{}^{+} + NH_3 + H_2O$	248.86	248.85
$[^{107}Ag + {}^{109}Ag]^{+} + NH_3 + H_2O$	250.86	250.85
$^{109}Ag_2{}^{+} + NH_3 + H_2O$	252.86	252.85
$^{107}Ag_3{}^{+}$	320.72	320.73
$[^{107}Ag_2 + {}^{109}Ag]^{+}$	322.71	322.71
$[^{107}Ag + {}^{109}Ag_2]^{+}$	324.71	324.71
$^{109}Ag_3{}^{+}$	326.71	326.72
$^{107}Ag + {}^{109}Ag_2 + K^{+}$	363.71	363.30
$^{109}Ag^{3} + K^{+}$	365.81	365.30

Supplementary Table S1. Comparison of experimental and theoretical masses for ions originating from AgNPs obtained via CVD deposition.

Supplementary Note 1. Description of the custom-built hot-wall CVD reactor.

Silver nanoparticles were deposited in a custom-built hot-wall CVD reactor consisting of a horizontal glass reaction tube, two independently controlled heating zones, a gas-supply line, a vacuum line, and a liquid-nitrogen cold trap for volatile by-products. The first zone was used for precursor evaporation, whereas the second zone contained the substrate and served as the deposition/decomposition zone. Argon was used as the carrier gas during deposition, and hydrogen was introduced after deposition to assist in the removal of residual by-products and the formation of metallic silver.
The substrate was positioned on a dedicated glass holder in the deposition zone, while the precursor was placed in a separate vessel in the evaporation zone. This configuration enabled separation of precursor sublimation from its transport and decomposition in the region containing the heated substrate. During a single CVD process, nanoparticles were deposited onto one substrate. A schematic of the reactor is provided in **Supplementary Fig. S1.**

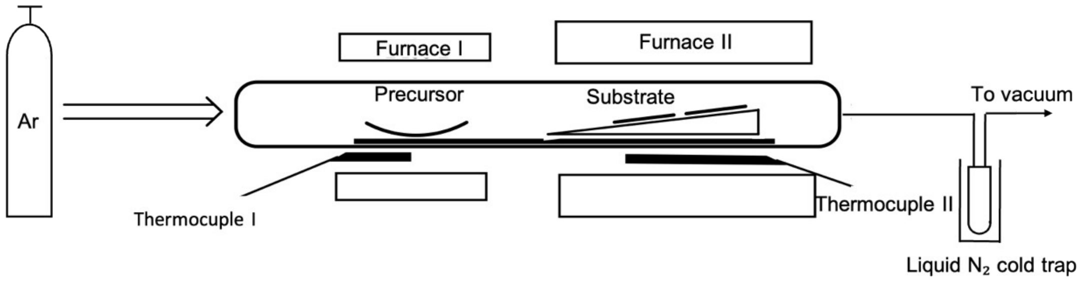

Supplementary Fig. S1. Schematic representation of the custom-built hot-wall CVD reactor used for Ag nanoparticle deposition.

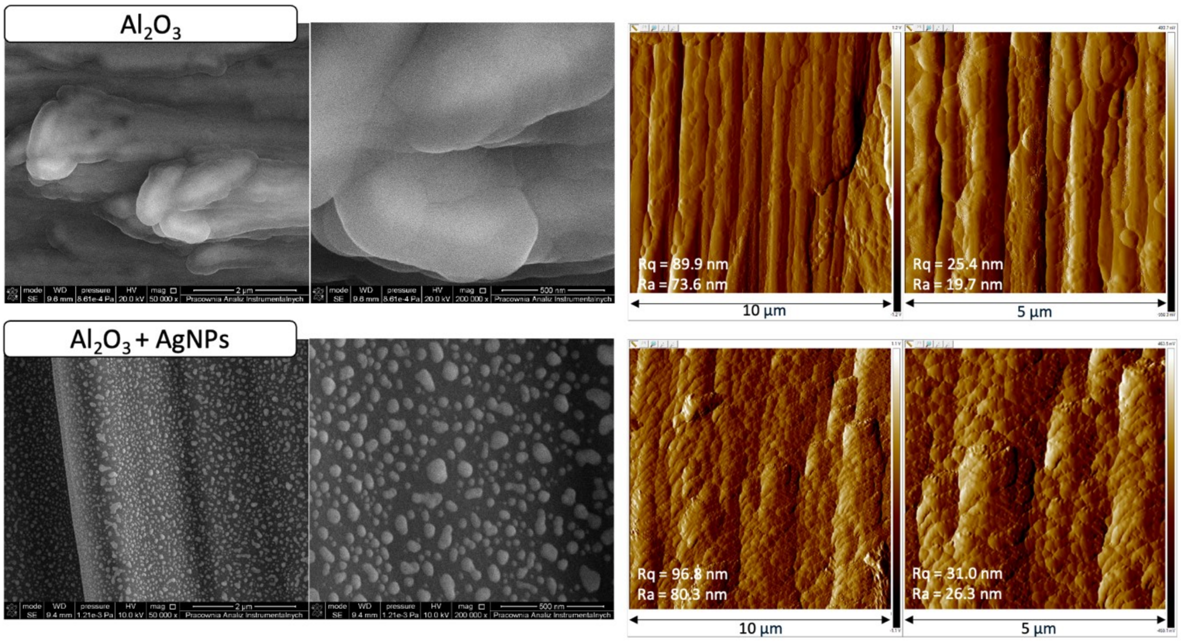

Supplementary Fig. S2. Comparison of the morphology and topography of NALDI plate no. 2. SEM images (50,000×, 200,000×) and AFM maps (10 µm, 5 µm) of the surface: top - Al_2O_3-coated steel substrate before Ag deposition, bottom - Al_2O_3 layer coated with silver nanoparticles (Al_2O_3 + AgNPs).

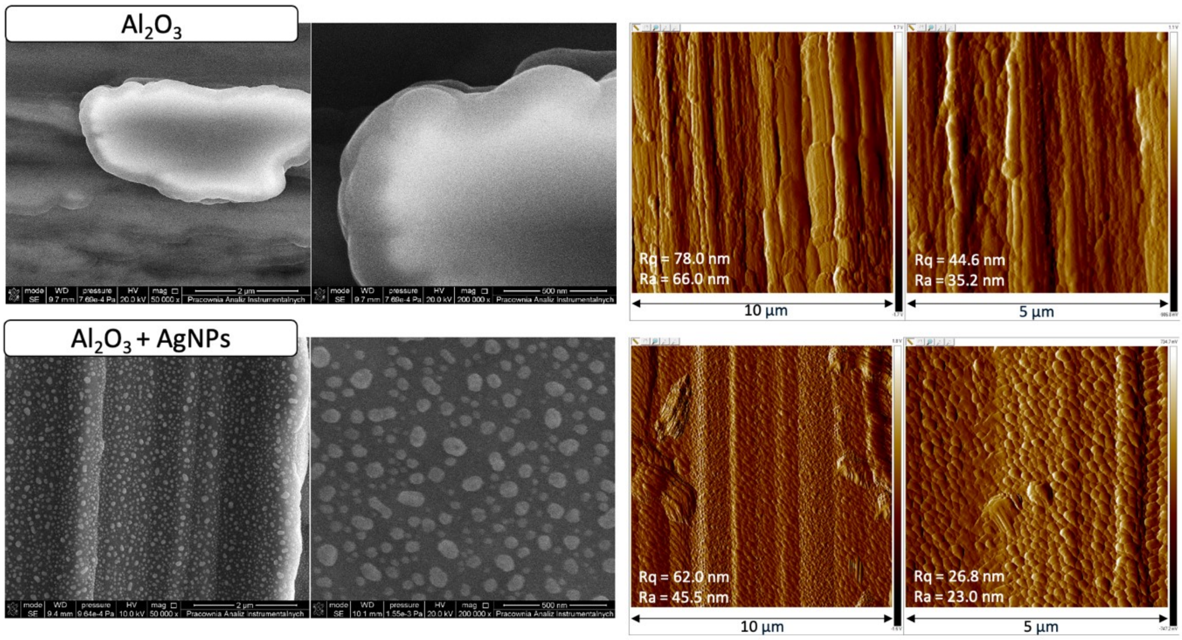

Supplementary Fig. S3. Comparison of the morphology and topography of NALDI plate no. 3. SEM images (50,000×, 200,000×) and AFM maps (10 µm, 5 µm) of the surface: top - Al_2O_3-coated steel substrate before Ag deposition, bottom - Al_2O_3 layer coated with silver nanoparticles (Al_2O_3 + AgNPs).

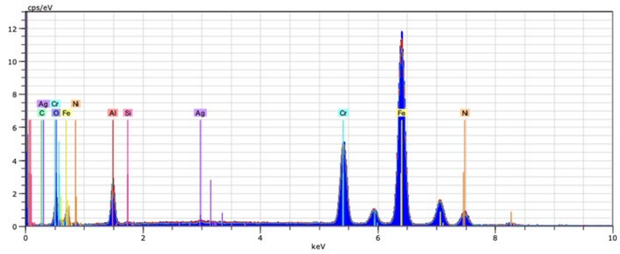

Atomic percent (%)

Spectrum	C	O	Al	Si	Cr	Fe	Ni	Ag
58719	2.80	11.48	14.28	0.38	14.19	51.34	5.21	0.32
58720	3.06	13.22	14.88	0.40	13.81	49.34	4.96	0.34
58721	2.87	14.05	14.62	0.42	13.24	48.98	5.54	0.27
58722	2.96	12.45	12.83	0.33	14.25	51.29	5.61	0.28
58723	3.28	13.96	14.26	0.48	13.63	48.89	5.22	0.29
Mean value:	2.99	13.03	14.18	0.40	13.82	49.97	5.31	0.30
Sigma:	0.19	1.08	0.79	0.05	0.42	1.24	0.27	0.03
Sigma mean:	0.08	0.48	0.35	0.02	0.19	0.55	0.12	0.01

Supplementary Fig. S4. EDX analysis of plate 2.

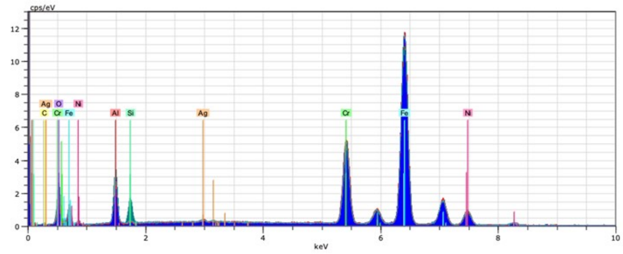

Atomic percent (%)

Spectrum	C	O	Al	Si	Cr	Fe	Ni	Ag
58714	3.21	14.31	12.18	0.43	13.83	50.14	5.61	0.30
58715	3.40	14.40	14.08	0.47	13.82	48.37	5.13	0.33
58716	3.21	19.59	15.28	6.37	11.36	39.79	4.10	0.30
58717	3.23	13.14	14.95	0.46	13.96	48.90	5.05	0.31
58718	3.15	14.53	13.76	0.43	13.62	48.93	5.26	0.31
Mean value:	3.24	15.19	14.05	1.63	13.32	47.23	5.03	0.31
Sigma:	0.09	2.52	1.22	2.65	1.10	4.21	0.56	0.01
Sigma mean:	0.04	1.13	0.54	1.18	0.49	1.88	0.25	0.00

Supplementary Fig. S5. EDX analysis of plate 3.

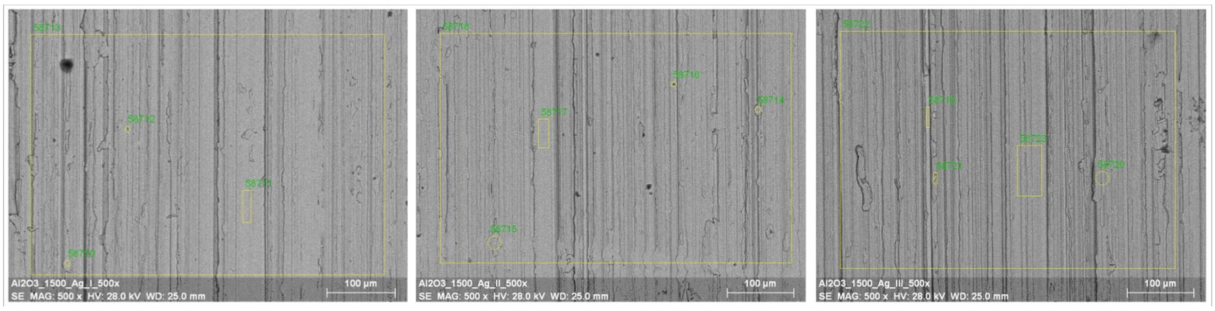

Supplementary Fig. S6. SEM images of the surface areas selected for EDX analysis for plates 1, 2, and 3. The marked points and areas correspond to the locations where the EDX measurements were performed.

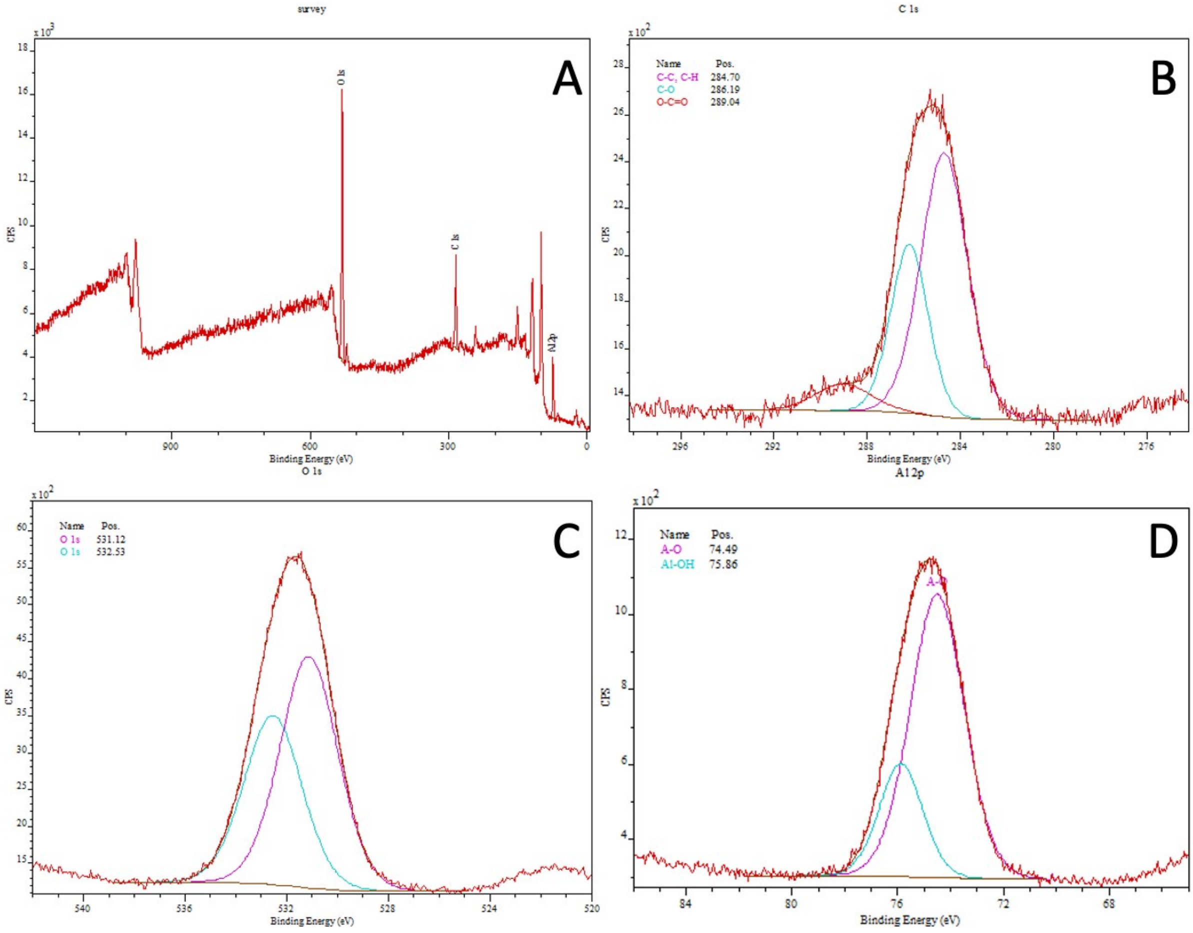

Supplementary Fig. S7. XPS characterization of the Al_2O_3 sample (plate 2): (A) survey spectrum; (B) O 1s high-resolution spectrum; (C) C 1s high-resolution spectrum; and (D) Al 2p high-resolution spectrum.

%Al	%O	%C
28.21	39.23	32.56

Supplementary Table S2. Surface elemental composition of the Al_2O_3 sample (plate 2) calculated from the XPS spectra.

Region	Binding energy (eV)	Assignment	Surface concentration (%)
C 1s	284.70	C-C/C=C	19.91
C 1s	286.19	C-O	10.21
C 1s	289.04	O-C=O	2.44
O 1s	531.12	metal oxides	22.65
O 1s	532.53	C-O-C/C-OH	16.58
Al 2p	74.49	Al-O	21.40
Al 2p	75.86	Al-OH	6.81

Supplementary Table S3. Relative surface concentrations of the components obtained from deconvolution of the XPS spectra of the Al_2O_3 sample (plate 2).

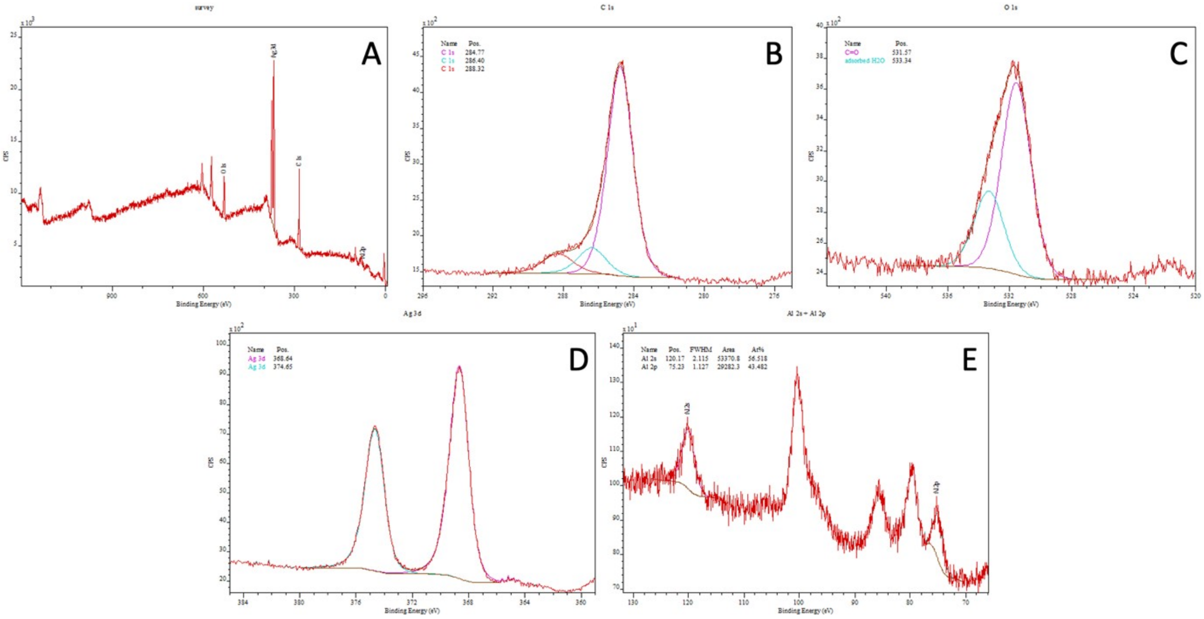

Supplementary Fig. S8. XPS characterization of the Al_2O_3 + Ag sample (plate 2): (A) survey spectrum; (B) Ag 3d high-resolution spectrum; (C) O 1s high-resolution spectrum; (D) C 1s high-resolution spectrum; and (E) Al 2s/2p region.

%C	%O	%Ag	%Al
64.26	17.66	12.23	5.85

Supplementary Table S4. Surface elemental composition of the Al_2O_3 + Ag sample (plate 2) calculated from the XPS spectra.

Region	Binding energy (eV)	Assignment	Surface concentration (%)
C 1s	284.77	C-C/C=C	51.43
C 1s	286.40	C-O/C-OH	7.03
C 1s	288.32	C=O	5.80
O 1s	531.57	C=O / oxygen-containing surface species	12.59
O 1s	533.34	adsorbed H_2O / adsorbed surface species	5.07
Ag 3d	368.64 / 374.65	Ag $3d_5/_2$ and Ag $3d_3/_2$, metallic Ag (Ag^0)	12.23
Al 2p	75.23	Al 2p Al_2O_3	5.85

Supplementary Table S5. Relative surface concentrations of the components obtained from deconvolution of the XPS spectra of the Al_2O_3 + Ag sample (plate 2).

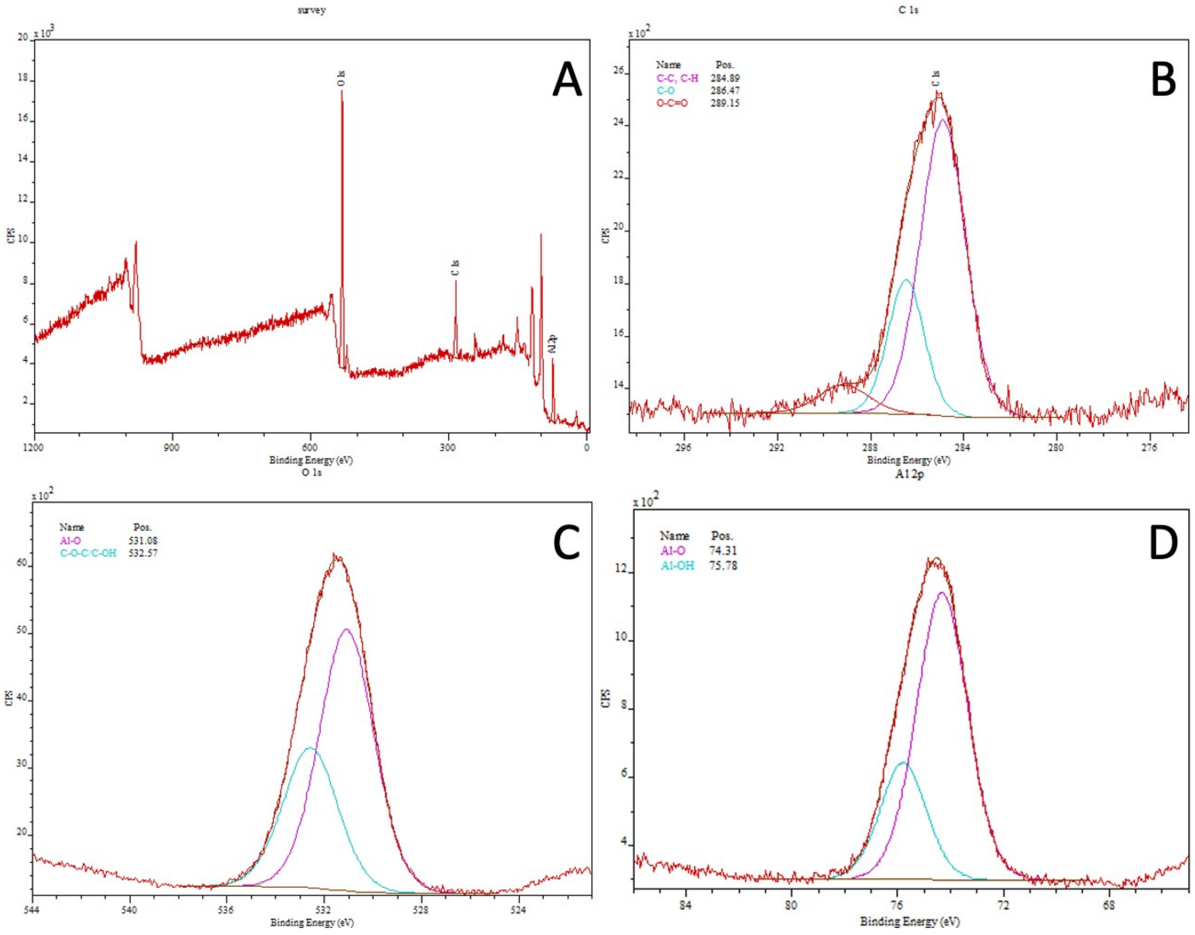

Supplementary Fig. S9. XPS characterization of the Al_2O_3 sample (plate 3): (A) survey spectrum; (B) O 1s high-resolution spectrum; (C) C 1s high-resolution spectrum; and (D) Al 2p high-resolution spectrum.

%Al	%O	%C
30.36	42.15	27.49

Supplementary Table S6. Surface elemental composition of the Al_2O_3 sample (plate 3) calculated from the XPS spectra.

Region	Binding energy (eV)	Assignment	Surface concentration (%)
C 1s	284.89	C-C/C=C	18.71
C 1s	286.47	C-O	6.80
C 1s	289.15	O-C=O	1.98
O 1s	531.08	metal oxides	27.40
O 1s	532.57	C-O-C/C-OH	14.75
Al 2p	74.31	Al-O	22.21
Al 2p	75.78	Al-OH	8.15

Supplementary Table S7. Relative surface concentrations of the components obtained from deconvolution of the XPS spectra of the Al_2O_3 sample (plate 3).

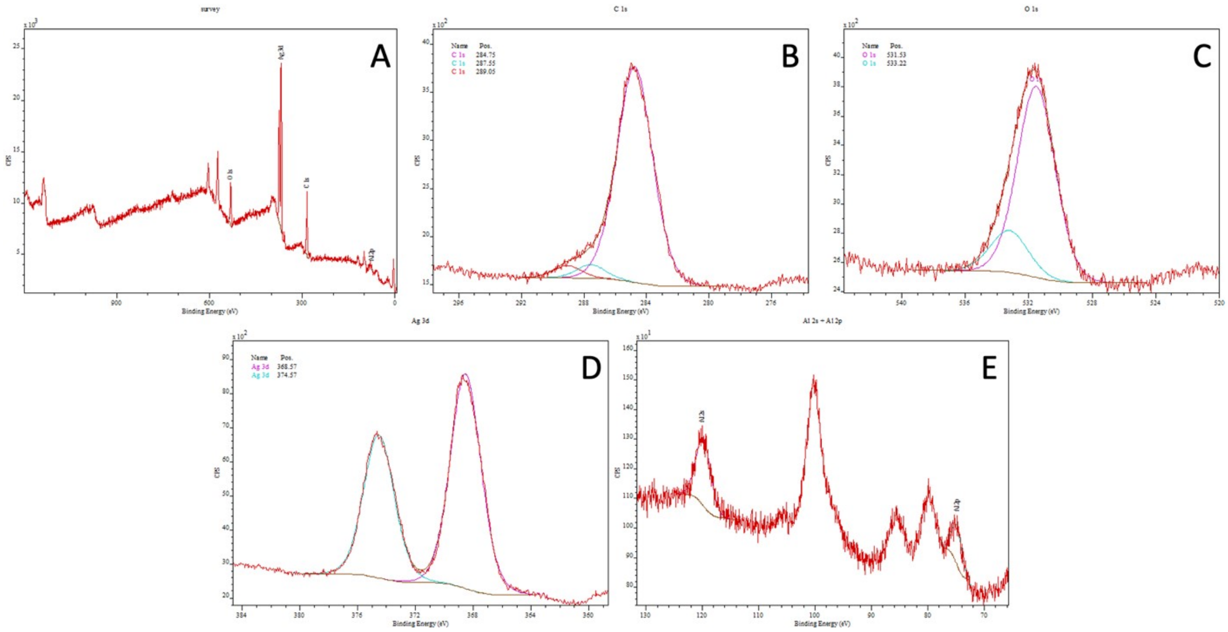

Supplementary Fig. S10. XPS characterization of the Al_2O_3 + Ag sample (plate 3): (A) survey spectrum; (B) Ag 3d high-resolution spectrum; (C) O 1s high-resolution spectrum; (D) C 1s high-resolution spectrum; and (E) Al 2s/2p region.

%C	%O	%Ag	%Al
65.06	18.59	13.30	3.05

Supplementary Table S8. Surface elemental composition of the Al_2O_3 + Ag sample (plate 3) calculated from the XPS spectra.

Region	Binding energy (eV)	Assignment	Surface concentration (%)
C 1s	284.75	C-C/C=C	59.39
C 1s	286.55	C-O/C-OH	2.99
C 1s	289.09	C=O	2.67
O 1s	531.53	C=O / oxygen-containing surface species	15.25
O 1s	532.22	adsorbed H_2O / adsorbed surface species	3.34
Ag 3d	368.57 / 374.57	Ag $3d_5/_2$ and Ag $3d_3/_2$, metallic Ag (Ag^0)	13.30
Al 2p	74.80	Al 2p Al_2O_3	3.05

Supplementary Table S9. Relative surface concentrations of the components obtained from deconvolution of the XPS spectra of the Al_2O_3 + Ag sample (plate 3).

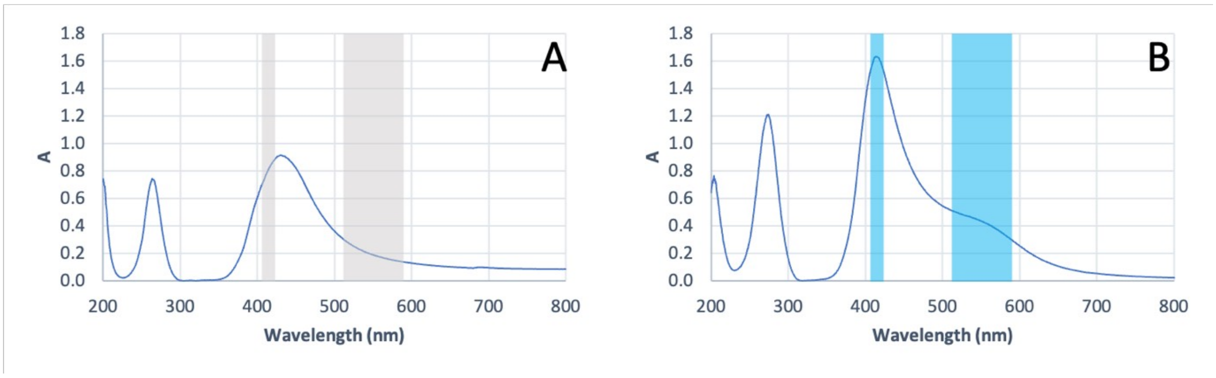

Supplementary Fig. S11. UV-Vis DRS spectra of samples (A) Al_2O_3 and (B) Al_2O_3 + AgNPs for plate 2.

Sample	Band Gap (eV)	λmax (nm)
Al_2O_3	2.55	200.5
	4.47	262.0
	6.01	426.0
Al_2O_3 + AgNPs	2.69	203.5
	4.24	272.5
	5.78	414.0

Supplementary Table S10. Calculated band gap values (Eg) and corresponding wavelengths (λmax) for the Al_2O_3 and Al_2O_3 + AgNPs samples for plate 2.

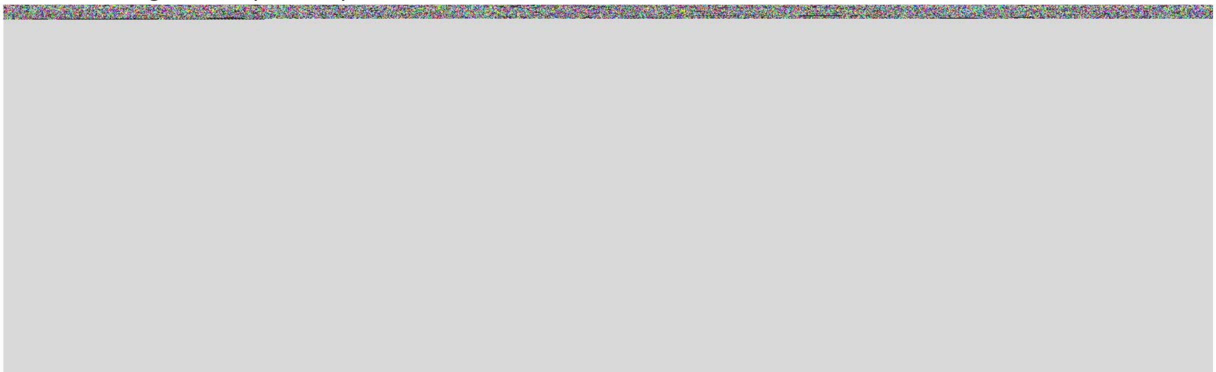

Supplementary Fig. S12. UV-Vis DRS spectra of samples (A) Al_2O_3 and (B) Al_2O_3 + AgNPs for plate 3.

Sample	Band Gap (eV)	λmax (nm)
Al_2O_3	2.50	201.5
	4.42	263.5
	5.94	431.0
Al_2O_3 + AgNPs	2.03	202.5
	2.78	269.5
	4.32	409.5

Supplementary Table S11. Calculated band gap values (Eg) and corresponding wavelengths (λmax) for the Al_2O_3 and Al_2O_3 + AgNPs samples for plate 3.

Supplementary Note 2. SEM characterization of the steel substrate

The image obtained at lower magnification reveals the presence of distinct, parallel topographic features on the substrate surface, consistent with prior mechanical treatment of the material. These structures give the surface a clearly ordered, directional character on the microscale. In turn, the image recorded at higher magnification shows that, in addition to the dominant linear traces, the surface also contains local morphological irregularities and protrusions. The SEM observations therefore indicate that the initial steel substrate is not ideally smooth or homogeneous, but instead exhibits a complex topography across different observation scales.

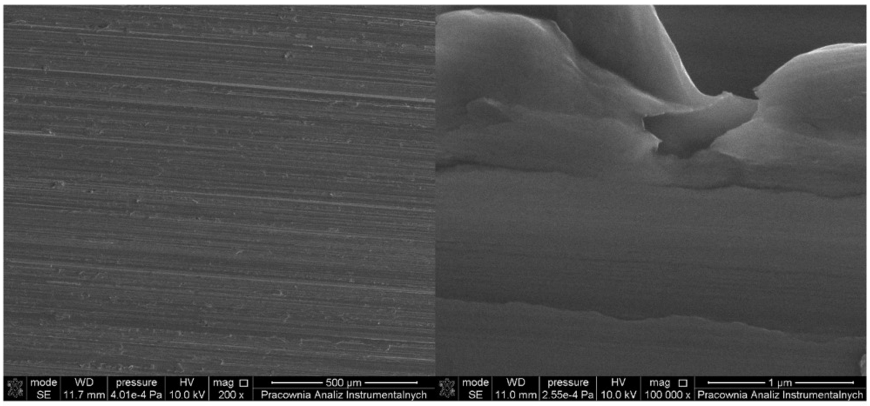

Supplementary Fig. S13. SEM images of the steel substrate surface before deposition of the Al_2O_3 layer.

Supplementary Note 3. AFM characterization of the steel substrate

Supplementary Fig. S14 presents the AFM image of the surface topography of stainless steel recorded over a scan area of 2 × 2 µm². As can be seen, the surface exhibits a developed and heterogeneous morphology with numerous irregular features of varying heights.

R_q	R_a	R_{max}
1.39 nm	1.01 nm	18.9 nm

Supplementary Table S12. Surface roughness parameters (R_a, R_q, R_{max}) of the stainless-steel substrate.

The calculated roughness parameters (**Supplementary Table S12**) are Ra = 1.01 nm and Rq = 1.39 nm, indicating a moderately developed surface. The maximum profile height (Rmax) reaches 18.9 nm, confirming the presence of local topographical peaks and significant height variations. These results indicate that the analyzed surface is not uniform and that its morphology may be related both to the surface-preparation procedure and to the intrinsic nature of the metallic substrate.

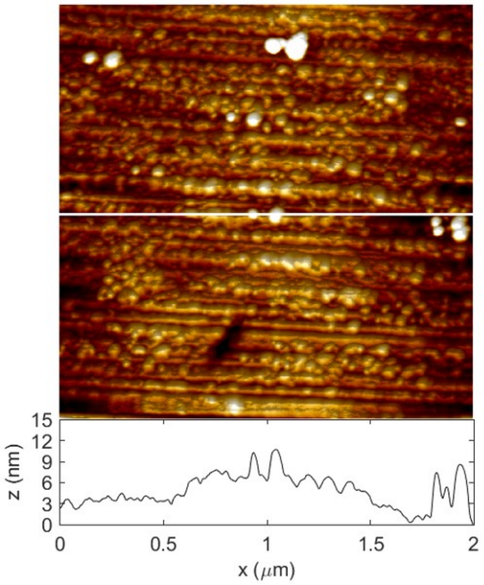

Supplementary Fig. S14. AFM image of the surface topography of stainless steel (scan area 2 × 2 µm²).

Supplementary Note 4. SEM characterization of the reference Al_2O_3 sample

SEM analysis of the reference sample (Si chip coated with an Al_2O_3 layer) showed that the surface was generally uniform within the analyzed area, although local morphological inhomogeneities were also present. At lower magnification, small, dispersed contrast features of similar morphology and distribution were visible over the surface. At higher magnification, the surface was composed predominantly of a relatively smooth background, with only a few isolated protrusion-like objects.

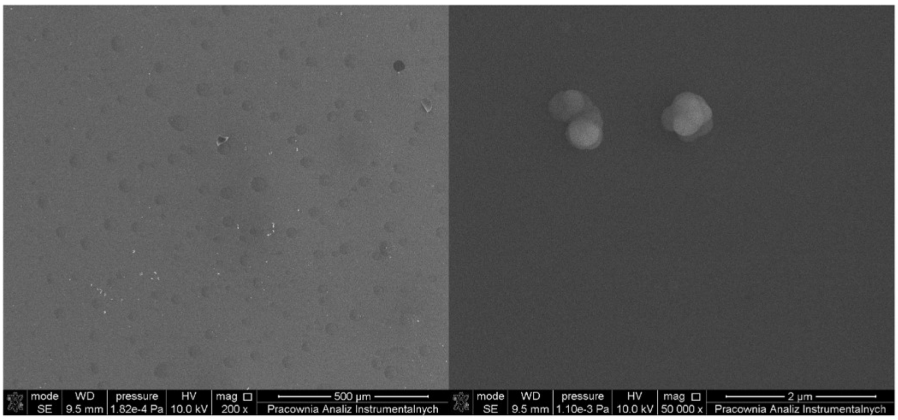

Supplementary Fig. S15. SEM images of the reference sample (Si chip coated with an Al_2O_3 layer) recorded at different magnifications.

Supplementary Note 5. AFM characterization of the reference Al_2O_3 sample

Supplementary Fig. S16 shows the AFM image of the surface topography of an Al_2O_3 thin film deposited on a silicon substrate using the atomic layer deposition (ALD) method, recorded over a scan area of 2 × 2 µm². As can be observed, the surface is highly uniform and does not exhibit pronounced large-scale features.

R_q	R_a	R_{max}
0.489 nm	0.377 nm	6.01 nm

Supplementary Table S13. Surface roughness parameters (R_a, R_q, R_{max}) of Al_2O_3 thin film deposited on a Si substrate.

The low roughness values (Ra = 0.377 nm, Rq = 0.489 nm) are consistent with a smooth and uniform ALD-grown Al_2O_3 film on Si. In the context of the present study, this reference sample serves primarily as a comparative system showing that the much stronger topographic heterogeneity observed for the steel-supported samples is substrate-related rather than intrinsic to the ALD process itself.

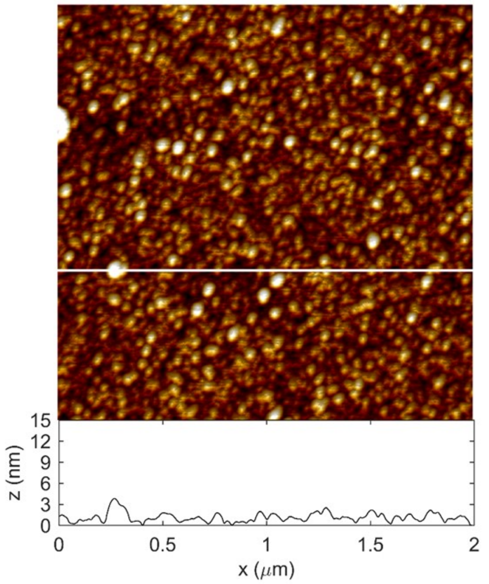

Supplementary Fig. S16. AFM image of the surface topography of an Al_2O_3 thin film deposited on a Si substrate by atomic layer deposition (ALD) (scan area 2 × 2 µm²).

Supplementary Note 6. EDX characterization of the reference Al_2O_3 sample

EDX analysis and elemental mapping of the reference sample detected Si, O, and Al within the analyzed area. The elemental distribution maps showed signals corresponding to these elements throughout the analyzed field of view. The quantitative atomic composition determined on the basis of EDX analysis is presented in Supplementary **Figs. S17 and S18**.

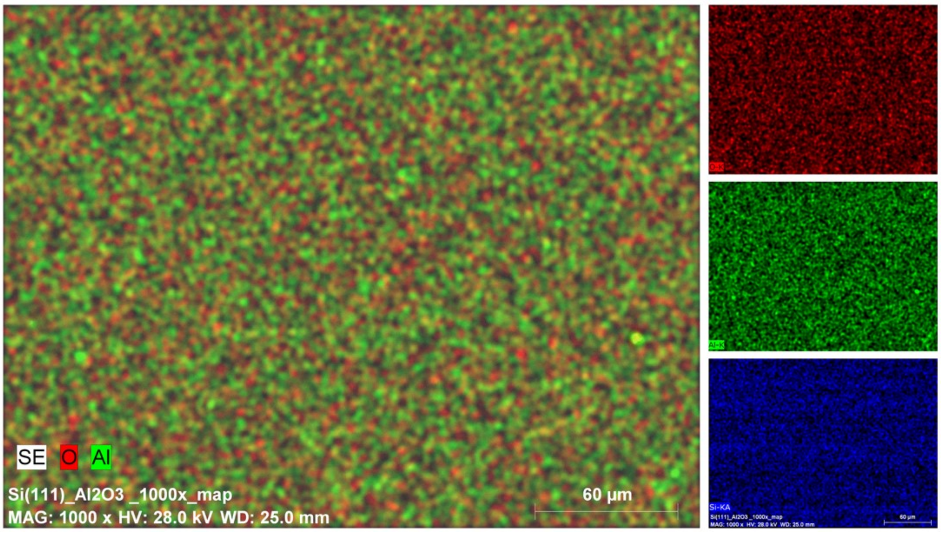

Supplementary Fig. S17. EDX elemental mapping of the reference sample (Si chip coated with an Al_2O_3 layer), including: (A) composite elemental map, (B) O map, (C) Al map, and (D) Si map.

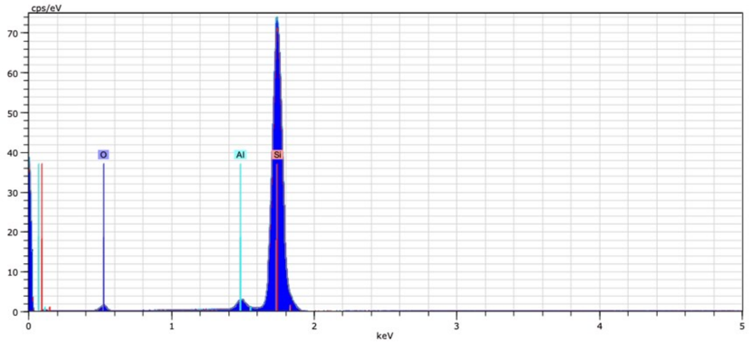

Atomic percent (%)

Spectrum	O	Al	Si
65201	26.71	2.35	70.94
65202	25.92	2.46	71.63
65203	26.25	2.39	71.36
Mean value:	26.29	2.40	71.31
Sigma:	0.40	0.05	0.35
Sigma mean:	0.23	0.03	0.20

Supplementary Fig. S18. EDX spectrum and average atomic composition of the reference sample (Si chip coated with an Al_2O_3 layer).

Supplementary Note 7. XPS characterization of the reference sample

XPS analysis of the reference Al_2O_3/Si sample revealed only O, Al, and C signals. The lower carbon contribution observed for this sample, relative to the steel-supported specimens, supports the interpretation that the elevated carbon content measured on the latter is dominated by surface contamination and adsorption effects. The Al 2p and O 1s components are consistent with Al-O and surface-hydroxylated alumina species, in agreement with the expected surface chemistry of ALD-grown Al_2O_3.

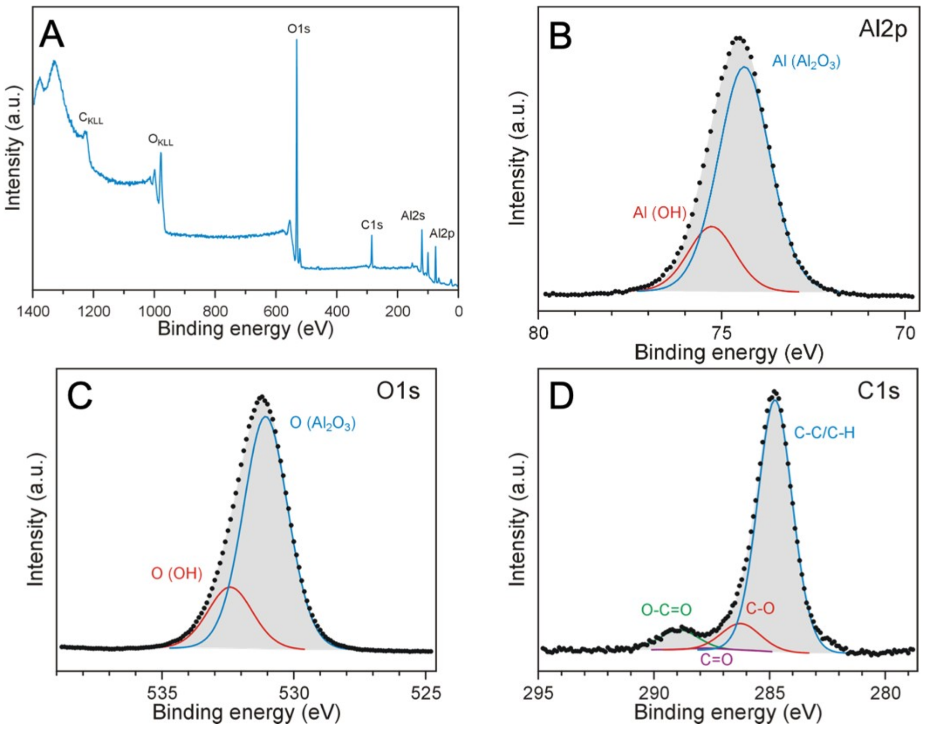

Supplementary Fig. S19. XPS spectra of the reference sample: survey spectrum and high-resolution Al 2p, O 1s, and C 1s spectra.

%Al	%O	%C
34.63	47.60	17.77

Supplementary Table S14. Surface elemental composition of the reference sample calculated from the XPS spectra.

O1	O2	C1	C2	C3	C4	Al1	Al2
37.67	9.93	14.59	1.75	0.12	1.31	27.48	7.15

Supplementary Table S15. Relative surface concentrations of the components obtained from deconvolution of the XPS spectra of the reference sample.

Bibliography

[1] S. Virtanen, I. Milošev, E. Gomez-Barrena, R. Trebše, J. Salo, and Y. T. Konttinen, "Special modes of corrosion under physiological and simulated physiological conditions," *Acta Biomater.*, vol. 4, no. 3, pp. 468–476, May 2008, doi: 10.1016/j.actbio.2007.12.003.

[2] S. M. George, "Atomic Layer Deposition: An Overview," *Chem. Rev.*, vol. 110, no. 1, pp. 111–131, Jan. 2010, doi: 10.1021/cr900056b.

[3] R. L. Puurunen, "Surface chemistry of atomic layer deposition: A case study for the trimethylaluminum/water process," *J. Appl. Phys.*, vol. 97, no. 12, Jun. 2005, doi: 10.1063/1.1940727.

[4] J. F. Moulder, *Handbook of X-Ray Photoelectron Spectroscopy*. 1992.
